# Person–environment interaction processes in frailty: a scoping review

**DOI:** 10.1093/geront/gnag044

**Published:** 2026-04-06

**Authors:** Junjie Zhang, Xiayu Wang, Paul S F Yip

**Affiliations:** Department of Social Work and Social Administration, The University of Hong Kong, Hong Kong SAR, China; School of Sociology, China University of Political Science and Law, Beijing, China; Department of Social Work and Social Administration, The University of Hong Kong, Hong Kong SAR, China; Hong Kong Jockey Club Centre for Suicide Research and Prevention, The University of Hong Kong, Hong Kong SAR, China

**Keywords:** Frailty, Person–environment interaction, Environmental stress, Adaptation

## Abstract

**Background and Objective:**

Frailty is an age-related syndrome characterized by increased vulnerability to stressors and adverse health outcomes. The environment influences older adults across many dimensions, yet person–environment (P–E) interactions related to frailty remain underexplored. This review aims to synthesize knowledge on P–E interactions associated with frailty.

**Research Design and Methods:**

We searched PubMed, Web of Science, and CINAHL for studies published between January 2001 and December 2024. Extracted data included study characteristics, definitions of environment and frailty, conceptual models, and key findings.

**Results:**

From 2,991 articles, 130 met inclusion criteria. Findings highlight the COntext Dynamics in Aging (CODA) framework’s relevance to frailty research. We identified a variety of environmental indicators across the CODA domains in these studies. Furthermore, we propose a refined model illustrating the dynamic interplay between individual adaptation and environmental stress. This model introduces a spatial-differential perspective, the “hierarchical stair” principle, where meeting needs at proximal levels enables engagement at broader spatial scales. The model also suggests that adaptation strategies evolve as individuals transition from proximal to more distal environments, with the strongest effects occurring at the home level.

**Discussion and Implications:**

This review emphasizes the dynamic interaction between individual adaptation and environmental stress across spatial levels. However, the review is limited by the scarcity of studies explicitly examining these interaction processes and by the lack of empirical evidence on spatial dynamics. Therefore, the proposed model provides a heuristic framework for hypothesis‑driven research, and future studies should prioritize empirical testing of this multilevel approach.

Frailty is a geriatric syndrome whose prevalence increases with age. It is commonly conceptualized using two leading models: the physical frailty model by Fried et al. (2001) and the deficit accumulation model by Mitnitski et al. (2001). The physical frailty model highlights the physical dimension of aging-related decline (Dent et al., 2016). In contrast, the deficit accumulation model views frailty as the cumulative impact of health deficits across multiple domains over the life course. Regardless of the model, frailty is consistently associated with a range of adverse health outcomes (Dent et al., 2019). Clarifying why frailty develops and which factors shape its progression is crucial for designing effective interventions and improving quality of life in later life.

Previous research has identified multiple factors associated with the presence and progression of frailty, particularly individual sociodemographic characteristics such as age, gender, and education, as well as physical factors including body weight and functional mobility (Feng et al., 2017; Kojima et al., 2018; Welstead et al., 2022). However, in the context of rapid societal mega-trends, driven by accelerating technological development, shifting political economies, and climate change (Rowles & Cutchin, 2025), environmental influences on frailty have received comparatively less attention (Fritz et al., 2020). Because frailty is closely tied to older adults’ capacity to adapt to environmental changes as they age (Dent et al., 2019), there is an increasing need to understand how environmental determinants contribute to frailty.

The study of environment in aging is continually evolving. Environment can be broadly defined as the totality of external phenomena, events, and forces that lie outside the individual (Dannefer, 2014). Foundational research such as Lawton’s Environmental Docility Hypothesis (Lawton & Simon, 1968) and Cantor’s life-space studies (1975) established key concepts for understanding how individual competence interacts with environmental impacts to shape mobility and well-being (Oswald et al., 2024; Wahl & Gerstorf, 2020). To integrate and extend this work, Wahl and Gerstorf (2018) proposed the COntext Dynamics in Aging (CODA) framework. The CODA framework distinguishes five dimensions of environment: socioeconomic, social, physical, care and service, and technology, alongside three key person–environment (P–E) processes: belonging, agency, and stress (Chaudhury & Oswald, 2019).

However, studies explicitly linking frailty to the CODA framework remain rare. While some reviews address physical or social environments in frailty (Fritz et al., 2020), few integrate these key concepts. Niedoba and Oswald’s (2024) review, the only one directly applying the CODA framework, shows that individuals with dementia actively interact with their environments in adaptive ways. A PROSPERO search revealed no existing systematic or scoping reviews addressing the CODA framework or P–E processes in frailty research. Given the fragmented and limited application of the CODA framework in frailty research, a scoping review is warranted to systematically map how the CODA framework’s environments and P–E processes have been conceptualized, and to identify gaps for future investigation. A scoping review protocol was registered on OSF (https://doi.org/10.17605/OSF.IO/4P83E) on December 3, 2024, prior to the search process.

Overall review question:

What is the relationship between frailty and the multidimensional environment including socioeconomic, social, physical, care and service, and technological domains as characterized in the CODA framework?

Subreview questions:

Which environmental components of the CODA framework have been studied in frailty research?

What person–environment interaction processes have been identified in the frailty research?

How are these person–environment interaction processes described, interpreted, or theorized in the literature?

## Methods

### Eligibility criteria

A PRISMA-ScR (Tricco et al., 2018) statement for this scoping review is provided in the Supplementary Materials. The PCC framework (Population, Concept, Context) guided the definition of key concepts and inclusion criteria (Peters et al., 2020). A detailed description of inclusion and exclusion criteria is provided in the Supplementary Materials. The review population consists of individuals with frailty or pre-frailty, irrespective of the conceptual models or the measurement tools used. We argue that the concept of frailty, distinct from conditions such as sarcopenia or disability, represents a unique geriatric syndrome. This conceptual distinction justifies the inclusive approach. Yet, we acknowledge that methodological heterogeneity may arise from differing theoretical frameworks. To address this, we documented measurement variability and accounted for methodological differences when interpreting findings. Studies were excluded if they referred to their sample as frail but only assessed physical function, disability, or comorbidity, because these conditions do not equate to frailty.

The concept of interest is the set of P–E interaction processes related to frailty. Environment encompasses multiple domains, including the socioeconomic, social, physical, care and service, and technological, as described in the CODA framework. According to Wahl and Gerstorf (2018), the socioeconomic domain refers to financial factors such as wealth, poverty, and employment at the neighborhood and state levels; the social domain encompasses relationships, social ties, community support, cohesion, and the broader culture of everyday interactions; the physical domain includes built and natural surroundings, housing conditions, and neighborhood infrastructure; the care and service domain covers the availability, quality, and accessibility of health‑related services and facilities; and the technological domain concerns the use and accessibility of technology. The interaction processes are agency, belonging, and stress. Agency refers to intentional and proactive behaviors, experiences, and cognitions through which individuals interact with and shape their environments. Belonging denotes the sense of security, safety, and emotional attachment that individuals form with their environments. Stress refers to the adaptive challenges that arise when the ability to exert agency or experience belonging is diminished in later life (Wahl & Gerstorf, 2018, 2020).

This scoping review focuses on P–E interactions among community-dwelling older adults and excludes studies of individuals in institutional or assisted settings, such as hospitals, nursing homes, and long‑term care facilities. Community-dwelling older adults are the primary target of aging-in-place policies and interventions, interacting with diverse environments that influence frailty risk (Collard et al., 2012; Duppen et al., 2019; Fritz et al., 2020). By concentrating on this population, the review aims to identify key emerging findings and to provide clearer insights into environmental influences on frailty.

### Information sources and search strategy

A literature search was conducted in December 2024 in three databases: PubMed, Web of Science, and CINAHL. The search was limited to studies published on or after January 1, 2001. This starting date was selected because landmark studies on frailty using the physical frailty model and the deficit accumulation model were published in 2001 (Fried et al., 2001; Mitnitski et al., 2001). The reference lists of the selected articles were searched for additional studies. Prior to the review, the first author conducted preliminary searches to compile a list of search terms aligned with the research objectives. The search was performed using titles and abstracts, and the specific search terms used are detailed in the Supplementary Materials. To balance methodological rigor with feasibility, this scoping review excluded nonpeer-reviewed and non-English studies.

### Selection and data collection process

The identified literature was uploaded into Covidence, and duplicates were removed. The first author developed the inclusion and exclusion criteria used to evaluate the studies. The process began with screening the titles and abstracts of potential publications. Full texts were retrieved for those that met the inclusion criteria. The first and second authors independently conducted the initial screening, resolving any conflicts regarding article inclusion through consensus. Data from the included studies were extracted using a standardized template. The items extracted are listed in the Supplementary Materials. Due to growing familiarity with the literature, the extracted items were modified during the extraction process. To assess the quality and risk of bias, we used the eight-item Newcastle–Ottawa Scale for cohort studies (Wells et al., 2000) and the eight-item Joanna Briggs Institute Critical Appraisal Checklist for Analytical Cross-Sectional Studies (Joanna Briggs Institute, 2017). Following criteria established in previous literature (Kojima et al., 2020), publications that scored five or fewer points were excluded from the review. For qualitative studies, we evaluated whether they provided a clear description of the study population, used a validated measure of frailty, specified the relevant environmental domain, and discussed the P–E interaction process.

### Data analysis method

The CODA framework highlights that P–E interactions involve multiple components and dynamic processes. The initial aim was to identify and illustrate specific P–E processes that link environmental factors to frailty, similar to the approach used by Niedoba and Oswald (2024) to study transitions into dementia. However, during the review, we found that most studies examined associations between environmental components and frailty without explicitly detailing the underlying P–E processes. Given this limitation, we adjusted the approach. Instead of focusing solely on mapping P–E processes, we collected and synthesized the theoretical frameworks cited in the selected studies along with their key findings. This allowed us to integrate multiple perspectives and theories related to frailty and the environment. We also mapped how studies defined environmental components in terms of their attributes, scale, and measurement, and how they conceptualized the pathways through which these components interact with frailty.

## Results

### Study characteristics

**Figure 1:**
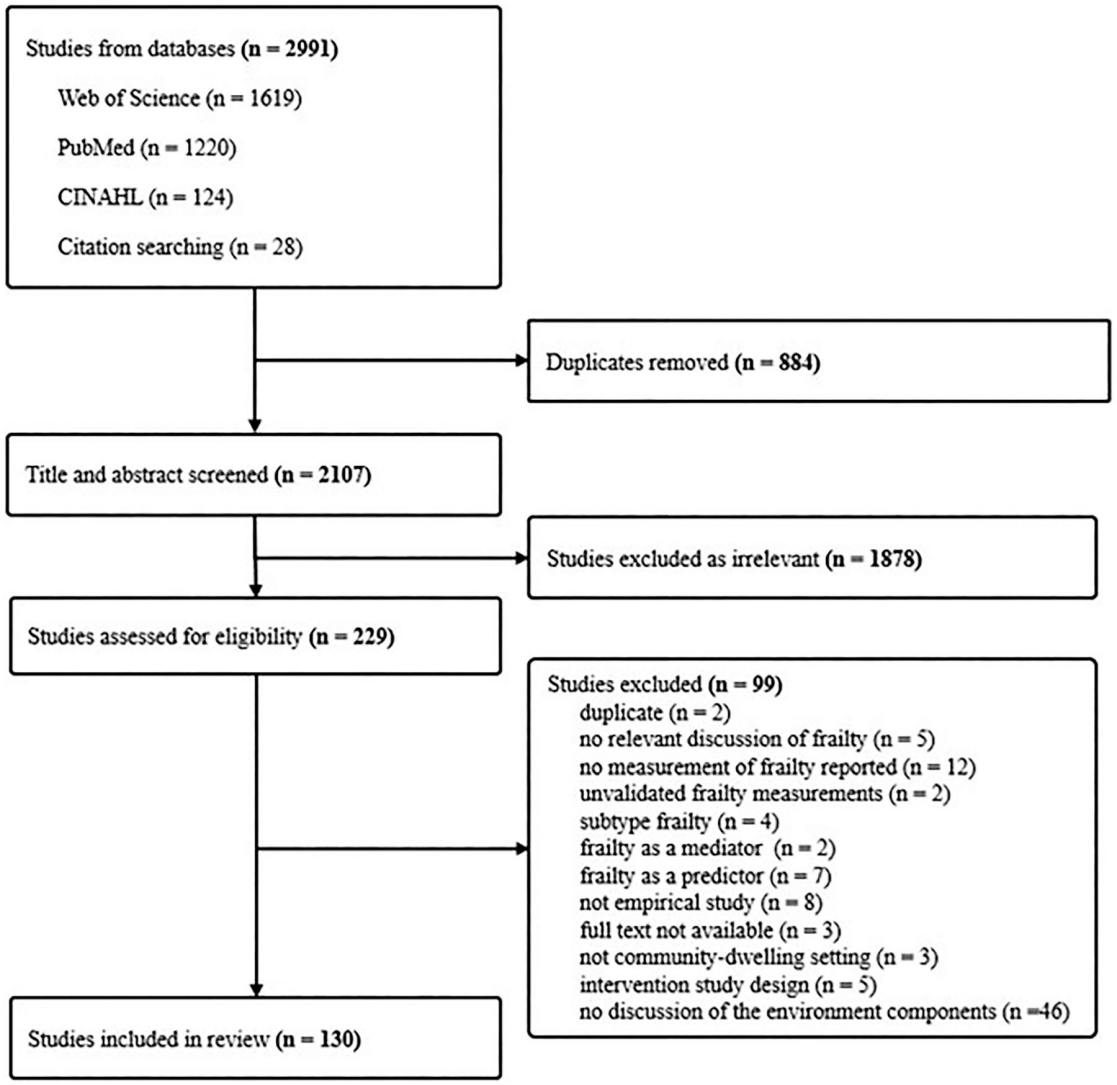
Sample selection procedure (adapted from PRISMA 2020 flow diagram).

Figure 1 shows the study selection process. The database and citation searches retrieved a total of 2,991 records, and 130 studies were included in the final analytical sample. Table 1 presents the study characteristics, including year of publication, country or region of origin, study design, and environmental dimension for the included studies. The studies were published between 2005 and 2024. By continent, most studies were conducted in Asia (*n* = 69), followed by Europe (*n* = 35), North America (*n* = 18), Africa (*n* = 1), South America (*n* = 3), and Australia (*n* = 4). Fifty-five percent of the selected studies used a cross-sectional design, while 34% employed a longitudinal design. Additionally, six studies were systematic reviews, six utilized a mixed design, and two were qualitative. Details of sample characteristics for the selected studies are presented in the Supplementary Materials. Sample sizes ranged from 13 participants (Thinuan et al., 2020) to 220,079 participants (Veronese et al., 2023). The percentage of female participants varied from 33% (Aranda et al., 2011) to 100% (Garcia-Vigara et al., 2022; Gardiner et al., 2016; Szanton et al., 2010). Mean ages ranged from 55.0 years (Schwartz et al., 2023) to 88.4 years (Chen, 2024). Regarding quality and risk of bias, the quantitative studies scored between six and eight on the appraisal scales (mean = 7.0). For reviews and qualitative studies, methodological quality was judged through consensus discussions between the first and second authors. Overall, all included studies were considered to have adequate methodological quality.

**Table 1:** Study characteristics.

Study characteristics (*N* = 130)	Count, *n* (%)
**Year of publication**	
2005–2010	3 (2.3)
2011–2016	18 (13.8)
2017–2022	68 (52.3)
2023–2024	41 (31.5)
**Continent**	
Asia	69 (53.1)
Europe	35 (26.9)
North America	18 (13.8)
Australia	4 (3)
South America	3 (2.3)
Africa	1 (0.8)
**Design**	
Qualitative	2 (1.5)
Cross-sectional	72 (55.4)
Prospective	44 (33.8)
Mixed methods	6 (4.6)
Systematic review	6 (4.6)
**Frailty**	
Deficit accumulation model	40 (30.7)
Physical frailty model	58 (44.6)
Multidomain model	25 (19.2)
Mixed frailty measures	7 (5.4)
**Dimension of environment**	
Social and physical environment	9 (6.9)
Social environment	36 (27.7)
Physical environment	45 (34.6)
Technological environment	5 (3.8)
Care/service environment	2 (1.5)
Socioeconomic environment	29 (22.3)
Socioeconomic and social/physical environment or care/service	4 (3.1)

*Note.* “Mixed frailty measures” indicates studies that used more than one type of frailty measure.

Characteristics of frailty measurements used in the selected studies are presented in the Supplementary Materials. Frailty is measured using three types of assessments: the frailty phenotype, which focuses on physical attributes; deficit accumulation measures, which evaluate health deficits across one or more domains; and multidomain measures, which integrate physical, psychological, and social factors. Fifty‑eight studies (45%) used frailty measurements based on the physical frailty model, while 40 studies (31%) utilized measures from the deficit accumulation model. Additionally, 25 studies focused on the multidomain frailty model. For the studies utilizing the deficit accumulation model, the number of deficits varied from 20 (Cao et al., 2022) to 62 (Woo et al., 2005), with an average of 42 deficits. For measurements based on the physical frailty model, the primary framework was the frailty phenotype developed by Fried et al. (2001), followed by a simplified version introduced by Morley et al. (2012). Several studies modified these physical frailty measures: Aranda et al. (2011) used a four‑item version of the Fried frailty phenotype focusing on weight loss, exhaustion, walking speed, and grip strength; Keranen et al. (2017) used a three‑item measure known as the Study of Osteoporotic Fractures Index; Chen et al. (2015) reported that their physical phenotypes were assessed objectively using a tri‑axial accelerometer; and Aravantinou‑Karlatou et al. (2022) and Christensen et al. (2024) employed a frailty phenotype measure customized for the Survey of Health, Ageing and Retirement in Europe (SHARE) dataset.

The selected studies also used a range of multidomain frailty measures, with three of the most frequently used being the Tilburg Frailty Indicator developed by Gobbens et al. (2012), the Kihon Checklist by Arai and Satake (2015), and the Kaigo‑Yobo List developed by Shinkai et al. (2013). Other tools included the Edmonton Frail Scale (Mümken et al., 2024), the Groningen Frailty Indicator (Shah et al., 2021), the Korean Frailty Scale, the China Frailty Scale, the Identification of Seniors at Risk of Functional Loss Scale, the Clinical Frailty Scale, and the Comprehensive Frailty Assessment Instrument.

### Findings categorized by the CODA framework

**Figure 2:**
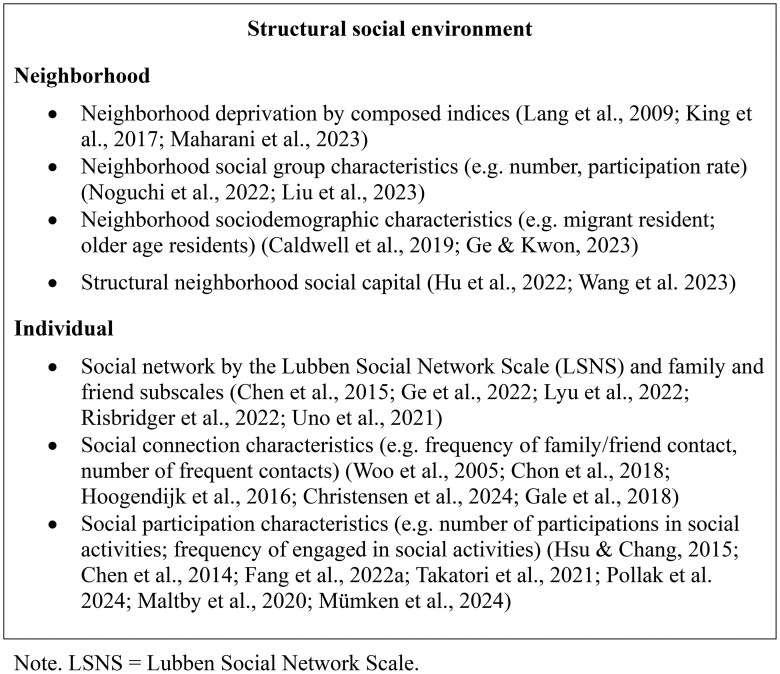
Structural social environment measures in selected studies.

**Figure 3:**
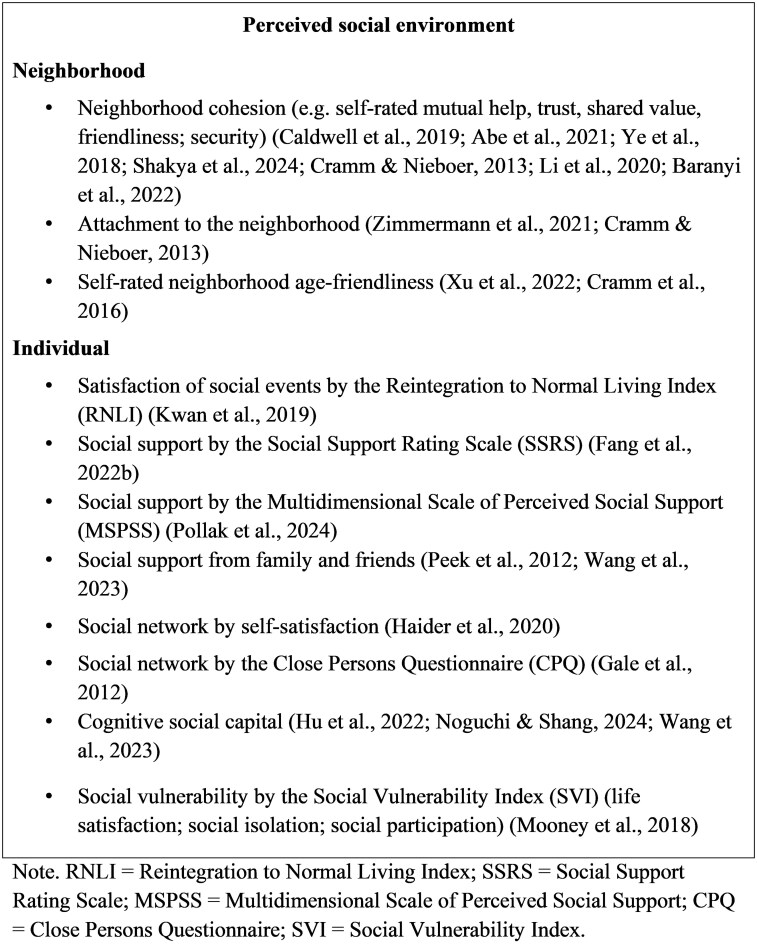
Perceived social environment measures in selected studies.

**Figure 4:**
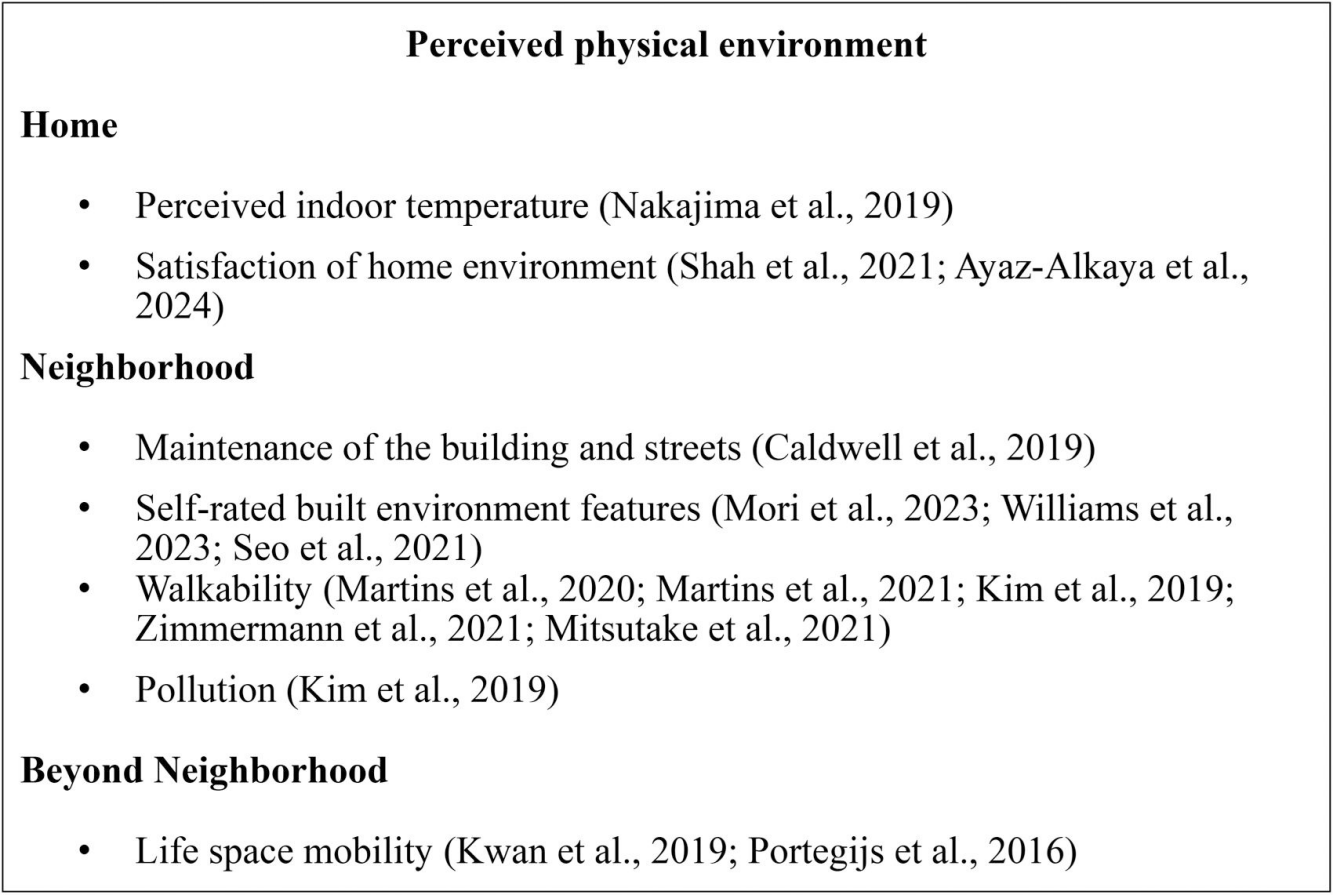
Perceived physical environment measures in selected studies.

**Figure 5:**
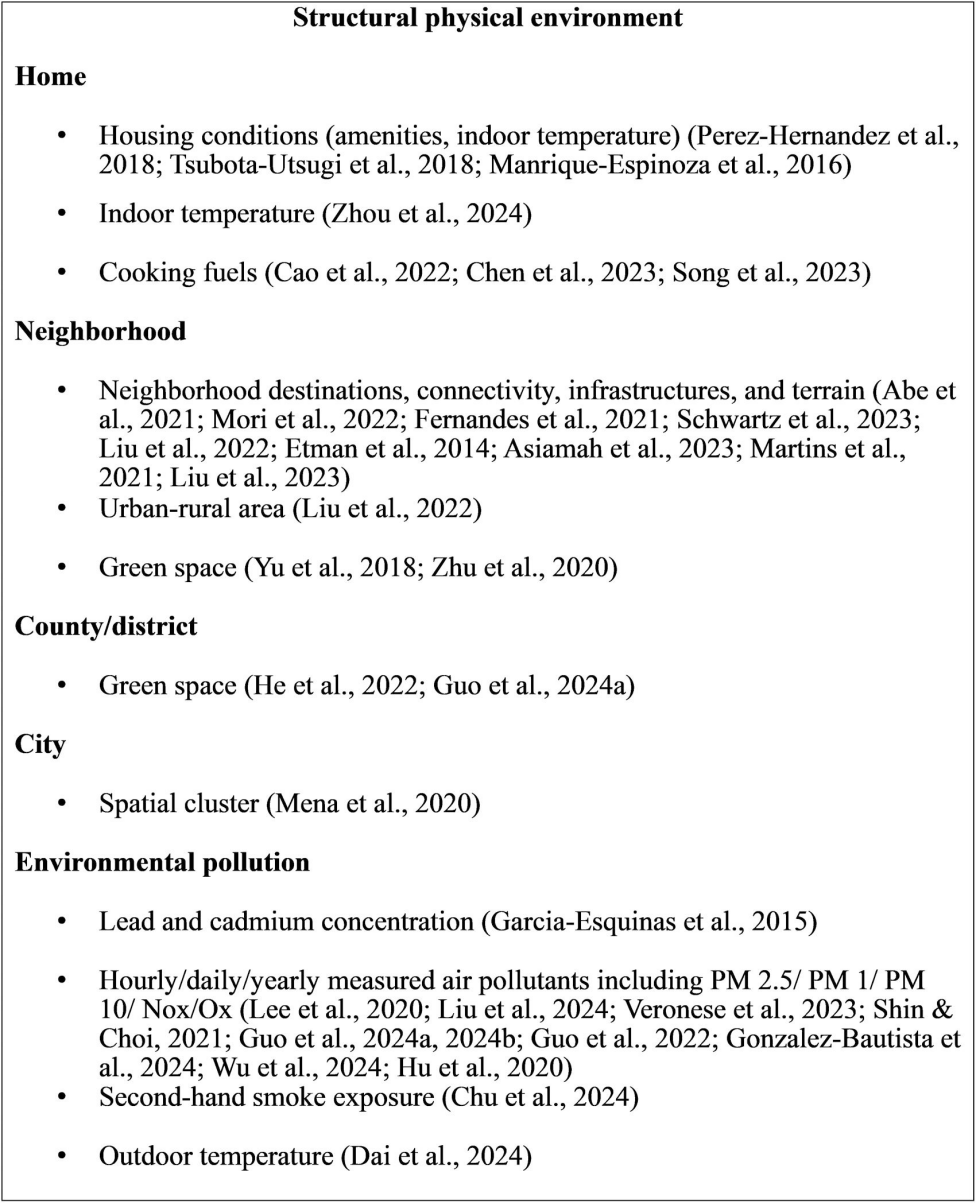
Structural physical environment measures in selected studies.

**Figure 6:**
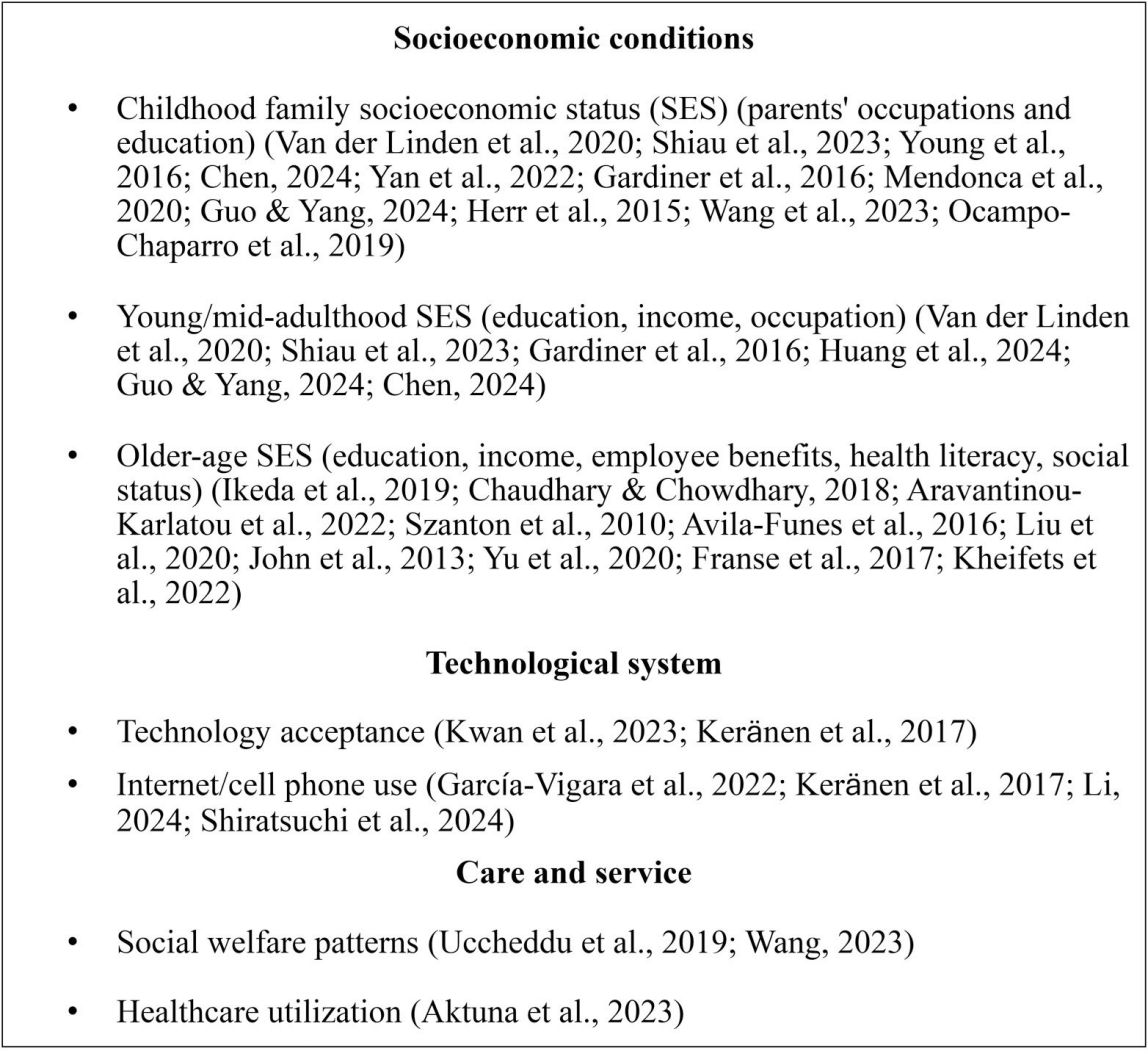
Socioeconomic conditions, technological systems, and care/service factors in selected studies.

According to the CODA framework, environmental factors are classified into five domains: social, physical, socioeconomic, care and service, and technological. Notably, we revised the operationalization of the socioeconomic domain. Whereas Wahl and Gerstorf (2018, 2020) conceptualized socioeconomic characteristics primarily at the neighborhood and state levels, this scoping review reassigned these indicators to the physical and social domains. In contrast, the new socioeconomic domain emphasizes individual-level socioeconomic status from a life-course perspective. Figures 2–6 present these revised categories and their corresponding measures. Detailed definitions, key findings, and study summaries are included in the Supplementary Materials.

#### Social environment

We categorized the social environment into structural and perceived dimensions. The structural dimension captures the tangible, quantifiable features of social relationships and neighborhood settings that influence frailty, and is distinct from subjective perceptions or feelings about the social environment. This disparity between structural and perceived measures is supported by the residential satisfaction paradox, which suggests that individuals’ subjective evaluations of their social environment may not align with objective structural indicators.

Figure 2 presents the types of structural social environments examined in the selected studies. At the individual level, disengagement from social activities, measured by the Lubben Social Network Scale (LSNS), is generally associated with increasing frailty (e.g., Chen et al., 2015), although one study reported inconsistent results (Ge et al., 2022). Social connection (e.g., frequency and number of family or friend contacts) and social participation (e.g., number and frequency of social activities) are generally linked to frailty risk (e.g., Hsu & Chang, 2015). At the neighborhood level, indicators of deprivation and sociodemographic composition (e.g., proportions of migrant or older residents) tend to be associated with higher frailty risk. Conversely, social group characteristics (e.g., number and participation rate) and neighborhood social capital are associated with slower frailty progression.

Figure 3 presents the types of perceived social environments examined in the selected studies. At the individual level, perceived social environment measures include satisfaction with social events, social support, cognitive social capital, and social vulnerability; these constructs reflect an individual’s experience of being respected, supported, and understood in society. At the neighborhood level, perceived social environment measures include neighborhood cohesion (e.g., self‑rated mutual help, trust, shared values, friendliness, and security) and attachment to the neighborhood, both of which are associated with lower odds of frailty.

#### Physical environment

Similar to the social environment, we categorized the physical environment into structural and perceived dimensions. The structural physical environment includes objective, measurable features, while the perceived physical environment reflects individuals’ subjective experiences and satisfaction. Our results describe the physical environment at three levels: the home environment, including living conditions and indoor temperature; the neighborhood environment, characterized by walkability, access to amenities, and neighborhood maintenance; and environments beyond the neighborhood, focusing on county‑ and city‑level green spaces.

Figure 4 presents the types of perceived physical environments examined in the selected studies. Shah et al. (2021) and Ayaz-Alkaya et al. (2024) found that older adults satisfied with their living environment had lower frailty scores. Nakajima et al. (2019) linked perceived cold indoor temperatures and dissatisfaction with economic status to higher frailty risk, with socioeconomic status moderating the temperature–frailty relationship.

At the perceived neighborhood level, neighborhood walkability, measured by scales such as the Neighborhood Environment Walkability Scale (NEWS), is often inversely associated with frailty; poor walkability, fewer destinations, poor street connectivity, inadequate facilities, poor esthetics, and high crime are linked to higher risk. However, Zimmermann et al. (2021) found no association between self-reported walkability satisfaction and frailty. Lower satisfaction with neighborhood maintenance and unmet transportation needs also predict greater frailty (Caldwell et al., 2019). Access to parks, fresh food stores, sidewalks, and appealing views reduce frailty risk, with walking, depressive symptoms, and social support mediating this relationship. Life-space mobility is negatively associated with frailty, with reduced life-space linked to higher frailty and pre-frailty.

Figure 5 presents the types of structural physical environments. In the home environment, the review included studies focused on cooking fuels, indoor temperature, home amenities, and housing types. Cross‑sectional and longitudinal studies found that the use of polluting cooking fuels (e.g., kerosene, coal, firewood) increases frailty risk, whereas clean fuels were protective (e.g., Cao et al., 2022). High indoor summer temperatures and poor home amenities are also linked to greater frailty. Tsubota-Utsugi et al. (2018) reported that among survivors of the 2011 East Japan earthquake, psychological distress and poor social networks were associated with frailty in men who experienced extensive housing damage or lived in temporary housing.

Neighborhood definitions varied, but key findings were consistent: living in areas with fewer amenities was associated with higher frailty, whereas greater land‑use mix, pedestrian connectivity, and access to parks, green spaces, and fresh food stores were associated with lower risk (e.g., Martins et al., 2021). Schwartz et al. (2023) reported higher odds of pre‑frailty or frailty in “resource‑poor” neighborhoods, defined by limited access to exercise opportunities and healthy food, lower socioeconomic status, and rural versus urban location. Mori et al. (2022) found that, over three years, access to parks, appealing views, fresh food stores, and welcoming facilities was associated with reduced frailty risk. Asiamah et al. (2023) reported that sedentary behavior was associated with frailty in both flat and hilly neighborhoods, with a stronger association in hilly areas. Fernandes et al. (2021) found that riverside living was associated with decreased frailty. Liu et al. (2023) measured community infrastructure (sewerage, waste management, paved roads, toilets, electricity) and found that better infrastructure had a protective effect on frailty over seven years and moderated the impact of life‑course socioeconomic deprivation. Liu et al. (2022) found that better infrastructure and exercise facilities slowed the increase in frailty among rural, but not urban, residents.

Beyond the neighborhood, higher county‑level green space was associated with lower frailty, whereas air pollution, such as PM1, PM2.5, PM10, and O3, was linked to increased frailty risk, with stronger effects observed in men, adults over 75 years, and rural residents. Studies have also shown that air pollutants mediate the relationship between greenness and frailty. Additional risks included secondhand smoke exposure and elevated blood lead levels, both of which were linked to greater frailty.

#### Socioeconomic conditions

As shown in Figure 6, socioeconomic conditions span three stages: childhood, young to mid-adulthood, and older age. Childhood socioeconomic status (SES), such as parents’ occupations and education, influenced both baseline frailty and its progression. Adult SES (education, income, occupation) mediated the effects of childhood conditions on frailty, while lifelong socioeconomic advantages provided protection against it (e.g., Chen, 2024). Low SES at any life stage was associated with higher frailty risk, although upward mobility might have offset early disadvantages. In older age, higher education and income reduced frailty risk and supported recovery from pre-frailty, whereas low income, poor health literacy, and limited social benefits were associated with higher frailty.

#### Care/service and technological systems

As shown in Figure 6, care and service are assessed at state and individual levels. At the state level, Uccheddu et al. (2019) found that gender and socioeconomic inequalities in frailty were weaker in Northern European welfare models (e.g., Sweden, Denmark), which are characterized by high defamilization and decommodification. Similarly, Wang (2023) reported that childhood disadvantages such as parental absence and poor parent–child relationships increased frailty risk, with variations across welfare regimes. At the individual level, Aktuna et al. (2023) linked frequent healthcare use, such as emergency visits, hospital admissions, and medication use, with higher frailty risk.

Regarding technology, studies examined Information and Communication Technology (ICT) use and older adults’ perceptions. Shiratsuchi et al. (2024) found no association between ICT usage frequency and frailty after adjustment. Conversely, Garcia-Vigara et al. (2022) reported that ICT use reduced frailty risk among middle‑aged and older women. Keranen et al. (2017) observed that frail noninternet users held more negative views on technology than their nonfrail peers. During the COVID-19 pandemic, technology acceptance moderated the link between physical activity and frailty (Kwan et al., 2023).

### A synthesized framework based on CODA

#### Key components

**Figure 7:**
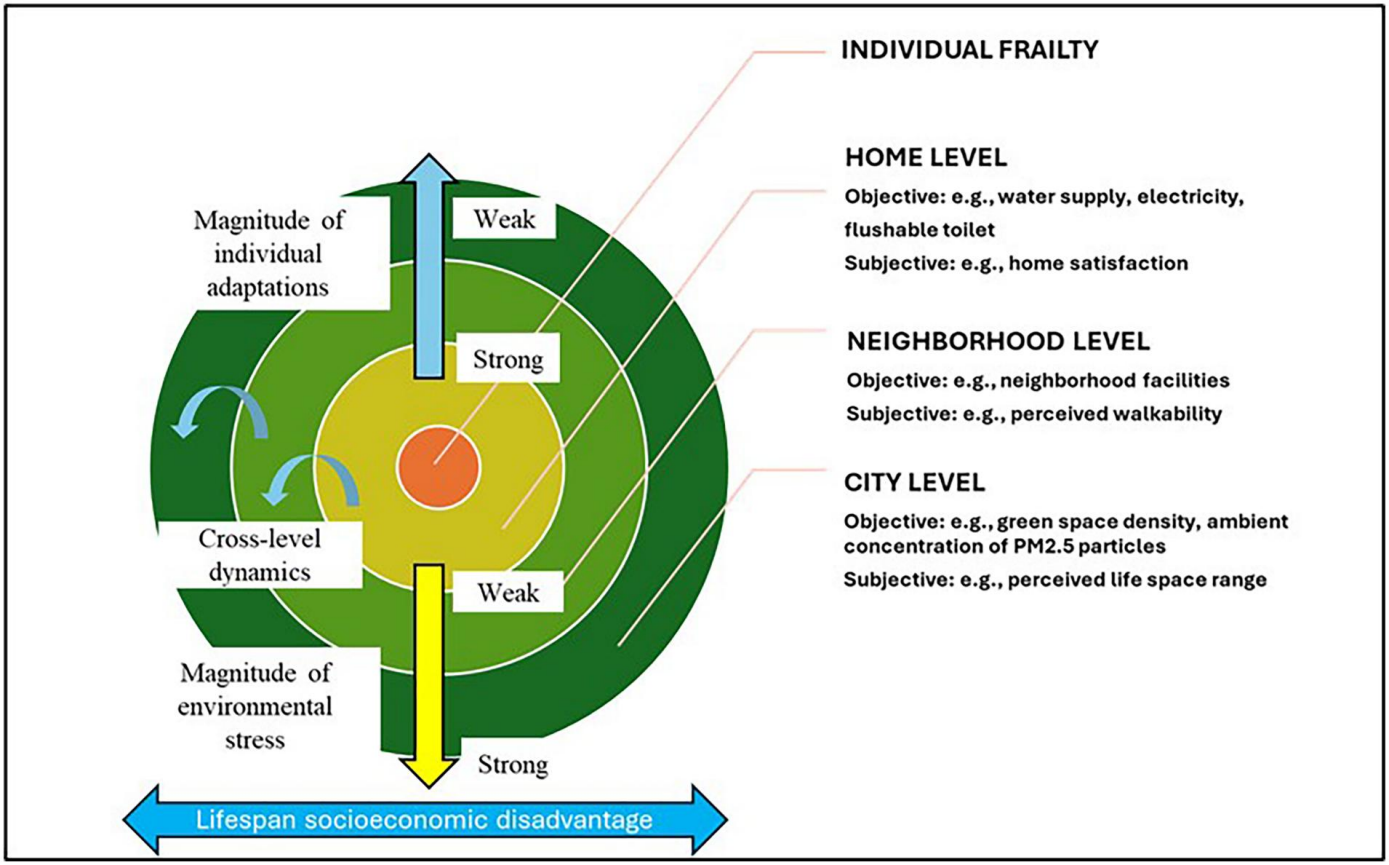
The synthesized theoretical model.

As shown in Figure 7, this scoping review introduces an integrative model of P–E interactions in frailty, organized hierarchically across three spatial levels: home, neighborhood, and city (or broader environments). In this model, frailty serves as the central outcome of ongoing P–E interactions and is shaped by the dynamic interplay between environmental stressors, such as physical deterioration and social disorganization, and individual adaptive responses, both behavioral and psychosocial. In the context of frailty, we argue that P–E interaction itself constitutes stress, understood as the adaptive challenges individuals face in response to contextual demands (Wahl & Gerstorf, 2018). From this perspective, the P–E stress process is not merely one element but a fundamental process underlying frailty. It reflects what Lawton and Simon (1968) describe as adaptive tension and environmental docility across environmental domains.

**Figure 8:**
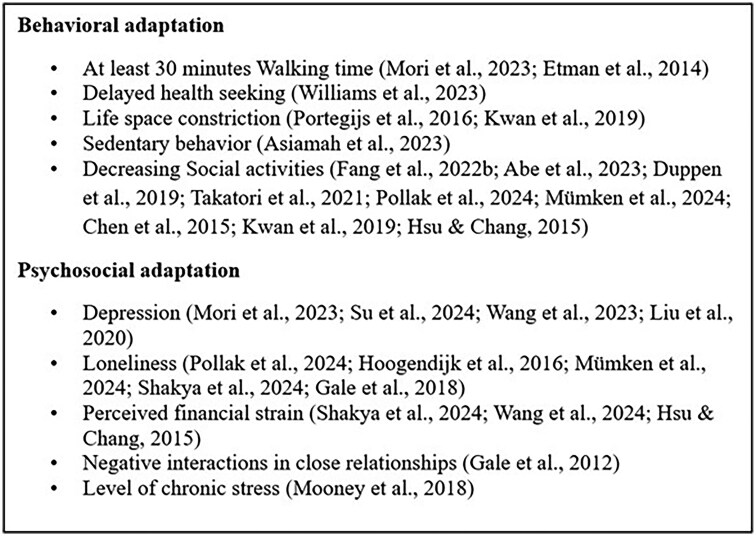
Behavioral and psychosocial adaptation measures in selected studies.

At each spatial level, frailty risk is influenced by two major types of environmental stress: physical disorder (e.g., deterioration of home infrastructure and neighborhood facilities) and social disorganization (e.g., reduced cohesion and weaker social support networks). Empirical evidence links increased frailty risk to both types of stress. These domains can be measured at each spatial level both objectively (e.g., green-space density, number of amenities) and subjectively (e.g., satisfaction with home, perceived walkability). In particular, objectively measured factors such as pollutant exposure contribute to frailty through physiological pathways, including oxidative stress, inflammation, and epigenetic modifications, all of which are implicated in frailty pathogenesis. Subjective environmental experience involves a process of transforming space into place, often described as place integration, where individuals form emotional connections with their environment (Bigonnesse & Chaudhury, 2022). Adaptation to environmental stress occurs at each level and can be classified as either behavioral or psychosocial. As depicted in Figure 8, behavioral adaptations include reduced physical activity, delayed healthcare seeking, and increased sedentary behavior. Psychosocial adaptations include emotional and social consequences, such as depression, loneliness, and relationship strain.

The model also incorporates the cumulative influence of life-course socioeconomic conditions and the supportive roles of technology, services, and care. Lifespan socioeconomic factors shape both frailty and perceptions of the environment through life‑course processes, in which specific experiences or exposures at different stages of life exert strong and lasting effects on later‑life health (Baranyi et al., 2022; Herr et al., 2015; Liu et al., 2023). Technological and care resources, in turn, act as buffers against environmental stress (Andrew & Keefe, 2014; Bigonnesse & Chaudhury, 2022). Negative socioeconomic circumstances are linked to less favorable perceptions of the environment, whereas technological and care services can provide compensatory support as frailty progresses.

#### Cross-level dynamics

Building on the integrative framework discussed above, the model highlights two fundamental cross‑level dynamics. First, the “hierarchical stair” principle describes a progression: older adults’ ability to engage with larger, more complex environments depends on their capacity for proactive adaptation to stressors in the proximal context, such as the home and its direct surroundings. Second, the model proposes a spatial-differential pattern in adaptation competence and environmental stress. Individual adaptive competence, namely the ability to actively modify or buffer the environment, is greatest at the home level and declines as the spatial context expands to the neighborhood and beyond. This reduced competence means that, as environments become more distal, older adults often shift from proactive adaptation to a more passive or “docile” response. These larger‑scale contexts present challenges that are increasingly difficult to control or change. As a result, environmental stress originating from distal levels (e.g., neighborhood disorder, city‑ or societal‑level disadvantage) is less easily mitigated by individual adaptation, whereas stress encountered within the home can more often be managed directly through behavioral and psychosocial adaptations.

At the most proximal level, home environments are where individual competence can be expressed most fully, because personal control and emotional attachment are greatest. These conditions facilitate effective behavioral and psychosocial coping. Higher satisfaction with the home environment, for example, with overall living conditions, and better access to residential facilities are associated with lower frailty risk (Chen et al., 2023; Chu et al., 2024; Shah et al., 2021). However, consistent with P–E fit theory, when broader physical or social environmental stresses exceed adaptive capacity, frailty may intensify.

At the neighborhood and city levels, environmental stress increases frailty risk by limiting opportunities for physical activity, social interaction, and access to services and support. Individual adaptation at the neighborhood level is moderate, as older adults have some influence but less direct control than at home. Neighborhood environments affect home‑level functioning by shaping daily routines, mobility, and outdoor social participation. Attachment to the neighborhood and strong community support foster social activities and promote healthier behaviors. At the city level, individual adaptation is weakest, as greater distance and scale reduce personal capacity to influence environmental conditions. City-level stressors can constrain neighborhoods and homes by limiting outdoor activity and social engagement. Conversely, well-designed city policies and infrastructure can improve neighborhood quality and support home adaptation by enhancing access to services and material resources.

## Discussion

This review offers one of the first systematic overview of research on person–environment (P–E) interactions in frailty. It underscores the significant role of the environment and the need to consider its heterogeneous components. To address this complexity, the review proposes a refined model that integrates the CODA framework with existing empirical findings, providing a more comprehensive perspective on its application to frailty.

This scoping review identifies the physical frailty model as the most frequently used frailty concept. One possible reason is that frailty has long been studied in biomedical and clinical fields, which emphasize physical and biological markers consistent with this model. Additionally, some studies prioritize binary frailty classifications (frail vs nonfrail), which correspond more closely to the physical frailty model. However, this unequal focus has important conceptual implications. Focusing primarily on the physical dimension risks overlooking frailty’s complex interactions with cognitive and psychological factors, resulting in an incomplete understanding of its multifactorial nature. Gale et al. (2018) illustrated this complexity using two frailty measures, one based on the deficit accumulation model and the other on the physical frailty model, to examine loneliness and structural social isolation. They found that high loneliness, but not social isolation, increased the risk of developing frailty phenotypes, whereas neither factor was associated with changes in the frailty index.

Beyond the dominance of the physical frailty model, this review also reveals substantial variability in how the frailty phenotype and frailty indices are operationalized across studies. Consistent with Theou et al. (2014), we observed that many studies modify the frailty phenotype criteria. Fritz et al. (2020) cautioned that such variations lack systematic validation, potentially undermining the phenotype’s validity. Regarding frailty indices, we found considerable variation in the number of deficits included and cutoff points used across studies, often without adequate justification. This inconsistency complicates comparisons and may affect measurement reliability. Notably, a narrative review by Dent et al. (2016) identified developments in frailty measurement, including electronic medical records (EMR), artificial intelligence, and epigenetics. However, our review found no included studies that incorporated these features. As frailty assessment increasingly adopts new technologies, more empirical research is needed to understand their implications for P–E interactions.

### CODA framework and advances

The CODA framework provides a foundational structure for P–E research by defining the environment across five key domains and emphasizing the processes of agency, belonging, and stress in P–E interactions. Despite these strengths, implementation of the CODA framework has been impeded by a lack of advanced environmental measurement tools and comprehensive indicator pools across all levels. In addition, there has been limited exploration of simultaneous or interactive effects of multiple domains and little illustration of cross‑level buffering or exacerbation effects (Wahl & Gerstorf, 2020). Responding to these gaps, our scoping review systematically collects existing measures and proposes a refined, spatial‑differential model for understanding P–E interactions in frailty.

#### Environmental measures

This scoping review makes two key advances. First, we comprehensively map environmental measures employed in frailty research, expanding the available indicator pool. We also clarify how these measures are operationalized across micro (home), meso (neighborhood), and macro (city and beyond) scales. This mapping can lay the foundation for more environmentally sensitive frailty research.

Following Wahl and Gerstorf’s (2020) call to jointly consider environmental domains in aging research, we demonstrate the interactive roles of socioeconomic status (SES), technological systems, and care and services in frailty, rather than examining these factors in parallel. This integrative approach aligns with life-course theories, particularly critical period, accumulation, and sensitive period perspectives, which emphasize that early‑ and mid‑life socioeconomic disadvantages shape health in later life. For example, Herr et al. (2015) underscored that education shapes knowledge and skills, which in turn influence attitudes, behaviors, and occupational opportunities. As a result, higher educational attainment reduces exposure to physical and psychosocial hazards throughout life, thereby lowering the risk of frailty in older age. Similarly, access to information and communication technology (ICT) and health and social care services can also be understood within the life-course perspectives. Individuals with higher SES face fewer barriers to technology and social support (Hulur & Macdonald, 2020), and these advantages help buffer external stressors, including pandemics, natural disasters, and societal inequalities (Kwan et al., 2023; Tsubota-Utsugi et al., 2018; Uccheddu et al., 2019). Despite these insights, research specifically examining these interactive pathways remains limited, underscoring the need for further investigation.

Second, we argue that the distinction between subjective and objective environmental measures is not simply a methodological choice but is fundamental to understanding P–E dynamics. Whereas the original CODA framework treats objective and subjective measures as complementary sources for describing environmental context (Wahl & Gerstorf, 2018), we propose that the alignment or divergence between individual perceptions and structural realities plays a central role in adaptation and well-being in later life (Rowles & Cutchin, 2025). The quest for “being in place” is not just about what environments physically exist or how settings are externally rated, but about how individuals seek a sense of belonging, comfort, and equilibrium. This sense of being in place may either align with or diverge from objectively assessed environmental qualities.

Building on this conceptual distinction, our review illustrates how subjective and objective environmental measures operate in practice. Objective environmental factors, such as air pollution measured by PM2.5 and PM10 concentrations, affect frailty through biological mechanisms, demonstrating a direct physical impact on the body. In contrast, subjective environmental measures capture how individuals actively connect with and perceive their surroundings (Bigonnesse & Chaudhury, 2022). In addition to the well‑known paradox of home satisfaction (Wahl & Oswald, 2010), Martins et al. (2021) found only a weak positive correlation between perceived neighborhood walkability and objective land‑use mix. This suggests that older adults’ perceptions often differ from measurable environmental features. As people age, their relationship with place requires greater efforts at “place integration,” the continuous process of reconciling personal changes (mobility, health, social loss) with changing environments (Rowles & Cutchin, 2025). This challenges future studies to consider both the physical affordances of place and its psychological and social dimensions. However, some instruments, such as the Social Support Rating Scale (Noguchi & Shang, 2024), the Chinese Shortened Social Capital Scale (Hu et al., 2022), and a social vulnerability index (Abeliansky et al., 2021), combine subjective and objective elements, complicating efforts to separate their distinct contributions.

Despite the recognized importance of the environment in influencing frailty among older adults, there is a lack of research focusing on home and beyond‑neighborhood environments. Most of the included studies have concentrated on neighborhood‑level features, such as walkability, access to amenities, and social cohesion, while neglecting the other two scales. This gap is especially concerning given that older adults spend substantial time within their homes and immediate surroundings (Oswald & Wahl, 2005). Older adults often develop strong cognitive and emotional ties to their living spaces (Wahl & Oswald, 2010). For many, the home is not merely a physical space, but a place imbued with a sense of belonging and identity (Wahl & Gerstorf, 2020). This suggests the need for more subjective measures that reflect how older adults maintain balance and well-being in their homes. The COVID-19 pandemic has further highlighted this need, as prolonged home confinement has been associated with various adverse health outcomes (Rodriguez-Fernandez et al., 2021). Strengthening the understanding of P–E interactions at the home scale is, therefore, critical, particularly to distinguish how these interactions differ from those occurring at larger spatial scales. Similarly, broader built and natural environments, such as city-level infrastructure, regional green spaces, and transportation networks, remain underexplored despite their potential to shape mobility, access to care, and social participation among older adults.

#### Cross-level dynamics

The CODA framework explicitly distinguishes between proximal contexts, characterized by direct interaction with people and immediate environments, and distal contexts, which involve environments or structures with which people interact in more indirect ways (Wahl & Gerstorf, 2020). Nevertheless, there remains a need for more precise operationalization, empirical testing, and quantification of these P–E interactions. Important questions are still unresolved, including whether more distal contexts (e.g., wider community or societal structures) buffer or exacerbate the effects of proximal contexts (e.g., home environment or close relationships). A critical review by Wahl and Gerstorf (2020) on life space, docility, and proactivity highlights that changes in life space are not merely passive consequences of aging. Instead, they can represent active adaptations, in which individuals strategically manage their environmental exposure by increasing the “density of control.” Building on these theoretical insights, our review helps integrate life‑space (Cantor, 1975) and P–E fit (Lawton & Simon, 1968) theories with the CODA framework.

First, our scoping review offers a spatial‑differential perspective. In the CODA framework, Wahl and Gerstorf (2020) advanced an age‑differential perspective on the duality of docility and proactivity, showing that in the Third Age, proactive adaptation tends to dominate as older adults preserve resources and extend life space. In the Fourth Age, by contrast, increasing vulnerability leads to greater environmental docility. We argue that the balance of docility and proactivity is shaped not only by age but also by levels of spatial context. Individuals are typically more empowered to adapt proactively in their immediate environments, while they become more docile and dependent as the context becomes more distal and constraints increase.

Within this spatial‑differential perspective, we argue that home‑level adaptation is strongest because the home is not merely a spatial container but a rich interplay of social, material, and symbolic meanings that support well‑being and resilience in later life (Wiles & Coleman, 2024). This strength arises from several key factors: the extended time older adults spend at home (Oswald & Wahl, 2005), deep cognitive and emotional ties to their living environment (Wahl & Oswald, 2010), and a heightened sense of belonging and identity (Wahl & Gerstorf, 2020). Critically, home serves as a primary setting for renegotiating one’s relationship with the environment through ongoing creative strategies and adaptations to changing needs (Wiles & Coleman, 2024). The home environment can be personalized and modified to match evolving functional abilities, helping maintain independence and safety while promoting daily self-care (Zimmermann et al., 2021). Furthermore, proximity to family and established social networks is typically greatest at home, providing practical support and facilitating adaptive responses (Thinuan et al., 2020). These unique capacities to negotiate, transform, and draw meaning from one’s living environment continually support the process of adaptation at home.

Meanwhile, larger scale environments, such as neighborhoods, towns, or wider regions, are often considered distal to older adults. Finlay et al. (2024) define public and commercial places outside the home and workplace as “third places.” They argue that age‑related declines in mobility, social exclusion, and rising health needs make access to, and engagement with, these environments increasingly challenging. Similarly, life‑space constriction theory (Xue et al., 2007) posits that shrinking life space, manifested by reduced range and frequency of movement, is associated with the development of frailty. The standard Life-Space Assessment operationalizes life space in five ascending levels, from bedroom, home, and yard to the neighborhood and beyond (Baker et al., 2003). In Xue et al. (2007), “life space not constricted” is defined as leaving one’s neighborhood four or more times weekly. Notably, these tools consistently assign greater weight to more immediate, proximal spaces, reflecting their primary relevance for daily functioning in later life.

Second, the “hierarchical stair” principle posits that active engagement with broader, more distal environments depends on first adapting and meeting foundational needs at more proximal levels, such as the home. Spatially, within the home–neighborhood–city hierarchy, successful P–E adaptation at home is essential before meaningful engagement at the neighborhood level is possible. Engagement at wider scales requires foundational fit and support from prior levels. For example, Thinuan et al. (2020) observed that, as older adults experience declining physical strength, areas beyond the immediate home environment become increasingly challenging. In response, most participants adapted by establishing new routines and focusing on household chores that helped them maintain physical activity and contribute to family life. Similarly, Duppen et al. (2019) found that, as frailty progresses, older adults tend to withdraw from hobbies such as fishing or cycling and rediscover value in activities offered by their local community. These patterns illustrate how proximal adaptation forms the foundation for maintaining participation as one’s life space evolves.

Viewed from an individual standpoint, the “hierarchical stair” principle highlights that personal resources built at proximal levels are prerequisites for broader participation. When adaptation at these foundational levels is undermined, efforts and adaptive energy are redirected inward, making wider engagement less feasible or less safe. From this perspective, changes in life space are not inherently negative. Maintaining agency and meaningful engagement within a smaller, well‑supported space often sustains well‑being, challenging the view that only expansion is positive (Wahl & Gerstorf, 2020).

Despite these conceptual propositions about the “hierarchical stair” principle, there is a clear lack of qualitative and quantitative evidence substantiating the model’s cross‑level dynamics. This scoping review reveals a shortage of theories conceptualizing P–E interactions at these levels. Most existing theories are rooted in the neighborhood level, such as social disorganization theory (Baranyi et al., 2022), life-space constriction (Xue et al., 2007), and neighborhood stress response (Li et al., 2020). These approaches have limitations when applied to smaller scales such as the home environment. As the home holds special significance for older adults, loss of the ability to live at home often results in a move to an assisted living facility. We argue that constriction at the home level may have different implications from those at the neighborhood level. These neighborhood‑focused theories may fail to capture the “back‑and‑forth” or bidirectional interactions that older adults experience within their home environments. Theories at the home level should illustrate how older adults engage with, adapt to, and derive meaning from their environments in the context of frailty. At larger scales beyond the neighborhood, conceptual models are even scarcer, with few exploring how city, regional, or national structures influence health or account for varied effects by SES, age, gender, or health status. Investigating these cross-level relationships could yield critical insights for advancing P–E theory across the aging spectrum.

Taken together, these cross‑level gaps reflect not only empirical limitations but also the ways P–E interactions have been theorized. At a more foundational level, the refined framework relies on a small cluster of conceptual work (e.g., CODA and related P–E models). As a result, our synthesis is grounded in a relatively narrow theoretical lineage and should be interpreted with caution. These frameworks explicitly prioritize environmental docility/proactivity and life space as key mechanisms, and they emphasize health as the central outcome of contextual aging. Consequently, our model may underrepresent alternative mechanisms, such as Gerotranscendence and Socioemotional Selectivity Theory. Gerotranscendence describes a developmental shift from a materialistic outlook to a more cosmic and transcendent perspective, whereas Socioemotional Selectivity Theory links perceived time horizons to emotionally meaningful goal selection. By prioritizing health as the primary outcome of P–E interactions, these frameworks may also overlook dimensions such as identity, citizenship, and rights, which are increasingly emphasized in gerontology. Moreover, CODA has been developed by closely connected author groups who frequently elaborate their own earlier formulations, leading to a concentration of authorship and theoretical development. This concentration may introduce circularity in how P–E interactions and “good” environments (e.g., age‑friendly, resource‑rich, supportive neighborhoods) are conceptualized. For instance, the “barrio advantage” literature shows that living in low‑income, ethnically dense Mexican American neighborhoods can confer protective effects on health and frailty despite limited material resources (Aranda et al., 2011). This evidence challenges a narrow emphasis on resource‑rich settings and underscores the need for independent empirical testing and systematic comparison with alternative theoretical perspectives.

Beyond these gaps in theory and evidence, this review identifies another limitation: the limited use of subgroup analyses, which restricts understanding of nuanced population differences in P–E interactions. Gender is the primary subgroup indicator reported. Selected studies highlight gender differences across socioeconomic conditions, environmental exposures, and social networks. Many studies found that poorer functional health among older women largely stems from socioeconomic factors such as education, neighborhood deprivation, and income (e.g., Szanton et al., 2010). Chen et al. (2023) noted that indoor cooking fuel pollution disproportionately affects women, especially those who are illiterate or economically disadvantaged. Woo et al. (2005), Gale et al. (2012), and Haider et al. (2020) found that negative social interactions, including limited family contact and lack of community engagement, increase frailty risk more in women than in men. These findings provide important insights into gender differences in P–E interactions related to frailty. However, these differences are underrepresented in the synthesized model. For example, it remains unclear how gender influences P–E interactions at home and at larger scales. How do social roles affect the attachment men and women develop to their homes? Are these differences amplified at the city level? Notably, in the context of climate change, Tsubota-Utsugi et al. (2018) found that women living in temporary housing after disasters faced an increased risk of frailty due to limited access to facilities and disrupted community ties. Beyond gender disparities, there is a general lack of subgroup analyses in frailty research. Future studies should prioritize this area to deepen understanding of how frailty develops across diverse populations. Addressing these subgroup and cross‑level gaps requires improved measurement and research designs.

To empirically test the model and address the gaps identified in this review, it is crucial to follow Wahl and Gerstorf’s (2020) call for advancements in measurement and study design. Measurement should prioritize capturing dynamic rather than static processes. At the home level, sensor-based monitoring and real-time behavioral detection can reveal interaction patterns (Alberdi Aramendi et al., 2018; Baniassadi et al., 2024). Interviewer‑based assessments can complement these methods by providing objective, less biased appraisals (Hoogesteyn et al., 2020; Riley, 2018). For larger environments, integrating city‑level data, such as satellite imagery, can identify neighborhood vulnerabilities and track urban change (Finlay et al., 2015; Phillipson et al., 2024). Analyses should model multiple domains simultaneously and over time, using multilevel and longitudinal designs. Interdisciplinary collaboration with urban planning, gerontechnology, public health, and social sciences is vital to fully address the complexity of these dynamics.

## Limitations

Several gaps affect the generalizability and comprehensiveness of this scoping review. First, several included studies draw on the same or closely related Chinese longitudinal data sources and focus on exposure to air pollutants. This clustering in data source, country, and topic means that some apparent replication of air‑pollution–frailty associations may partly reflect repeated analyses of similar cohorts and policy contexts rather than fully independent evidence. In particular, China’s implementation of the Clean Air Action in 2013 to reduce air pollution may shape exposure patterns and health trajectories in ways that do not directly translate to other countries. In contrast to this concentration of air‑pollution evidence in China, there is a notable scarcity of studies from South America and Africa. Sánchez-González and Rodríguez-Rodríguez (2016) highlighted that environmental gerontology in Mediterranean Europe emphasizes property and public amenities, while Latin American research focuses on poverty, social exclusion, and access to resources. This geographic gap limits applicability across diverse cultural and health system contexts and risks overlooking region-specific factors. Second, there is a lack of studies on technology and care services. While the focus on observational studies may have excluded some intervention research, this gap could also reflect limited adoption of these innovations in practice. Third, qualitative research is underrepresented in this review. Many qualitative studies were excluded due to the absence of standardized frailty measures, often using “frailty” as a broad label rather than a defined geriatric syndrome. Given that the CODA framework relates closely to theories of psychological attachment and control beliefs, areas well suited to qualitative approaches, this represents a significant limitation. Finally, this review is predominantly descriptive. Most included studies examine frailty outcomes rather than the processes underlying its development, which constrains understanding of how frailty evolves in relation to environmental factors.

We argue that some of these limitations may be compounded by the exclusion of nonpeer‑reviewed and non‑English studies in the search process. While this decision enhances methodological rigor and practical feasibility, it narrows the scope of included evidence and may introduce language and publication bias. Crucially, the available studies are largely associative and rarely test cross‑level mechanisms. As a result, the “hierarchical stair” and spatial‑differential adaptation concepts proposed in this study should be regarded as heuristic, largely hypothetical constructs rather than empirically validated conclusions. The model, therefore, serves primarily as a synthesis and a framework for future research, and the current data do not offer robust validation of its cross‑level dynamics. Future studies using independent cohorts, diverse policy and cultural settings, and alternative theoretical frameworks will be essential to test, refine, or challenge the propositions advanced in this study.

## Conclusion

In summary, this scoping review highlights the critical role of person–environment interactions in understanding frailty across spatial levels. Building on this perspective, our review emphasizes that the CODA framework, through its multiple environmental dimensions and interaction processes, shapes how frailty develops. While CODA offers a useful foundation, gaps in research on home and wider environmental contexts may lead to an incomplete understanding of factors influencing frailty. Given the scarcity of studies on interaction processes and spatial dynamics, the proposed model should be viewed as a heuristic framework for developing and testing new hypotheses. Therefore, future studies should prioritize empirical testing of this multilevel approach. Future research should also strengthen measurement methods, enhance study designs, and foster interdisciplinary collaboration.

## Supplementary Material

gnag044_Supplementary_Data

## Data Availability

Data, analytic methods, and materials are available to other researchers for replication purposes. They can be requested from the corresponding author. A scoping review protocol was published in the Open Science Framework (https://doi.org/10.17605/OSF.IO/4P83E) on December 3, 2024.
