# Supplementary material for: Person–environment interaction processes in frailty: a scoping review
Source: Gerontologist. 2026 Apr 6;66(6):gnag044. doi: 10.1093/geront/gnag044 (PMC13180652; doi:10.1093/geront/gnag044)
Supplement: gnag044_Supplementary_Data [file gnag044_supplementary_data.pdf]

Person–Environment Interaction Processes in Frailty: A Scoping Review

Junjie Zhang, PhD, Xiayu Wang, PhD, Paul S.F. Yip, PhD

Supplementary materials

Supplementary Table 1 Preferred Reporting Items for Systematic Reviews and Meta-Analyses Extension for Scoping Reviews (PRISMA-ScR) Checklist

Supplementary Table 2 Detailed information on eligibility criteria

Supplementary Table 3 Detailed information on search terms

Supplementary Table 4 Detailed information on extracted information

Supplementary Table 5 Study title, author, design, and sample characteristics

Supplementary Table 6 Frailty measurements

Supplementary Table 7 Person-Environment interaction findings and summaries

References (all)

Supplementary Table 1 Preferred Reporting Items for Systematic Reviews and Meta-Analyses Extension for Scoping Reviews (PRISMA-ScR) Checklist

Preferred Reporting Items for Systematic reviews and Meta-Analyses extension for Scoping Reviews (PRISMA-ScR) Checklist[1]

SECTION	ITEM	PRISMA-ScR CHECKLIST ITEM	Location
TITLE			
Title	1	Identify the report as a scoping review.	Title section
ABSTRACT			
Structured summary	2	Provide a structured summary that includes (as applicable): background, objectives, eligibility criteria, sources of evidence, charting methods, results, and conclusions that relate to the review questions and objectives.	Introduction
INTRODUCTION			
Rationale	3	Describe the rationale for the review in the context of what is already known. Explain why the review questions/objectives lend themselves to a scoping review approach.	Introduction
Objectives	4	Provide an explicit statement of the questions and objectives being addressed with reference to their key elements (e.g., population or participants, concepts, and context) or other relevant key elements used to conceptualize the review questions and/or objectives.	Introduction
METHODS			
Protocol and registration	5	Indicate whether a review protocol exists; state if and where it can be accessed (e.g., a Web address); and if available, provide registration information, including the registration number.	Introduction
Eligibility criteria	6	Specify characteristics of the sources of evidence used as eligibility criteria (e.g., years considered, language, and publication status), and provide a rationale.	Methods: Eligibility criteria; Supplementary Table 2
Information sources*	7	Describe all information sources in the search (e.g., databases with dates of coverage and contact with authors to identify additional sources), as well as the date the most recent search was executed.	Methods: Information sources and search strategy
Search	8	Present the full electronic search strategy for at least 1 database, including any limits used, such that it could be repeated.	Methods: Information sources and search strategy; Supplementary Table 3

[1] Tricco, A. C., Lillie, E., Zarin, W., O'Brien, K. K., Colquhoun, H., Levac, D., Moher, D., Peters, M. D., Horsley, T., & Weeks, L. (2018). PRISMA extension for scoping reviews (PRISMA-ScR): checklist and explanation. Annals of internal medicine, 169(7), 467-473.

SECTION	ITEM	PRISMA-ScR CHECKLIST ITEM	Location
Selection of sources of evidence†	9	State the process for selecting sources of evidence (i.e., screening and eligibility) included in the scoping review.	Methods: Selection process and data collection process
Data charting process‡	10	Describe the methods of charting data from the included sources of evidence (e.g., calibrated forms or forms that have been tested by the team before their use, and whether data charting was done independently or in duplicate) and any processes for obtaining and confirming data from investigators.	Methods: Selection process and data collection process
Data items	11	List and define all variables for which data were sought and any assumptions and simplifications made.	Supplementary Table 4
Critical appraisal of individual sources of evidence§	12	If done, provide a rationale for conducting a critical appraisal of included sources of evidence; describe the methods used and how this information was used in any data synthesis (if appropriate).	/
Synthesis of results	13	Describe the methods of handling and summarizing the data that were charted.	Methods: Data analysis method
RESULTS			
Selection of sources of evidence	14	Give numbers of sources of evidence screened, assessed for eligibility, and included in the review, with reasons for exclusions at each stage, ideally using a flow diagram.	Results: Study characteristics; Figure 1 Flowchart of study selection
Characteristics of sources of evidence	15	For each source of evidence, present characteristics for which data were charted and provide the citations.	Results: Study characteristics; Table 1 Study characteristics
Critical appraisal within sources of evidence	16	If done, present data on critical appraisal of included sources of evidence (see item 12).	/
Results of individual sources of evidence	17	For each included source of evidence, present the relevant data that were charted that relate to the review questions and objectives.	Results: Study findings; Figure 2 ~8; Supplementary Tables 5, 6, and 7
Synthesis of results	18	Summarize and/or present the charting results as they relate to the review questions and objectives.	Results: Synthesized framework; Figure 7 Refined CODA model
DISCUSSION			
Summary of evidence	19	Summarize the main results (including an overview of concepts, themes, and types of evidence available), link to the review questions and objectives, and consider the relevance to key groups.	Discussion
Limitations	20	Discuss the limitations of the scoping review process.	Limitations

SECTION	ITEM	PRISMA-ScR CHECKLIST ITEM	Location
Conclusions	21	Provide a general interpretation of the results with respect to the review questions and objectives, as well as potential implications and/or next steps.	Conclusions
FUNDING			
Funding	22	Describe sources of funding for the included sources of evidence, as well as sources of funding for the scoping review. Describe the role of the funders of the scoping review.	/

JBI = Joanna Briggs Institute; PRISMA-ScR = Preferred Reporting Items for Systematic reviews and Meta-Analyses extension for Scoping Reviews.

* Where *sources of evidence* (see second footnote) are compiled from, such as bibliographic databases, social media platforms, and Web sites.

† A more inclusive/heterogeneous term used to account for the different types of evidence or data sources (e.g., quantitative and/or qualitative research, expert opinion, and policy documents) that may be eligible in a scoping review as opposed to only studies. This is not to be confused with *information sources* (see first footnote).

‡ The frameworks by Arksey and O'Malley (6) and Levac and colleagues (7) and the JBI guidance (4, 5) refer to the process of data extraction in a scoping review as data charting.

§ The process of systematically examining research evidence to assess its validity, results, and relevance before using it to inform a decision. This term is used for items 12 and 19 instead of "risk of bias" (which is more applicable to systematic reviews of interventions) to include and acknowledge the various sources of evidence that may be used in a scoping review (e.g., quantitative and/or qualitative research, expert opinion, and policy document).

Supplementary Table 2 Detailed information on eligibility criteria

		Inclusion criteria	Exclusion criteria
Participants	People with frailty/pre-frailty	People with frailty/ prefrailty<br>Frailty should be measured using a validated instrument (including studies that used modified validated instruments)	Frailty was measured exclusively by physical function, disability, or comorbidity, because these conditions are not synonymous with frailty.<br>Subscale of frailty measures, social frailty, and cognitive frailty
Concept	Person-environment processes	The primary or secondary aim of studies to be an examination of any type of relationship between one or multiple P-E interaction(s) and frailty<br>Interactions between the person and the social, physical, technological, socioeconomical or service-related environment on micro/mezzo/macro level<br>Agency: goal-oriented cognition towards the environment, the use, modification, and creation of the environment (Chaudhury & Oswald, 2019).<br>Belonging: non-goal oriented cognitive and emotional processes of the connection (Chaudhury & Oswald, 2019).<br>The experience of stress due to environmental conditions (Wahl & Gerstorf, 2018).<br>The activities in the online/virtual/artificial environment	Cellular or neurobiological environments<br>Studies on medication<br>Physical programs/ intervention program without relation to environment<br>Leisure activities or daily tasks without emphasizing the environmental factors.<br>No person-environment interaction identified<br>Isolated behaviors without relation to environment.<br>Non-environmental risk for frailty (e.g., gender, age, gene, inherited disease)<br>Not empirical studies (e.g., diagnostic procedures, development, validation, or adaptations of questionnaires)
Context		Community setting (everyday living environment)	E.g., Acute care, primary health care
Types of sources		In English	No abstract or full-text available<br>Gray literature

Supplementary Table 3 Detailed information on search terms

Query	Search fields	Aspect	Description	Search terms
1<br>AND	Title, Abstract, Keywords	Participant	Frailty and frailty development	(“frailty” OR “prefrailty” OR “pre-frailty” OR “frailty index” OR “FI” OR “frailty score*” OR “frail” OR “prefrail” OR “Pre-frail”)
2	Title, Abstract, Keywords	Concept and Context	Person–environment interaction processes and community setting	(“person–environment” OR “physical environment*” OR “social environment*” OR “social capital*” OR “social network*” OR “built environment*” OR “green space” OR “greenspace” OR “air pollution” OR “housing” OR “neighborhood*” OR “neighbourhood*” OR “information and communication technology” OR “ICT” OR “socioeconomic status” OR “SES”)
Filter				2001-2024, English
(("frailty"[Title/Abstract] OR "prefrailty"[Title/Abstract] OR "pre-frailty"[Title/Abstract] OR "frailty index"[Title/Abstract] OR "FI"[Title/Abstract] OR "frailty score*"[Title/Abstract] OR "frail"[Title/Abstract] OR "prefrail"[Title/Abstract] OR "Pre-frail"[Title/Abstract]) AND ("person-environment"[Title/Abstract] OR "physical environment*"[Title/Abstract] OR "social environment*"[Title/Abstract] OR "social capital*"[Title/Abstract] OR "social network*"[Title/Abstract] OR "built environment*"[Title/Abstract] OR "green space"[Title/Abstract] OR "greenspace"[Title/Abstract] OR "air pollution"[Title/Abstract] OR "housing"[Title/Abstract] OR "neighborhood*"[Title/Abstract] OR "neighbourhood*"[Title/Abstract] OR "information and communication technology"[Title/Abstract] OR "ICT"[Title/Abstract] OR "socioeconomic status"[Title/Abstract] OR "SES"[Title/Abstract]) AND "english"[Language] AND ("english"[Language] AND 2001/01/01:2024/12/31[Date - Publication])) AND (english[Filter])				

Supplementary Table 4 Detailed information on extracted information

	Extracted information
General information	Study characteristics (authors, title, abstract, year)<br>Origin/place of investigation<br>Design
Population	Participants/target group<br>Recruitment source<br>Sample size<br>Proportion of female participants (if applicable)<br>Age recruitment criteria (if applicable)<br>Age distribution (if applicable)<br>Operationalization of frailty<br>Frailty cutoff points (if applicable)
Concept & Context	The layer of environment<br>Type of environment (according to the CODA framework)<br>Measurement of the CODA environment<br>Subjective or objective measures<br>P-E interaction categories<br>Summary of P-E processes<br>Potential mechanism<br>Study findings in the subgroups (if applicable)

Supplementary Table 5 Study title, author, design, and sample characteristics

Title	Quality Score	Author, year	Publication Year	Country/region	Continent	Study design	Participants recruitment source	Sample size	Female participants (%)	Age threshold	Age (Mean)
Associations of neighborhood built and social environments with frailty among mid-to-older aged Australian adults.	7	Abe et al., 2021	2021	Australia	Australia	Cross sectional	the How Areas in Brisbane Influence health And activity project	3419	61.8	50	60.65
Social vulnerability and aging of elderly people in the United States.	7	Abeliansky et al., 2021	2021	Germany	Europe	Prospective	the Health and Retirement Study	17602	57.2	50	68.3
Frailty prevalence and its associations with socioeconomic factors, health status, and healthcare utilization among elderly home care clients	7	Aktuna et al., 2023	2023	Turkey	Europe	Cross sectional	older people received home care services from the Bornova Municipality	298	75	65	78.6
Understanding Frailty, Functional Health and Disability among Older Persons in India: A Decomposition Analysis of Gender and Place of Resident.	7	Anand et al., 2020	2020	India	Asia	Cross sectional	the WHO-SAGE and BKPAI-2011 (the study of Building Knowledgebases for Population Ageing in India)	5347	47.3	50	
The protective effect of neighborhood composition on increasing frailty among older Mexican Americans: a barrio advantage?	7	Aranda et al., 2011	2011	USA	North America	Cross sectional	the Hispanic Established Populations for Epidemiologic Studies of the Elderly	963	33	65	80.8
The Impact of Socioeconomic Factors and Geriatric Syndromes on Frailty among Elderly People Receiving Home-Based Healthcare: A Cross-Sectional Study	6	Aravantinou-Karlatou et al., 2022	2022	Greece	Europe	Cross sectional	older adults aged 65 years old and over who received homecare in the reference region of Crete, Greece	301	63.1	65	78.5
Sedentary behaviour among older adults residing in flat and hilly neighbourhoods and its association with frailty and chronic disease status.	8	Asiamah et al., 2023	2023	Ghana	Africa	Cross sectional	community-dwelling older adults residing in the flat and hilly neighbourhoods	1209	52	50	62
Association between employee benefits and frailty in community-dwelling older adults.	8	Avila-Funes et al., 2016	2016	Mexico	North America	Cross sectional	the Mexican Study of Nutritional and Psychosocial Markers of Frailty	927	54.9	70	
Prevalence and predisposing factors of frailty and social inclusion among older adults: A cross-sectional study	8	Ayaz-Alkaya et al., 2024	2024	Turkey	Europe	Cross sectional	older adults registered in five family health centers in a city in Turkey	600	58.5	65	
Association of Life-Course Neighborhood Deprivation With Frailty and Frailty Progression From Ages 70 to 82 Years in the Lothian Birth Cohort 1936.	7	Baranyi et al., 2022	2022	UK	Europe	Prospective	the Lothian Birth Cohort 1936	1091	49.77		69.53
Disablement in Context: Neighbourhood Characteristics and Their Association With Frailty Onset Among Older Adults.	8	Caldwell et al., 2019	2019	USA	North America	Prospective	the National Social Life, Health, and Aging Project	1925	51.1	58	66.9
Indoor air pollution and frailty: A cross-sectional and follow-up study among older Chinese adults.	7	Cao et al., 2022	2022	China	Asia	Cross sectional; Prospective	the China Health and Retirement Longitudinal Study	4946	49.6	60	67.9
Age and socioeconomic gradients in frailty among older adults in India	7	Chaudhary & Chowdhary., 2018	2018	India	Asia	Cross sectional	the WHO multi-country study on Global Aging and Adult Health(SAGE)	6560	41	50	
Prevalence and associated factors of frailty among elderly people in Taiwan	7	Chen et al., 2014	2014	Taiwan	Asia	Cross sectional	the Coming of an Aging Society Study on Social Planning in Taiwan in 2025	495	48.3	65	
Screening for frailty phenotype with objectively-measured physical activity in a west Japanese suburban community: evidence from the Sasaguri Genkimon Study	7	Chen et al., 2015	2015	Japan	Asia	Cross sectional	the Sasaguri Genkimon Study	1527	61.2	65	73.3

Household Polluting Fuel Use and Frailty among Older Adults in Rural China: The Moderating Role of Healthy Lifestyle Behaviors.	8	Chen et al., 2023	2023	China	Asia	Cross sectional	the Chinese Longitudinal Healthy Longevity Survey (2018)	4535	45.8	65	82.7
Childhood and adult socioeconomic status influence on late-life healthy longevity: evidence from the Chinese longitudinal healthy longevity survey.	6	Chen, 2024	2024	China	Asia	Prospective	the Chinese Longitudinal Healthy Longevity Survey	37264	58.5	65	88.4
The Association between Frequency of Social Contact and Frailty in Older People: Korean Frailty and Aging Cohort Study (KFACS).	7	Chon et al., 2018	2018	Korea	Asia	Cross sectional	the Korean Frailty and Aging Cohort Study	1200	53.3	65	
Prevalence and population characteristics associated with frailty in a rural low socioeconomic area in Denmark: the Lolland-Falster Health Study	7	Christensen et al., 2024	2024	Denmark	Europe	Cross sectional	the Danish population study Lolland-Falster Health Study	10154	52.3	50	
Effects of cigarette smoking and secondhand smoke exposure on physical frailty development among community dwelling older adults in Japan: Evidence from a 10 year population based cohort study	7	Chu et al., 2024	2024	Taiwan	Asia	Prospective	the National Institute for Longevity Sciences-Longitudinal Study of Aging	540	47.6	65	71.4
Relationships between frailty, neighborhood security, social cohesion and sense of belonging among community-dwelling older people.	7	Cramm & Nieboer, 2013	2013	Netherlands	Europe	Cross sectional	older adults (aged 70 years) in four districts in Rotterdam.	945	56	70	77.5
The creation of age-friendly environments is especially important to frail older people	7	Cramm et al., 2018	2018	Netherlands	Europe	Cross sectional; Qualitative	older adults residing in four districts of Rotterdam	545	56	70	77
The combined effects of heatwaves, air pollution and greenery on the risk of frailty: a national cohort study.	7	Dai et al., 2024	2024	China	Asia	Prospective	the China Health and Retirement Longitudinal Study	6400	45.5	45	56.54
Effects of air pollutants exposure on frailty risk: A systematic review and meta-analysis.		Ding et al., 2024	2024	China	Asia	Systematic review		20			
The Social Environment's Relationship With Frailty: Evidence From Existing Studies.		Duppen et al., 2019	2019	Belgium	Europe	Systematic review		15			
Social Participation in the Daily Lives of Frail Older Adults: Types of Participation and Influencing Factors.		Duppen et al., 2020	2020	Belgium	Europe	Qualitative	community-dwelling older adults	38			
Identifying frailty risk profiles of home-dwelling older people: focus on sociodemographic and socioeconomic characteristics	7	Dury et al., 2017	2017	Belgium	Europe	Cross sectional	the Belgian Ageing Studies	28049	54.3	60	
Frailty prevalence and neighborhood residence in older Mexican Americans: the San Antonio longitudinal study of aging.	7	Espinoza & Hazuda, 2015	2015	USA	North America	Cross sectional	the baseline examination of the San Antonio Longitudinal Study of Aging	394	64.2	65	69.2
Characteristics of residential areas and transportational walking among frail and non-frail Dutch elderly: does the size of the area matter?	7	Etman et al 2014	2014	Netherlands	Europe	Cross sectional	the Elderly And Their Neighborhood study	408	52.9	65	
Concurrent and lagged associations of social participation and frailty among older adults.	7	Fang et al., 2022a	2022	China	Asia	Cross sectional; Prospective	the China Health and Retirement Longitudinal Study	6865	49.7	60	68.15
Perceived Social Support and Associated Factors Among Community-Dwelling Older Adults With Frailty and Pre-frailty in Hangzhou, China	7	Fang et al., 2022b	2022	China	Asia	Cross sectional	adults aged 60 years and over who were in the community healthcare center and could communicate in Chinese in Hangzhou City, Zhejiang Province, China	543	58.6	60	70.99

Influence of the Amazonian context on the frailty of older adults: A population-based study.	7	Fernandes et al., 2021	2021	Brazil	South America	Cross sectional	the Study of Health and Frailty of Older Adults of the Brazilian Amazon	256	62.6	60	
Socioeconomic inequalities in frailty and frailty components among community-dwelling older citizens.	8	Franse et al., 2017	2017	Netherlands	Europe	Cross sectional	the Older Persons and Informal Caregivers Survey Minimum Dataset	26014	58.4	55	78
Neighborhood Characteristics and Frailty: A Scoping Review.	NA	Fritz et al., 2020	2020	USA	North America	Systematic review		13			
Close relationships and risk of frailty: the Hertfordshire Cohort Study	7	Gale et al., 2012	2012	UK	Europe	Cross sectional	the Hertfordshire Cohort Study	482	49.4		64.8
Social isolation and loneliness as risk factors for the progression of frailty: the English Longitudinal Study of Ageing	7	Gale et al., 2018	2018	UK	Europe	Prospective	the English Longitudinal Study of Ageing	2817	56.9	60	69.3
Association of lead and cadmium exposure with frailty in US older adults	7	Garcia-Esquinas et al., 2015	2015	USA	North America	Cross sectional	the third U.S. National Health and Nutrition Examination Survey	5272	51	60	
Non-use of information and communication technology as a predictor of frailty in postmenopausal midlife and older women.	7	Garcia-Vigara et al., 2022	2022	Spain	Europe	Cross sectional	community-dwelling post-menopausal women attending primary care centers for health checks	409	100	40	67.45
The Effect of Socioeconomic Status Across Adulthood on Trajectories of Frailty in Older Women.	7	Gardiner et al., 2016	2016	Australia	Australia	Prospective	the Australian Longitudinal Study on Women's Health	7484	100		72.5
How Neighborhood Structural and Individual Characteristics Affect Frailty Progression: Evidence from the China Health and Retirement Longitudinal Study.	7	Ge & Kwon, 2023	2023	China	Asia	Prospective	the China Health and Retirement Longitudinal Study	6238	49.6	60	68
Associations of social isolation, social participation, and loneliness with frailty in older adults in Singapore: a panel data analysis.	7	Ge et al., 2022	2022	Singapore	Asia	Cross sectional	the longitudinal Population Health Index Survey conducted in Singapore	606	57.6	60	70.9
Frailty Was Associated With Atmospheric NO2 Levels: A Geospatial Approach.	6	Gonzalez-Bautista et al., 2024	2024	Mexico	North America	Cross sectional	the National Health and Nutrition Survey	1907	58.8	50	73
Can Social Mobility Impact Frailty Trajectories of Chinese Adults in Later Life? A Nationwide Longitudinal Study	7	Guo & Yang, 2024	2024	China	Asia	Prospective	the China Health and Retirement Longitudinal Study	13239	52.1	45	58.86
Frailty Risk in Older Adults Associated With Long-Term Exposure to Ambient PM2.5 in 6 Middle-Income Countries.	8	Guo et al., 2022	2022	China	Asia	Cross sectional	the Study on global Ageing and adult health	34138	50.2	50	
Assessing the effects of air pollution and residential greenness on frailty in older adults: a prospective cohort study from China.	8	Guo et al., 2024a	2024	China	Asia	Prospective	the Chinese Longitudinal Healthy Longevity Survey	6953	50.21	65	80.77
Differential effects of size-specific particulate matter on frailty transitions among middle-aged and older adults in China: findings from the China Health and Retirement Longitudinal Study (CHARLS), 2015-2018.	8	Guo et al., 2024b	2024	China	Asia	Prospective	the China Health and Retirement Longitudinal Study	13910		45	
Impact of physical activity, protein intake and social network and their combination on the development of frailty.	7	Haider et al., 2020	2020	Austria	Europe	Cross sectional	the Study on Health, Ageing and Retirement in Europe	22226	52.4	50	63.5
Frailty and socioeconomic position: A systematic review of observational studies.		Hanlon et al., 2024	2024	UK	Europe	Systematic review		383			
Association between residential greenspace structures and frailty in a cohort of older Chinese adults.	8	He et al., 2022	2022	China	Asia	Cross sectional	the Chinese Longitudinal Healthy Longevity Survey	8776	52.88		

Contribution of socioeconomic position over life to frailty differences in old age: comparison of life-course models in a French sample of 2350 old people	7	Herr et al., 2015	2015	France	Europe	Cross sectional	the information system for loss of functional autonomy of the older adults	2350	59.4	70	83.3
Adverse effects of frailty on social functioning in older adults: Results from the Longitudinal Aging Study Amsterdam.	7	Hoogendijk et al., 2016	2016	France	Europe	Cross sectional; prospective	the Longitudinal Aging Study Amsterdam	1115	54.7	65	74.8
Trajectories of frailty and related factors of the older people in Taiwan	7	Hsu & Chang, 2015	2015	Taiwan	Asia	Prospective	the Taiwanese Longitudinal Survey on Aging	2306	44.9	64	70.7
The association between city-level air pollution and frailty among the elderly population in China.	8	Hu et al., 2020	2020	China	Asia	Prospective	the Chinese Longitudinal Healthy Longevity Survey	4746	54.3	65	
Association between social capital and frailty and the mediating effect of health-promoting lifestyles in Chinese older adults: a cross-sectional study.	7	Hu et al., 2022	2022	China	Asia	Cross sectional	the Chinese older adults in Changsha city	674	49.4	60	
Causal effects of socioeconomic traits on frailty: a Mendelian randomization study.	6	Huang et al., 2024	2024	China	Asia	Cross sectional	the summary-level genetic data from large-sample genomewide association studies				
Income and education are associated with transitions in health status among community-dwelling older people in Japan: the JAGES cohort study.	7	Ikeda et al., 2019	2019	Japan	Asia	Prospective	the Japan Gerontological Evaluation Study	65952	50.7	65	72.9
Area Deprivation Index and Frailty Among Older People With HIV.	7	Iriarte et al., 2024	2024	USA	North America	Cross sectional	older people with HIV enrolled in two cohorts from the Denver, Colorado and St. Louis, Missouri metropolitan areas.	209	81.5	50	57.7
Social position and frailty	7	John et al., 2013	2013	Canada	North America	Cross sectional	the Manitoba Study of Health and Aging	1751	58.5	65	77.5
Use of Information and Communication Technologies Among Older People With and Without Frailty: A Population-Based Survey.	7	Keränen et al., 2017	2017	Finland	Europe	Cross sectional	the Gamified Services for Elderly study	918		65	
Association of socioeconomic status measures with physical activity and subsequent frailty in older adults.	7	Kheifets et al., 2022	2022	Isreal	Asia	Prospective	the First National Health and Nutrition Survey of Older Adults Aged 65 and over in Israel	1799	53.3	65	74.6
The Association of Perceived Neighborhood Walkability and Environmental Pollution With Frailty Among Community-dwelling Older Adults in Korean Rural Areas: A Cross-sectional Study.	7	Kim et al., 2019	2019	Korea	Asia	Cross sectional	adults aged 65 and older in Gyeongnam Province, Korea	808	59	65	74.6
A Cumulative Deficit Laboratory Test-based Frailty Index: Personal and Neighborhood Associations.	7	King et al., 2017	2017	USA	North America	Cross sectional	the Duke-EPESE study of persons age 65-105	1740	66	65	78
Frailty and its association with the Mediterranean diet, life-space, and social participation in community-dwelling older people	7	Kwan et al., 2019	2019	Hong Kong	Asia	Cross sectional	older adults visiting non-government community centers in Hong Kong	263	83.7	60	77.1
The association of technology acceptance and physical activity on frailty in older adults during the COVID-19 pandemic period.	7	Kwan et al., 2023	2023	Hong Kong	Asia	Cross sectional	a snowball sampling method to invite community-dwelling older adults in Hong Kong	380	57.4	60	66.5
Neighborhood deprivation, individual socioeconomic status, and frailty in older adults.	7	Lang et al., 2009	2009	UK	Europe	Prospective	the English Longitudinal Study of Ageing	4818	55	65	
PM(2.5) air pollution contributes to the burden of frailty.	7	Lee et al., 2020	2020	Taiwan	Asia	Cross sectional	the New Taipei City Elderly Health Examination Database	20606	53.9	65	72.9
Linking early life risk factors to frailty in old age: evidence from the China	7	Li et al., 2020	2020	China	Asia	Prospective	the China Health and Retirement Longitudinal Study	6806	49.2	60	69

Health and Retirement Longitudinal Study.											
Neighborhood Effects on the Health of Chinese Older Adults: Beyond the Rural and Urban Dichotomy.	7	Li et al., 2021	2021	China	Asia	Cross sectional	the China Health and Retirement Longitudinal Study	6245	49.67	60	68.06
Internet use and frailty in middle-aged and older adults: findings from developed and developing countries	7	Li, 2024	2024	China	Asia	prospective	wave 11-wave 14 (2012 – 2018) for Health and Retirement Study (HRS); wave 1-wave 4 (2011 – 2018) for China Health and Retirement Longitudinal Study (CHARLS); wave 5-wave 8 (2013 – 2019) for Health Ageing and Retirement in Europe (SHARE); wave 6-wave 9 (2012 – 2018) for Mexican Health and Aging Study (MHAS)				
The Relationship Between Health Literacy, Social Support, Depression, and Frailty Among Community-Dwelling Older Patients With Hypertension and Diabetes in China.	7	Liu et al., 2020	2020	China	Asia	Cross sectional	community dwelling elders aged 65 years and older who had been diagnosed with hypertension and diabetes	637	64.8	65	72.21
Neighborhood resources associated with frailty trajectories over time among community-dwelling older adults in China.	7	Liu et al., 2022	2022	China	Asia	Prospective	the China Health and Retirement Longitudinal Study	5673	49.4	60	67.7
Links between life-course SES and frailty trajectory moderated by community environment resources: Person-environment Fit perspective.	7	Liu et al., 2023	2023	China	Asia	Prospective	the China Health and Retirement Longitudinal Study	11675		50	61.6
Association of Air Quality Improvement and Frailty Progression: A National Study across China.	7	Liu et al., 2024	2024	China	Asia	Prospective	the China Health and Retirement Longitudinal Study	12891	51.6	45	58.6
Associations of multi-faceted factors and their combinations with frailty in Japanese community-dwelling older adults: Kashiwa cohort study.	7	Lyu et al., 2022	2022	Japan	Asia	Cross sectional	the Kashiwa Cohort Study	1308	47.8	65	74.6
Household wealth, neighborhood deprivation and frailty amongst middle-aged and older adults in England: a longitudinal analysis over 15 years (2002-2017).	7	Maharani et al., 2023	2023	England	Europe	Prospective	the English Longitudinal Study of Ageing	17438	53.6	50	65.1
Frailty and Social Isolation: Comparing the Relationship between Frailty and Unidimensional and Multifactorial Models of Social Isolation.	7	Maltby et al., 2020	2020	UK	Europe	Prospective	the English Longitudinal Study of Ageing	4918	56.64	60	70.52
Frailty and Social Vulnerability in Mexican Deprived and Rural Settings.	7	Manrique-Espinoza et al., 2016	2016	Mexico	North America	Cross sectional	the Impact Evaluation Study of the social pension	558	47.5	60	
Built Environment and Frailty: Neighborhood Perceptions and Associations With Frailty, Experience From the Nagoya Longitudinal Study.	7	Martins et al., 2020	2020	Japan	Asia	Cross sectional	the Nagoya Longitudinal Study-Healthy Elderly	370	55.4	60	72.12
Objective and subjective measures of the neighborhood environment: Associations with frailty levels.	7	Martins et al., 2021	2021	Australia	Australia	Cross sectional	community-dwelling adults in Adelaide, Australia	115	56.9	60	75.53
Impacts of Physical Environment Perception on the Frailty Condition in Older People.	7	Mena et al., 2020	2020	Chile	South America	Cross sectional	participants for healthcare centers located in the Maule Region, in central Chile	284	63.4	60	73.4
Transitions between frailty states in the very old: the influence of socioeconomic status and multi-morbidity in the Newcastle 85+ cohort study.	6	Mendonca et al., 2020	2020	UK	Europe	Prospective	the Newcastle 85+ Study	696	59.4	85	

Do Walking-Friendly Built Environments Influence Frailty and Long-Term Care Insurance Service Needs?	7	Mitsutake et al., 2021	2021	Japan	Asia	Cross sectional	older adults aged 65 years or older who were all residents in Hatoyama town	2867	49.3	65	73
Perceived Control Mediates Effects of Socioeconomic Status and Chronic Stress on Physical Frailty: Findings From the Health and Retirement Study.	7	Mooney et al., 2018	2018	USA	North America	Cross sectional; Prospective	the Health and Retirement Study	5250	57.3	65	72.8
Built environments and frailty in older adults: A three-year longitudinal JAGES study.	7	Mori et al., 2022	2022	Japan	Asia	Prospective	the Japan Gerontological Evaluation Study	38829	52	65	72.2
Built Environments and Frailty in Older Adults: The JAGES Longitudinal Study Using Mediation Analysis.	7	Mori et al., 2023	2023	Japan	Asia	Prospective	adults aged 65 years or older living in 22 municipalities in 9 prefectures who were not certified as requiring support or care	33174		65	
Prevention of frailty in relation with social out-of-home activities in older adults: results from the Survey of Health, Ageing, and Retirement in Europe.	7	Mümken et al., 2024	2024	Germany	Europe	Prospective	the Survey of Health, Ageing, and Retirement in Europe	13456	58.5	50	65.94
Relationship between Perceived Indoor Temperature and Self-Reported Risk for Frailty among Community-Dwelling Older People.	8	Nakajima et al., 2019	2019	Japan	Asia	Cross sectional	older adults visiting an outpatient rehabilitation facilities in Kochi, Osaka,	342	62.9	65	81.74
Regional differences in frailty among older adults with type 2 diabetes: a multicenter cross-sectional study in Japan.	7	Nishimura et al., 2024	2024	Japan	Asia	Cross sectional	older adults with presence of type 2 diabetes, age 6080 years, and unimpaired basic ADL from the frailty prevention program for older adults with diabetes study	421	45.6	60	69.8
Role of Individual Social Capital in the Association of Physical Frailty With Functional Ability Among Older Adults.	7	Noguchi & Shang, 2024	2024	Japan	Asia	Cross sectional	visitors to public facilities	522	78	60	74.1
Association between community-level social capital and frailty onset among older adults: a multilevel longitudinal study from the Japan Gerontological Evaluation Study (JAGES).	7	Noguchi et al., 2022	2022	Japan	Asia	Prospective	non- institutionalized older adults from the Japan Gerontological Evaluation Study	21940	51.2	60	71.8
A Sense of Control and Wellbeing in Older People Living with Frailty: A Scoping Review.		Nyende et al., 2023	2023	Greece	Europe	Systematic review		34			
Frailty in older adults and their association with social determinants of Health. The SABE Colombia Study.	7	Ocampo-Chaparro et al., 2019	2019	Colombia	South America	Cross sectional	the SABE Health, Wellbeing, and Aging Colombia project	24553	58.4	60	69.3
Social support, stressors, and frailty among older Mexican American adults	7	Peek et al., 2012	2012	USA	North America	Prospective	the Hispanic Established Populations for the Epidemiologic Study of the Elderly	2438	58	65	75.13
Housing conditions and risk of physical function limitations: a prospective study of community-dwelling older adults.	7	Pérez-Hernández et al., 2018	2018	Spain	Europe	Prospective	the study on Nutrition and Cardiovascular Risk in Spain	1602	49.1	60	70.7
Difference-Making Pathways to Frailty Through Social Factors: A Configurational Analysis.	6	Pollak et al., 2024	2024	USA	North America	Cross sectional	the Rush Memory and Aging Project a longitudinal, clinical pathologic study of chronic conditions of aging	1071	75.8	65	79.3
Is frailty associated with life-space mobility and perceived autonomy in participation outdoors? A longitudinal study.	6	Portegijs et al., 2016	2016	Finland	Europe	Prospective	the Life-space mobility in old age cohort study	753	64	75	80.4
Social Participation's Association with Falls and Frailty in Malaysia: A Cross-Sectional Study.	7	Risbridger et al., 2022	2022	Malaysia	Asia	Cross sectional	the Malaysian Elders Longitudinal Research study	1383	57.1	55	68.5
The Association of Neighborhood Characteristics and Frailty in Childhood	7	Schwartz et al., 2023	2023	USA	North America	Cross sectional	the St. Jude Lifetime Cohort study	3806	46.7		55

Cancer Survivors: A Report from the St. Jude Lifetime Cohort Study.											
Differences in the Association of Neighborhood Environment With Physical Frailty Between Urban and Rural Older Adults: The Korean Frailty and Aging Cohort Study (KFACS).	7	Seo et al., 2021	2021	Korea	Asia	Cross sectional	the Korean Frailty and Aging Cohort Study	2593	51	70	76
Understanding frailty among older people living in old age homes and the community in Nepal: A cross-sectional study	7	Shah et al., 2021	2021	Nepal	Asia	Cross sectional	older adults in old age homes and communities of Kathmandu valley in NepaL	694	51.1	60	72.6
Psychosocial stressors associated with frailty in community-dwelling older adults in the United States.	7	Shakya et al., 2024	2024	USA	North America	Cross sectional	the Health and Retirement Study	7679	57.9	65	74.9
Association between socioeconomic position trajectories and frailty among elderly people in Taiwan.	7	Shiau et al., 2023	2023	Taiwan	Asia	Cross sectional	the SEBAS 2006 from Taiwan Longitudinal Study of Aging	1284	47.12	53	65.79
Frailty Related to the Exposure to Particulate Matter and Ozone: The Korean Frailty and Aging Cohort Study.	7	Shin & Choi, 2021	2021	Korea	Asia	Cross sectional	the Korean Frailty and Aging Cohort Study	3014	52.4	70	76.8
Associations between information and communication technology use and frailty in community-dwelling old-old adults: results from the ILSA-J.	7	Shiratsuchi et al., 2024	2024	Japan	Asia	Cross sectional	the Integrated Longitudinal Studies on Aging in Japan	2893	62.1	75	79.6
Biomass fuel usage for cooking and frailty among older adults in China: a population-based cohort study.	7	Song et al., 2023	2023	China	Asia	Prospective	the Chinese Longitudinal Healthy Longevity Survey	4643	46.3	65	80.9
Frailty and social isolation before and during the coronavirus disease 2019 pandemic among older adults: A path analysis.	7	Su et al., 2024	2024	Japan	Asia	Cross sectional	community-dwelling older adults in Hokkaido and Tokyo	852	57.9	65	77.3
Socioeconomic status is associated with frailty: the Women's Health and Aging Studies.	7	Szanton et al., 2010	2010	USA	North America	Cross sectional	the Women Health and Aging Studies	727	100	65	74.2
Social factors associated with reversing frailty progression in community-dwelling late-stage elderly people: An observational study.	7	Takatori et al., 2021	2021	Japan	Asia	Prospective	community-dwelling older adults older than 75 years old, in Ikoma City, Nara Prefecture, Japan	5050	50.3	75	79.4
Narratives of older persons' frailty and physical activity in relation to environmental landscapes and time		Thinuan et al., 2020	2020	Thailand	Asia	Qualitative	older persons living in the province of Lampang in the north of Thailand	13		60	70.47
Association between health risks and frailty in relation to the degree of housing damage among elderly survivors of the great East Japan earthquake.	8	Tsubota-Utsugi et al., 2018	2018	Japan	Asia	Prospective	the Research project for prospective Investigation of health problems Among Survivors of the Great East Japan Earthquake	2261	54.7	65	73.2
Gender and Socioeconomic Inequalities in Health	7	Uccheddu et al., 2019	2019	Netherland	Europe	prospective	the Survey of Health, Ageing and Retirement in Europe (SHARE) between the years 2004 – 2015	13,955	53.66	50	67.93
Friendship-related social isolation is a potential risk factor for the transition from robust to prefrailty among healthy older adults: a 1-year follow-up study.	7	Uno et al., 2021	2021	Japan	Asia	Prospective	the Nagoya Longitudinal Study for Healthy Elderly	229	53.7	60	69.3
Life Course Socioeconomic Conditions and Frailty at Older Ages.	7	Van der Linden et al., 2020	2020	Switzerland	Europe	Prospective	the longitudinal Survey of Health, Ageing, and Retirement in Europe	23358	54.2	50	64.6
Association Between Pollution and Frailty in Older People: A Cross-Sectional Analysis of the UK Biobank.	7	Veronese et al., 2023	2023	Italy	Europe	Cross sectional	the UK Biobank	220079	52.8	60	64.7
Frailty and socioeconomic status: a systematic review.		Wang & Hulme, 2021	2021	UK	Europe	Systematic review		21			

Social capital, depressive symptomatology, and frailty among older adults in the western areas of China.	7	Wang et al., 2023	2023	China	Asia	Cross sectional	the Health Examination for Urban and Rural Residents program	2591	54.3	60	
Socioeconomic support, quality of life, and prognosis of frailty among the older adults	7	Wang et al., 2024	2024	China	Asia	Prospective	the Chinese Longitudinal Healthy Longevity Survey	13859	54.9	65	85.8
Social contexts and cross-national differences in association between adverse childhood experiences and frailty index.	7	Wang, 2023	2023	China	Asia	Prospective	the Survey of Health, Ageing and Retirement in Europe, China Health and Retirement Longitudinal Study	43928		50	
Association of unmet basic resource needs with frailty and quality of life among older adults with cancer-Results from the CARE registry.	7	Williams et al., 2023	2023	USA	North America	Cross sectional	the CARE registry prospectively enrolls older adults with cancer.	494	36.4	60	69
Social determinants of frailty	7	Woo et al., 2005	2005	Hong Kong	Asia	Cross sectional	random sampling from a registered list of all recipients of old age and disability allowances	2032	50.8	70	80
Association between City-Level Particulate Matter Exposure and Frailty among Middle-Aged and Older Adults in China	7	Wu et al., 2024	2024	China	Asia	Cross sectional	the China Health and Retirement Longitudinal Study	15611			
Association between Age-Friendliness of Communities and Frailty among Older Adults: A Multilevel Analysis	7	Xu et al., 2022	2022	China	Asia	Cross sectional	older adults aged 65 years or older from 43 communities in four cities in China	390	53.45	65	
Childhood experiences and frailty trajectory among middle-aged and older adults in China.	7	Yan et al., 2022	2022	China	Asia	Prospective	the China Health and Retirement Longitudinal Study	10963	53	45	58.29
Associations between lifestyle, physical and social environments and frailty among Chinese older people: a multilevel analysis.	7	Ye et al., 2018	2018	China	Asia	Cross sectional	community-living residents recruited in the Shanghai Healthy City Survey in 2017	2559	57.4	60	66.12
The Identification of Hereditary and Environmental Determinants of Frailty in a Cohort of UK Twins.	6	Young et al., 2016	2016	UK	Europe	Cross sectional; Prospective	the St. Thomas UK Adult Twin Registry	3375		45	59
Is Neighborhood Green Space Associated With Less Frailty? Evidence From the Mr. and Ms. Os (Hong Kong) Study.	7	Yu et al., 2018	2018	Hong Kong	Asia	Prospective	the Mr. and Ms. Os study	3240	49.2	65	72.2
Socioeconomic Inequalities in Frailty in Hong Kong, China: A 14-Year Longitudinal Cohort Study.	7	Yu et al., 2020	2020	Hong Kong	Asia	Prospective	the Mr. and Ms. Os study	694	50.1	65	71.2
Adaptative thermal comfort analysis in the elderly based on Fried frailty classification in residential buildings during summer	7	Zhou et al., 2024	2024	China	Asia	Cross sectional	Older individuals residing in 58 residential apartment buildings in Chongqing, China	394		65	73
Residential Greenness and Frailty Among Older Adults: A Longitudinal Cohort in China	7	Zhu et al., 2020	2020	China	Asia	Prospective	the Chinese Longitudinal Healthy Longevity Survey (CLHLS)	16,238	55.6	65	83
Home environment and frailty in very old adults	7	Zimmermann et al., 2021	2021	Germany	Europe	Cross sectional	the study of Quality of Life and Well-Being of the Very Old in North Rhine-Westphalia	1577	63.2	80	84.9
Note: Blank cells indicate that the original publication did not report this information.											

Supplementary Table 6 Frailty measurements

Author, year	Theoretical model	Name of P-F/Number of deficits/Name of multidimensional frailty indicator	Frailty cutoff points (if applicable)	Frailty prevalence (if applicable)
Abe et al., 2021	Deficit accumulation model	32	0.35	12
Abeliansky et al., 2021	Deficit accumulation model	38		
Aktuna et al., 2023	Physical frailty model	FRAIL	3	20
Anand et al., 2020	Deficit accumulation model	40		
Aranda et al., 2011	Physical frailty model	a modified version of 4-iterm fried frailty phenotype with weight loss, exhaustion, walking speed, and grip strength	2	37.1
Aravantinou-Karlatou et al., 2022	Physical frailty model	the SHARE-Frailty Instrument	3	38.5
Asiamah et al., 2023	Multidomain model	the Tilburg frailty indicator		
Avila-Funes et al., 2016	Physical frailty model	frailty phenotype	3	14.1
Ayaz-Alkaya et al., 2024	Multidomain model	the Tilburg frailty indicator	5	60.5
Baranyi et al., 2022	Deficit accumulation model	30	0.2	
Caldwell et al., 2019	Physical frailty model	fried phenotype	3	18.4
Cao et al., 2022	Deficit accumulation model	20	0.25	66.2
Chaudhary & Chowdhary., 2018	Physical frailty model	frailty phenotype	3	19.5

Chen et al., 2014	Physical frailty model	frailty phenotype	3	8.3
Chen et al., 2015	Physical frailty model	physical phenotype which physical activity was objectively measured with a tri-axial accelerometer.	3	9.3
Chen et al., 2023	Deficit accumulation model	38		
Chen, 2024	Deficit accumulation model	38	0.1	
Chon et al., 2018	Physical frailty model	frailty phenotype	3	9
Christensen et al., 2024	Physical frailty model	five-item SHARE-FI	3	6.7
Chu et al., 2024	Physical frailty model	frailty phenotype	3	6.2
Cramm & Nieboer, 2013	Multidomain model	the Tilburg frailty indicator	5	48.8
Cramm et al., 2018	Multidomain model	the Tilburg frailty indicator	5	43.7
Dai et al., 2024	Deficit accumulation model	39	0.21	14.7
Duppen et al., 2019	Multidomain model	the Comprehensive Frailty Assessment Instrument		
Dury et al., 2017	Multidomain model	the Comprehensive Frailty Assessment Instrument		
Espinoza & Hazuda, 2015	Physical frailty model	frailty phenotype	3	15.6
Etman et al 2014	Multidomain model	the Identification of Seniors at Risk of Functional Loss Scale	2	24.8
Fang et al., 2022a	Deficit accumulation model	39		
Fang et al., 2022 b	Physical frailty model	FRAIL scale	3	24.9

Fernandes et al., 2021	Physical frailty model	frailty phenotype	3	9.4
Franse et al., 2017	Deficit accumulation model	45		
Gale et al., 2012	Physical frailty model			7.3
Gale et al., 2018	Deficit accumulation model and Physical frailty model	52		
Garcia-Esquinas et al., 2015	Physical frailty model	frailty phenotype	3	7.1
Garcia-Vigara et al., 2022	Physical frailty model	frailty phenotype	3	33
Gardiner et al., 2016	Physical frailty model	FRAIL scale	3	22.1
Ge & Kwon, 2023	Deficit accumulation model	61		
Ge et al., 2022	Multidomain model	the Clinical Frailty Scale	3	
Gonzalez-Bautista et al., 2024	Deficit accumulation model	31	0.36	21.9
Guo & Yang, 2024	Deficit accumulation model	32	0.25	
Guo et al., 2022	Deficit accumulation model	40	0.2	
Guo et al., 2024a	Deficit accumulation model	39	0.25	21.7
Guo et al., 2024 b	Deficit accumulation model	39	0.21	14.9
Haider et al., 2020	Physical frailty model	frailty phenotype	3	3.9
He et al., 2022	Deficit accumulation model	39	0.21	

Herr et al., 2015	Deficit accumulation model	43		
Hoogendijk et al., 2016	Physical frailty model	frailty phenotype	3	12.4
Hsu & Chang, 2015	Physical frailty model	frailty phenotype	3	12.7
Hu et al., 2020	Deficit accumulation model	39		
Hu et al., 2022	Multidomain model	the Tilburg frailty indicator	5	
Huang et al., 2024	Deficit accumulation model	49		
Ikeda et al., 2019	Multidomain model	the Kihon Checklist (KCL); 25 item	8	24.7
Iriarte et al., 2024	Physical frailty model	frailty phenotype	3	68
John et al., 2013	Deficit accumulation model	40		
Keränen et al., 2017	Physical frailty model	Study of Osteoporotic Fractures Index; 3 items	2	7.1
Kheifets et al., 2022	Physical frailty model	frailty phenotype	3	14
Kim et al., 2019	Multidomain model	the Kaigo-Yobo Checklist	4	35.5
King et al., 2017	Deficit accumulation model	28	0.2	
Kwan et al., 2019	Physical frailty model	frailty phenotype	3	22.1
Kwan et al., 2023	Physical frailty model	FRAIL scale	3	4.5
Lang et al., 2009	Deficit accumulation model	58		

Lee et al., 2020	Physical frailty model	frailty phenotype	3	5.2
Li et al., 2020	Physical frailty model	frailty phenotype	3	8.1
Li et al., 2021	Physical frailty model	FRAIL scale	3	
Li, 2024	Deficit accumulation model	28-30		
Liu et al., 2020	Multidomain model	The Tilburg frailty indicator	4	42.2
Liu et al., 2022	Deficit accumulation model	39		
Liu et al., 2023	Deficit accumulation model	39		
Liu et al.,2024	Deficit accumulation model	53		
Lyu et al., 2022	Physical frailty model	frailty phenotype	3	8.7
Maharani et al., 2023	Deficit accumulation model	60	0.25	11.7
Maltby et al., 2020	Deficit accumulation model	52		
Manrique-Espinoza et al., 2016	Physical frailty model	frailty phenotype	3	8.6
Martins et al., 2020	Deficit accumulation model	54	0.21	11.9
Martins et al., 2021	Physical frailty model	FRAIL	3	33
Mena et al., 2020	Physical frailty model	frailty phenotype	3	34.1
Mendonca et al., 2020	Physical frailty model	frailty phenotype	3	24.9

Mitsutake et al., 2021	Multidomain model	the Kaigo-Yobo Checklist		13.5
Mooney et al., 2018	Physical frailty model	frailty phenotype	3	6.8
Mori et al., 2022	Multidomain model	KCL	8	7.1
Mori et al., 2023	Multidomain model	KCL	8	6.7
Mümken et al., 2024	Multidomain model	the Edmonton Frail Scale	8	3.37
Nakajima et al., 2019	Multidomain model	the Kaigo-Yobo Checklist		
Nishimura et al., 2024	Multidomain model	KCL	8	20.14
Noguchi & Shang, 2024	Physical frailty model	frailty phenotype	3	6.5
Noguchi et al., 2022	Multidomain model	KCL	8	2.8
Ocampo-Chaparro et al., 2019	Physical frailty model	frailty phenotype	3	17.9
Peek et al., 2012	Physical frailty model	frailty phenotype	3	15.7
Pérez-Hernández et al., 2018	Physical frailty model	frailty phenotype	3	4.2
Pollak et al., 2024	Deficit accumulation model	40		
Portegijs et al., 2016	Physical frailty model	frailty phenotype	3	4
Risbridger et al., 2022	Physical frailty model	frailty phenotype	3	9.3
Schwartz et al., 2023	Physical frailty model	frailty phenotype	3	8.75

Seo et al., 2021	Physical frailty model	frailty phenotype	3	7.1
Shah et al., 2021	Multidomain model	the Groningen Frailty Indicator	4	56.3
Shakya et al., 2024	Physical frailty model	frailty phenotypes	3	21.9
Shiau et al., 2023	Physical frailty model	frailty phenotypes	3	18.39
Shin & Choi, 2021	Multidomain model	the Korean Frailty Scale ; The KFS consists of six questionnaires that assess physical frailty, psychological frailty, and social frailty	3	21.1
Shiratsuchi et al., 2024	Physical frailty model	frailty phenotype	3	7.2
Song et al., 2023	Deficit accumulation model	46	0.21	19.9
Su et al., 2024	Physical frailty model	frailty screening index	3	19
Szanton et al., 2010	Physical frailty model	frailty phenotype	3	10
Takatori et al., 2021	Multidomain model	KCL, 25-item		18.7
Thinuan et al., 2020	Physical frailty model	frailty phenotype	3	
Tsubota-Utsugi et al., 2018	Multidomain model	the Kihon Checklist; 20 item	5	22.6
Uccheddu et al., 2019	Deficit accumulation model	40		
Uno et al., 2021	Physical frailty model	frailty phenotype	3	21.4
Van der Linden et al., 2020	Physical frailty model	frailty phenotype	3	7.9
Veronese et al., 2023	Physical frailty model	frailty phenotype	3	5

Wang et al., 2023	Physical frailty model	FRAIL scale	3	24
Wang et al., 2024	Physical frailty model	frailty phenotype	3	26.3
Wang, 2023	Deficit accumulation model	35		
Williams et al., 2023	Deficit accumulation model	44	0.35	29
Woo et al., 2005	Deficit accumulation model	62		
Wu et al., 2024	Deficit accumulation model	39	0.21	23.51
Xu et al., 2022	Multidomain model	Chinese frailty screening-10 Scale (CFS-10)	5	13.64
Yan et al., 2022	Deficit accumulation model	32		
Ye et al., 2018	Physical frailty model	FRAIL scale	3	16.9
Young et al., 2016	Deficit accumulation model	39		
Yu et al., 2018	Physical frailty model	frailty phenotype	3	
Yu et al., 2020	Physical frailty model	frailty phenotype	3	30.4
Zhou et al., 2024	Physical frailty model	frailty phenotype	3	21
Zhu et al., 2020	Deficit accumulation model	39	0.21	
Zimmermann et al., 2021	Physical frailty model	frailty phenotype	3	18.7
Note: Blank cells indicate that the original publication did not report this information.				

Supplementary Table 7 Person-Environment interaction findings and summaries

Author, year	CODA components	Details of the component	Summary of P–E processes	Main study findings
Abe et al., 2021	Social environment; Physical environment	Objective measures of the physical environment were derived within a 1-km network buffer around participants' residential addresses, capturing residential density, street connectivity, land-use diversity, park availability, and access to public transport. The social environment was assessed subjectively using neighborhood safety and social cohesion. Neighborhood safety reflected perceived crime levels, safety in parks and on streets, and safety when walking home from public transport stops. Social cohesion was measured through perceived shared values, trust, and the quality of social relationships within the community.	The study reported that higher land-use mix at the neighborhood level was associated with lower odds of being frail, and that higher levels of safety and social cohesion were consistently associated with lower odds of being frail among community-dwelling older people in Brisbane. Areas with high street connectivity, when not designed to be pedestrian friendly, were associated with increased frailty among older adults due to safety concerns and a lack of diverse land use.	A one–standard deviation increase in street connectivity within a 1-km network buffer around participants' homes and at the neighborhood level was associated with 23% and 13% higher odds of frailty, respectively. In contrast, a one–standard deviation increase in neighborhood land-use mix was associated with 12% lower odds of frailty. Higher perceived neighborhood safety and social cohesion were consistently linked to reduced odds of being frail.
Abeliansky et al., 2021	Social environment	Social vulnerability index (SVI): consists of social support, social isolation, social engagement, subjective wellbeing.	Individuals with a greater initial SVI may have less incentive to leave the house, face less cognitive challenges, and experience more stress from perceived loneliness. As a result, initially mild health deficits may develop. The increase in health deficits then leads to a faster development of new health deficits due to the self-productive nature of health deficit accumulation.	Later-born cohorts, particularly those born in the late 1940s or afterwards, showed higher levels of social vulnerability (SVI). This pattern suggests important policy implications: the link between the "quiet revolution" and a slight decline in SVI in later life indicates that social ties formed outside the workplace may be more protective against isolation than relationships with work friends and colleagues, likely because these connections are easier to sustain after retirement. Furthermore, the self-perpetuating nature of deficit accumulation means that small initial differences in social vulnerability can widen over the life course, intensifying inequality as people age.
Aktuna et al., 2023	Care/service	The number of emergency room visits, primary care admissions, secondary care admissions, and hospitalization days in the last year were collected.		Regular drug use, longer total hospital stay, and more frequent admissions were associated with higher frailty risk. This may reflect polypharmacy, poorer medication adherence, drug–drug interactions, and adverse effects, as well as a shared underlying burden of comorbid disease.
Anand et al., 2020	SES	individual level education and wealth		Education, wealth, working status, marital status and physical activity were significantly related to the gender gap in frailty. Wealth index and education were associated with differences in frailty between rural and urban areas.
Aranda et al., 2011	Social environment	Financial strain was defined as having trouble paying bills. Care was defined as having private insurance or Medicare.	Living in areas where individuals share similar identities fosters greater social capital. Household clusters and neighborhoods of like-minded residents strengthen cultural ties and sustain exchange relationships among kin. High neighborhood cohesion supports ethnic economies and institutions, facilitates financial and in-kind exchanges across social classes, and provides a trusted cultural environment grounded in shared language and identity. Such areas offer access to formal and informal networks, emotional support, and lifelong care activities, including elder care. Dense household clusters with rich 'funds of knowledge' further promote material survival and community resilience.	Subjects living in ethnically dense Mexican American neighborhoods who had medical insurance were at lower risk of increasing frailty than those without insurance.
Aravantinou-Karlatou et al., 2022	SES	Socioeconomic factors included annual individual income (≤4,500 euros vs. >4,500 euros) and educational level (no formal education, high school, bachelor's degree or higher).		The finding suggests that older people with higher individual incomes were less likely to present with frailty; as higher education levels were associated with frailty, suggesting that

				higher education levels may act as a protective factor, although the association was not significant in the fully adjusted model.
Asiamah et al., 2023	Physical environment	The terrain of the neighborhood was classified as either hilly or flat.	In the hilly neighborhood, higher frailty was associated with higher sedentary behavior.	The association between sedentary behavior and chronic disease status was significant in both neighborhoods, but this relationship was stronger in the hilly neighborhood. Older adults in the flat neighborhood reported lower sedentary behavior at higher frailty, but those in the hilly neighborhood reported higher sedentary behavior at higher frailty.
Avila-Funes et al., 2016	SES	Employee benefits were established according to eight characteristics: bonus, profit sharing, pension, health insurance, food stamps, housing credit, life insurance, and Christmas bonus.		Employee benefits were inversely associated with the frail subgroup. Frail participants were older and more likely to be female. Women are less likely than men to have public or private disability insurance.
Ayaz-Alkaya et al., 2024	Physical environment	Satisfaction with the home living environment		Satisfaction with their home living environment was found to be predisposing factors for both frailty and social inclusion.
Baranyi et al., 2022	SES	Neighborhood Social Deprivation (NSD) was measured across childhood and mid-life. NSD in 1941–1971 included indicators such as population density, overcrowding, infant mortality, the percentage of households renting their accommodations, and the availability of amenities. NSD in 1981–2011 was based on the Carstairs index of deprivation, including male unemployment, overcrowding, car ownership, and social class.	Living in socially deprived neighborhoods can increase health risks and frailty by generating chronic stress and restricting access to shared resources. Social cohesion and active participation, in contrast, appear to protect against frailty by strengthening social ties and helping residents manage stress more effectively. When neighborhoods are perceived as unsafe, frailty risk may rise because people experience heightened stress, avoid outdoor activities, and adopt less effective coping strategies. From an accumulation perspective, exposure to deprived neighborhoods in early life can disrupt stress regulation and epigenetic processes, setting the stage for adverse outcomes in later adulthood. Gendered social roles may further shape vulnerability: boys may be at greater risk due to looser supervision and stronger neighborhood influences on behavior and aspirations, whereas girls have historically spent more time in domestic roles.	Among males, the impact of living in socially deprived areas in childhood and mid- to late adulthood contributed to higher frailty in older age. Among females, higher NSD in mid- to late adulthood was associated with faster frailty progression.
Caldwell et al., 2019	Social environment; Physical environment	Structural characteristics included neighborhood racial/ethnic density, socioeconomic disadvantage, and residential instability. Socioeconomic disadvantage was derived from a PCA component capturing the tract proportions of residents living below the federal poverty level, households receiving public assistance, adults with a high school education or less, and unemployed people aged 16 years or older. Residential instability was based on the tract proportions of residents who had moved in the past year and non–owner-occupied dwellings. Social process variables comprised physical disorder, measured by self-rated maintenance of buildings and streets, and social cohesion, assessed through self-reported mutual help, friendliness, shared values, and trust.	Physical disorder refers to visible deterioration in buildings and streets, signaling socioeconomic challenges and weak social control. Social cohesion involves trust and solidarity, fostering community support. High residential instability leads to unfamiliar faces and weak social networks, limiting resource exchange. Older adults in such areas may struggle with disabilities and daily activities. Physical disorder discourages older adults from leaving home, while socially cohesive neighborhoods promote shared responsibility for safety and well-being, improving quality of life and belonging.	In models excluding social process variables, residing in neighborhoods with a higher density of African American residents and greater residential instability was associated with increased odds of frailty. In models that incorporated social process variables, living in areas with mild socioeconomic disadvantages, the highest levels of residential instability, and greater physical disorder was likewise linked to higher odds of frailty, whereas greater social cohesion was associated with lower odds of being frail.
Cao et al., 2022	Physical environment	Indoor air pollution is assessed by cooking fuel type, solid or clean.	Adverse health outcomes caused by air pollution exposure are well known to involve inflammatory reaction, oxidative stress, and epigenetic modifications.	Participants using solid fuel were more likely to develop frailty than those using clean fuel, as assessed by either phenotype or the index. Based on a 4-year follow up, use of solid fuel for cooking might only be a risk factor for phenotypic frailty.

Chaudhary & Chowdhary., 2018	SES	education and household wealth index	High SES is a protective factor against health shortfalls as it promotes healthier lifestyles and better health practices High SES is an essential factor in preserving reserve capacities and slows down the movement of functional limitations and health declines after some time.	The number of frailty indicators was positively associated with lower income and education levels in the case of controlling and not controlling for confounders. Also, among the higher age groups, individuals with low SES had higher chances of being frail.
Chen et al., 2014	Social environment	Environmental factors included living arrangements and perceived social support, as well as participation in social and community activities such as group gatherings, leisure pursuits, religious services, visiting friends and relatives, and informal conversations with neighbors.		This study indicates that the factors jointly influencing prefrailty and frailty are age, diabetes, depressive syndrome, and the number of activities in which the participants were involved.
Chen et al., 2015	Social environment	The social networks were measured with the Japanese version of the Lubben Social Network Scale.		Older adults engaged in social activities are less likely to become frail, as frequent social participation helps maintain physical and mental fitness, compensating for age-related decline. Withdrawal from social activities may precede frailty, reflecting behavioral adaptation to declining physiological reserve. Interestingly, frailty showed no association with socioeconomic factors like education, income, employment, or housing tenure. This may be due to Japan’s universal health coverage and equitable socioeconomic conditions that support health maintenance, especially among the oldest elders.
Chen et al., 2023	Physical environment	Household polluting fuel use (HPFU) included kerosene, coal, charcoal, and firewood.		Use of household polluting fuels (HPFU) was significantly associated with higher frailty index scores. Healthy lifestyle behaviors, such as engaging in social activities and maintaining a nutritious diet, significantly attenuated this relationship, suggesting that older adults exposed to household air pollution from HPFU may face a lower risk of healthily when they eat healthily and remain socially active. The environmental health burden of HPFU was especially pronounced among women, individuals with no formal education, and those with low socioeconomic status.
Chen, 2024	SES	Childhood socioeconomic status (SES) factors include urban birth, access to adequate nutrition, father's occupation, and quality of medical care received. Adulthood SES is influenced by educational attainment, household income and wealth, current urban-rural residence, pension status, and access to healthcare services.	Childhood and adulthood socioeconomic status (SES) and frailty can be interpreted through both the critical period and accumulation models. Childhood SES appears to exert a stronger protective effect on healthy longevity in later life than higher SES attained in adulthood, indicating that early-life advantages lay a durable foundation for better health trajectories. Even though living standards for many older Chinese adults have improved markedly in midlife and old age, these later-life gains do not fully offset the detrimental effects of socioeconomic disadvantage experienced in childhood on healthy longevity.	Favorable childhood SES and lifelong accumulation of SES advantages protect against frailty.
Chon et al., 2018	Social environment	frequency of contact with family, friends, or neighbors	It is possible that people perceive their contact with friends as being more supportive compared to family or neighbors, resulting in more beneficial outcomes from contacting one's friends.	People who maintain less frequent contact with others are at greater odds of prevalent frailty compared to people who maintain better contact frequency. The contact frequency was considered separately for friends, family, or neighbors, and different type of contact was associated with frailty differently. Of the three types of contact, contact frequency with friends was most significantly associated with frailty.
Christensen et al., 2024	Social environment	Family social contact includes the frequency of seeing one’s children, the availability of support when needed, and the impact of financial difficulties on family dynamics.		Low frequency of seeing one's children is associated with frailty.

Chu et al., 2024	Physical environment	Secondhand smoke exposure in family and in the office, transportation or conference room.	Chronic inflammation is a popular explanation for the link between smoking and frailty. Elevated levels of inflammatory markers, such as interleukin-6 and C-reactive protein, have been found among both frail older adults and smokers. There also exists an association between smoking and lipid peroxidation, with lipid peroxidation playing a role in inflammatory processes and contributing to oxidative stress.	Participants exposed to both smoking and secondhand smoke had a higher risk of physical frailty than non-smokers without secondhand smoke exposure. Smokers exposed to secondhand smoke were associated with more risk of physical frailty compared with smokers without secondhand smoke exposure.
Cramm & Nieboer, 2013	Social environment	The neighborhood social cohesion and sense of belonging scale with 15 items. The perceived neighborhood security assessed with four items the neighborhood is quiet and peaceful, the neighborhood is spacious and roomy, the neighborhood is safe, and the neighborhood is orderly with good public security.	The community environment might provide effective ways to induce healthy behavior. Social cohesion and a sense of belonging might prevent frailty by influencing health-related behavior through the adoption of health-promoting activities and exerting social control over unhealthy behavior. Higher levels of social cohesion and sense of belonging may also result in higher degrees of social activity.	Prevalence of frailty might be higher in urban areas. Feeling secure in the neighborhood and being socially cohesive and having a sense of belonging to the neighborhood protect against frailty after controlling for age, sex, marital status and education.
Cramm et al., 2018	Social environment; Physical environment	There are 24 items representing eight domains outlined by the World Health Organization (WHO) that contribute to promoting active aging. These domains include outdoor spaces and buildings, transportation, housing, social participation, respect and social approval, civic participation, communication and information, and community support and health services.	The ecological theory of ageing proposes that relocation decisions are shaped by the balance between environmental demands and individual competencies; consequently, preferences for ageing in place are expected to vary with neighborhood conditions. Neighborhoods that lack supportive physical and social features make it harder for frail older adults to access services and move around, increasing the likelihood of institutionalization. In contrast, social neighborhood characteristics such as social and civic participation, respect, and social recognition form a critical support system that enables frail older adults to remain in the community. Patterns of “missing” neighborhood features among frail older people can therefore be interpreted as indicators of which neighborhood characteristics are essential for supporting ageing in place.	Frailty is positively linked to unmet age-in-place characteristics. Physical decline can lead to losses in physical, social, and psychological domains. Accessible public transport, streets, and buildings are vital for mobility, especially for wheelchair users. Safety perceptions depend on the ability to walk outside, particularly at night. Older adults increasingly rely on neighborhood social support for daily tasks like transportation and groceries. Maintaining independence in activities such as shopping and doctor visits is especially important for older men. Over time, neighborhood features may lose importance, with comfort at home and pleasant views becoming more valued.
Dai et al., 2024	Physical environment	Heat-wave exposure was defined by daily maximum air temperatures from 2010 to 2018. Long-term air pollution was measured as the annual mean PM2.5 concentration, and greenery exposure as the yearly maximum NDVI across China.	Air pollution has been shown to facilitate the occurrence of oxidative stress, which triggers various pathways of cytotoxic expression. These variables include cellular harm, the initiation of programmed cell death pathways, increased production of transcription factors and breakdown of proteins, diminished functioning of mitochondria, and hindered repair mechanisms.	The findings revealed a significant association between heatwave exposure and the incidence of frailty. A low level of education, a proxy for socioeconomic status (SES), as a significant contributor to increased vulnerability to heatwave exposure.
Ding et al., 2024	Physical environment	air pollutants	When solid fuel is burned, it releases a variety of air pollutants, including particulate matter, NO2, SO2, CO and organic compounds, such as benzene, benzo pyrene, polycyclic aromatic hydrocarbons, which are harmful to health. By interfering with biological mechanisms such as inflammation, oxidative stress, genetic and epigenetic modifications, and homeostasis, these pollutants induce the cumulative decrease of multiple physiological systems, which leads to physical weakness while impeding successful aging.	The results showed that exposure to PM2.5 and solid fuel increased the risk of frailty.
Duppen et al., 2019	Social environment			Five key dimensions of the social environment were distinguished: social networks, social support, social participation, subjective neighborhood experience, and socioeconomic neighborhood characteristics. Social networks capture the presence of family members, satisfaction with close relationships, and feelings of missing absent significant others. Social support reflects the availability of emotional and

				practical assistance from relatives and neighbors. Social participation denotes involvement in groups, leisure pursuits, and other social activities. Socioeconomic neighborhood characteristics refer to area-level deprivation and the ethnic composition of residents. Subjective neighborhood experience encompasses perceived safety, social cohesion, sense of belonging, and enjoyment of one's home and neighborhood.
Duppen et al., 2020	Social environment	Social activities: Doing activities alone or in parallel are not considered to be social participation. Level 3 concerns socially oriented activities talking with neighbors, and Level 4 involves task-oriented activities. Level 5 activities are oriented towards helping others, and Level 6 includes society-oriented activities.	Theoretical accounts linking social participation and frailty draw on several perspectives. Disengagement Theory proposes that, following retirement, men are expected to withdraw from instrumental roles and women from socioemotional roles as part of an adaptive transition into later life. Gerotranscendence describes a developmental shift from a predominantly materialistic outlook to a more cosmic and transcendent perspective, which can be accompanied by greater life satisfaction. Socioemotional Selectivity Theory argues that individuals' perceptions of remaining time shape the goals they prioritize and the social partners they choose in order to pursue those goals.	Narratives from frail older adults reveal that social environmental factors help maintain and enhance their well-being and can stimulate social participation. Low-key participation fosters a sense of belonging in the neighborhood. The social environment can encourage new or alternative forms of participation for those with limited opportunities due to frailty. Social and physical environments are interconnected and should be considered together. Community service centers play a vital role in linking frail older adults through participation, supporting both their social engagement and overall well-being.
Dury et al., 2017	Physical environment	Change of living environment: Having moved house in the past 10 years.	The negative relationship with social frailty might imply that moving makes it challenging for older people in terms of creating new social contacts and integrating into a new neighborhood.	Moving house has been negatively associated with physical, psychological, social, and multidimensional frailty. Women who moved in the past 10 years were less likely to be environmentally frail.
Espinoza & Hazuda, 2015	SES	Neighborhood SES		Frailty prevalence was higher in the barrio than in the transitional neighborhood or suburbs. Odds of frailty were 4.15 times higher for MAs residing in the barrio compared with those residing in the suburbs.
Etman et al., 2014	Physical environment	Objective neighborhood size was defined by tracing all possible walking routes along the street network in every direction from the nearest street to each participant's residence, up to distances of 400, 800, 1,200, and 1,600 meters. Within each of these buffers, objective neighborhood characteristics—covering aesthetics, functional features, safety, and available destinations were identified using Google Maps. Self-reported transportational walking was assessed by the reported frequency of grocery shopping and visits to family and friends during the previous two weeks.		Frail persons had more destinations in their residential area within a buffer up to 1200 meters, and more functional features within a buffer up to 800 meters compared to non-frail persons. No interaction effect of frailty level and area characteristics was found for any of the buffer sizes. An increase in functional features, presence of sidewalks and benches, within a 400-meter buffer, inaesthetic, absence of litter and graffiti, within 800 and 1200 meter buffers, and an increase of one destination per buffer of 400 and 800 meters were associated with more transportational walking.
Fang et al., 2022a	Social environment	Social participation is assessed by how often individuals engage in activities such as interacting with friends, playing games or attending community clubs, helping family, friends, or neighbors, participating in sports or social clubs, joining community organizations, and volunteering or taking part in charity work.	A higher level of social participation was significantly associated with a lower level of frailty; social activities could involve older adults in physical exercises, thus enhancing their physical and functional health; social participation provides older adults with a chance of role support, which in turn improves psychological well- being.	In the fully adjusted models, frailty was negatively associated with social participation in the same wave; higher FI scores predicted lower levels of social participation in the following wave: social participation was negatively associated with frailty in the same wave; higher social participation levels predicting lower frailty levels in the following wave.
Fang et al., 2022b	Social environment; Physical environment	Social support was assessed using the Social Support Rating Scale, including subjective support (emotional satisfaction and feeling understood), objective support (practical help and social network presence), and support utilization (engagement in social activities and seeking help). Family situations were evaluated by number of children, living arrangements, financial and moral support from children, relationship quality, and companionship. Community environment factors		Lower perceived social support level was observed among both the aged pre-frail and frail people who were single. In the frail group, the educational level of college or above and living in a community with good management positively influenced subjective support and objective support.

		included accessibility of medical institutions, sports fields, and nursing homes within 15 minutes, as well as community management, availability of recreational activities, and health education.		
Fernandes et al., 2021	Physical environment	Living conditions were characterized by three indicators: housing type (wooden versus masonry construction), residence in a riverside community (yes or no), and primary means of transportation (on foot, by vehicle, or a combination of both).	Older adults living in this environment may have adopted healthier lifestyle habits, including more routine physical activity, as suggested by previous research. The housing type likely functioned as a proxy for mode of transportation while also carrying cultural meaning because wooden houses are often linked to a simpler, riverside way of life. Residents of wooden houses may therefore represent individuals who have lived in riverside communities for a longer period, further shaping their daily behaviors and mobility patterns.	Participants who had never lived in riverside communities had 3 times more chance of being frail compared to those with riverside life experience. The type of housing is another factor found in the study that reinforces the idea that the more rural lifestyle could remain among the older adults in the region, even after moving to the urban area. Those who lived in masonry houses were associated with greater frailty, regardless of income and other variables in the equation.
Franse et al., 2017	SES	Individual SES: education level.<br>Neighborhood SES: income and labor market position of persons living in each area code.	Morbidities component mediates the association between socioeconomic status and other Frailty Index components.	Persons with the lowest SES, the lowest education level or living in the most deprived neighborhoods, had the highest overall frailty and frailty component scores. The number of morbidities mediated the association between SES indicators and other frailty components.
Fritz et al., 2020	Physical environment			This review suggests that neighborhood characteristics are linked to frailty in later life. However, the included studies differed substantially in how they defined and measured neighborhoods.
Gale et al., 2012	Social environment	The Close Persons Questionnaire, which asks about negative aspects of close relationships, confiding and emotional support, and practical support in the last year.	The finding of a link between exposure to negative interactions in close relationships and risk of frailty in women provides support for the view that psychosocial factors may predispose an individual to frailty or precipitate its onset. The underlying mechanisms are uncertain but might involve high levels of stress hormones, impaired immune functioning, or chronic inflammation.	In men, there were no differences in risk of frailty according to levels of negative interactions in close relationships or social support at baseline. In women, risk of frailty increased with level of negative interactions reported at baseline.
Gale et al., 2018	Social environment	Loneliness: Participants completed the short version of the Revised UCLA loneliness scale.<br>Social isolation: being unmarried or not cohabiting, having less than monthly contact with each of children, other members of the family, and friends, and not being a member of organizations.		High levels of loneliness, but not of social isolation, increased the risk of becoming physically frail. Neither loneliness nor social isolation were associated with the rate of change in a more broadly defined frailty index. In the sample as a whole, there was no association between social isolation and risk of becoming physically frail or pre-frail, but high social isolation was associated with increased risk of becoming physically frail in men.
Garcia-Esquinas et al., 2015	Physical environment	Blood lead and Urine cadmium concentration. Blood lead concentrations were measured by graphite furnace atomic absorption spectrophotometry. Urine cadmium was measured by Zeeman effect graphite furnace atomic absorption.	Lead and cadmium are two toxic metals widely distributed in the environment that accumulate in the human body, resulting in chronic endogenous exposure tissues and they also made contribution of environmental lead and cadmium to the development of age-related chronic diseases, including cardiovascular disease.	Blood lead concentrations showed a positive dose–response relationship with frailty.
Garcia-Vigara et al., 2022	Technology	The study distinguished whether participants were regular or sporadic users of information and communication technologies (ICTs) and documented how they accessed internet services either general internet use or specifically through social media platforms.	Social media can facilitate contact, interaction, and the development of supportive social networks, which act as important buffers against frailty by promoting psychological well-being in older adults and encouraging participation in physical activity programs among middle-aged and older populations. In this way, social media capitalizes on the well-established protective effects of social interaction in preventing deterioration in health and overall well-being.	Most women included use of some form of social media as a mode of internet use. ICT use was inversely associated with frailty status in a cohort of postmenopausal midlife/older women.

Gardiner et al., 2016	SES	SES across adulthood. early adulthood SES was measured by highest level of education; midlife SES was assessed by main occupation; late-life SES was assessed by ability to manage on income.	The study suggests that disadvantage in early adulthood and mid-life does not directly persist in terms of frailty, but they may still play a role in that they influence late-life SES; these findings support the age as-leveler hypothesis	After adjusting for confounders, the effects of later life SES on trajectory group membership persisted, whereas the effects of early and mid-life SES were attenuated.
Ge & Kwon, 2023	Social environment	Community structure: community level SES, resident stability: migrant people living less than half a year in a village or community, neighborhood type, rural villages versus urban communities.	Neighborhoods with a higher concentration of older residents often develop age-oriented services and resources (e.g., health care, transportation, social programs), and these demographic clusters can coincide with denser social-support networks. Older adults in such areas tend to have better access to instrumental and emotional support, which may slow frailty progression by buffering stress and facilitating care and service use. By contrast, social disorganization theory holds that neighborhood structural disadvantage, marked by low socioeconomic status, high residential instability, and related stressors, undermines residents' ability to build social ties, exert informal social control, and sustain collective efficacy, ultimately contributing to worse health outcomes.	Frailty index trajectories were nonlinear, with rapid increases earlier in later life and a slowing rate of deficit accumulation over time. Living in neighborhoods with more older residents and in rural villages was linked to slower frailty progression. Women showed higher frailty than men at baseline but accumulated deficits more slowly, reducing the gender gap at older ages.
Ge et al., 2022	Social environment	Social isolation was assessed by the Lubben Social Network Scale-6, loneliness was assessed using the three-item UCLA Loneliness Scale.	While being isolated for a short period could be by choice and has less impact on health; loneliness, as a negative emotional feeling, may contribute to a series of physical and mental health issues including cardiovascular disease and stroke, increased stress, and depression, and cognitive decline, thereby increasing the risk of development and progression of frailty. Social participation among older adults directly increases social interactions which has the potential to reduce cognitive decline, lowers the risk of depression, and creates a sense of belonging which alleviates feeling of loneliness.	Increase in social participation was associated with lower level of frailty and feeling lonely was associated with higher level of frailty. Social isolation was not associated with frailty. Gender did not have moderating effect on these associations.
Gonzalez-Bautista et al., 2024	Physical environment	Atmospheric NO2 Levels	An mechanism could be systemic inflammation related to chronic air pollution exposure, which affects cardiovascular and cognitive function and may lead to frailty.	Frailty was positively associated with exposure to NO2 concentrations. The primary cause of NO2 emissions are anthropogenic combustion processes, for heat or electricity generation or in combustion engines. For instance, the main sources of nitrogen oxides are road- transport vehicles.
Guo & Yang, 2024	SES	life-course socioeconomic position mobility	Upward mobility such as from low SEP to high SEP can partially compensate for disadvantages early in life while the benefits of higher SEP in childhood can be diluted by subsequent downward mobility over the life course.	The study shows that frailty trajectories increased over time for all socially mobile groups. Individuals who experienced upward social mobility exhibited higher frailty trajectories compared to those with a stable high socioeconomic position (SEP), while individuals with downward social mobility had lower frailty trajectories than those with a stable low SEP. Participants who faced socioeconomic disadvantage during childhood but achieved higher SEPs in adulthood showed better frailty outcomes than those with similar childhood backgrounds who remained in the lower SEP group throughout their lives. However, individuals who experienced downward socioeconomic mobility from childhood to adulthood had worse frailty outcomes than the group with a consistently advantaged SEP throughout their lives.
Guo et al., 2022	Physical environment	Air pollution: Yearly averaged PM2.5 concentration.	Air pollution, including long-term exposure to ambient PM2.5 induces chronic systemic oxidative stress, inflammation, hormonal changes, and genetic and epigenetic modifications all of which can cause damage to cellular and molecular structures.	In rural areas, increasing PM2.5 exposure was associated with increasing odds of frailty. The gender-stratified analysis showed that the association seemed to be slightly stronger in men than in women in rural areas.

Guo et al., 2024a	Physical environment	Air pollution: PM1, PM2.5, PM10, PM10-2.5, O3, and NO2. Residential greenness: Normalized difference vegetation index.	Air pollution on frailty, including inflammatory responses, oxidative stress, mitochondrial dysfunction, and epigenetic modifications. Air pollution initially triggers a local inflammatory response and oxidative stress upon entering the lungs. Subsequently, it enters circulation and affects extrapulmonary organs, leading to systemic inflammation and oxidative stress. Frailty is closely associated with elevated levels of oxidative stress markers and reduced parameters of antioxidant defense function.	A positive association between air pollution including PM1, PM2.5, PM10, PM10-2.5, O3, and NO2, and frailty among the older adults, as well as a negative association between NDVI and frailty in this population. with more pounced Age older than 80 years; Individuals with poorer economic status and residing in rural areas. PM1, PM2.5, PM10, and PM10-2.5 play a mediating role in the estimated relationship between residential greenness and frailty.
Guo et al., 2024b	Physical environment	1- and 3-y mean concentrations of air pollutants PM2.5, PM10, carbon monoxide, ozone, sulphur dioxide and nitrogen dioxide	Inflammation and oxidative stress are key biological pathways through which particulate matter (PM) exposure contributes to adverse health outcomes. Long-term exposure to ambient PM has been linked to increased risk of several non-communicable diseases and mental disorders, including cardiovascular disease, chronic obstructive pulmonary disease, depression, and cognitive impairment, all of which are relevant components when assessing frailty in older adults.	Significant associations were found between PM concentrations and frailty transitions. For each unit increase in the 3-year average 2.5-μm PM concentrations, the risk of worsening in frailty increased in robust and pre-frail participants, while the probability of improvement in frailty in pre-frail participants decreased. In addition, the associations of PM10 and coarse fraction of PM with frailty transitions showed similar patterns.
Haider et al., 2020	Social environment	Social network satisfaction was assessed with a single item: “Overall, on a scale from 0 to 10, where 0 means completely dissatisfied and 10 means completely satisfied, how satisfied are you with the relationships you have with all the people in your social network?"	A large body of research has shown that social isolation is associated with various diseases such as coronary heart disease, depression, as well as with the general health status, and overall mortality. The higher life expectancy of females made women more likely to have lost their life partner and therefore are more prone to social isolation. Outside family and work social network are important.	The results also show that satisfaction with the social network is associated with frailty developing in women, while no association was found in men. It suggests that women are more vulnerable to social factors compared with men.
Hanlon et al., 2024	SES	socioeconomic position (SEP)		Lower socioeconomic position (SEP) was consistently linked to higher frailty prevalence, regardless of how SEP was measured, including childhood deprivation, education, occupation, housing, income, wealth, or area-based deprivation. Lower SEP was also associated with higher frailty incidence, greater odds of progressing to more severe frailty, reduced likelihood of reverting to a less frail state, and faster accumulation of health deficits.
He et al., 2022	Physical environment	County level greenness: Satellite greenspace data quantified largest patch index, area and edge mean shape index, patch cohesion index, proximity.	Greenspace may influence health by directly promoting cognitive functions and well-being, strongly related to the onset of frailty. Large area-edge and good proximity can improve frailty by increasing opportunities for physical exercise, which is a mediator of the relationship between greenspaces and frailty transitions by improving physical, cognitive, and psychological function. Good greenness connectivity maintained by green ecological corridors also provides opportunities for physical activities.	In cross-sectional analyses, the study finds negative dose-response relationships for greenspace structures and frailty. Stronger significant association among the females, centenarians, illiterate people, city residents, unmarried people. The study did not find a similar negative association in the adjusted longitudinal model.
Herr et al., 2015	SES	The socioeconomic position over the life course can be measured through indicators such as family standard of living in early life, number of years of education in young adulthood, occupation status in midlife, and perceived financial security in old age.	The critical period, accumulation, and sensitive period perspectives propose that the harmful effects of social adversity on health operate through environmental, behavioral, and physiological pathways. Education shapes knowledge, skills, attitudes, and behaviors, thereby influencing occupational class, which in turn determines economic resources and exposure to physical and psychosocial hazards across adulthood. In later life, limited finances can restrict access to health care and supportive resources, even in systems with broad social coverage, whereas adequate financial means allow older adults to compensate for	The sensitive periods model, childhood and young adulthood, was the model that best fitted the data, indicating that it is best to consider<br>the specific effect of each period of social disadvantage in a model including indicators at each stage of life rather than consider accumulation of periods of social disadvantage or their critical effects during given periods.

			functional limitations by paying for home adaptations and in-home care services.	
Hoogendijk et al., 2016	Social environment	Social functioning was assessed at T1 (baseline) and T2, focusing on social network size, instrumental support, emotional support, and loneliness. The personal network size was measured using the domain-specific network delineation method, where respondents identified important contacts. For the nine most frequently contacted individuals, information on instrumental and emotional support intensity was collected (0–36), with higher scores indicating more support. Loneliness was evaluated using the De Jong Gierveld loneliness scale, scoring from 0 to 11, where higher scores reflect greater loneliness.		Cross-sectional linear regression showed that pre-frail and frail older adults had a smaller social network size and higher levels of loneliness compared to their non-frail peers. In the Longitudinal model only the loneliness remained significant.
Hsu & Chang, 2015	Social environment	Financial satisfaction: Subjectively measured as satisfaction with their economic status.<br>Having social participation: The individuals who had work or participated in community activities or engaged in voluntary work.	Education, having higher financial satisfaction, and social participation are protective factors to frailty. Education and financial satisfaction often reflect ones socioeconomic status, and thus ones subjective level of financial satisfaction is likely to indirectly predict one's subjective well-being.	The first group, with a stable and low frailty risk, comprised 43.5% of participants. The second, called the developing frailty group, had a prevalence of 38.8%. The third group, the high-risk frailty group, showed consistently high frailty risk from baseline to study end, with a prevalence of 17.7%. Higher financial satisfaction protected the developing frailty group from worsening frailty, while social participation was protective for the high-risk group. These findings highlight the importance of financial and social factors in frailty trajectories among older adults.
Hu et al., 2020	Physical environment	Air quality index: Air quality data from the Ministry of Ecology and Environment of China provided for each city for each day for the period from January 1, 2000, to December 31, 2014.		All models show increased frailty with higher exposure to air pollution in one year prior to the interview, when controlling for short-term fluctuations. Moreover, elderly people living in areas where air pollution increased over the follow-up had larger increases in frailty scores than those where air pollution was relatively constant.
Hu et al., 2022	Social environment	Chinese shortened social capital scale: The structural social capital with 11 items, comprising three dimensions: social participation, social support, and social connection; The cognitive social capital with 11 items, comprising three dimensions: trust, cohesion, and reciprocity.	Social cohesion results from a complex process involving factors such as neighborhood quality, individuals' mental and physical health, their subjective assessment of the community, and the nature and quality of social contacts. Cognitive social capital influences health by controlling risky behaviors, reducing stress, and providing mutual assistance and support. This mediating effect can be divided into two stages: first, cognitive social capital positively impacts health-promoting lifestyles; second, these lifestyles negatively affect frailty. However, individuals with higher structural social capital may still struggle to benefit their health if they lack good cognition and experience of the social environment.	Cognitive and structural social capital showed a moderate positive correlation, indicating that individuals with richer participation in social structures also tend to report higher perceived trust, reciprocity, and support. Cognitive social capital was significantly and inversely associated with overall frailty and all three of its dimensions, whereas structural social capital showed no significant association after adjustment for other covariates. Moreover, health-promoting lifestyles partially mediated the relationship between cognitive social capital and frailty, suggesting that higher perceived trust and support may reduce frailty risk in part by encouraging more beneficial health behaviors.
Huang et al., 2024	SES	Life-course socioeconomic position (SEP) was captured using several indicators: age at completion of full-time education, whether one's main job involved heavy manual or physical work, whether the job required mainly walking or standing, and average total household income before tax.	Jobs involving heavy manual labor or long periods of walking and standing can overburden the musculoskeletal system, leading to chronic pain, joint damage, and functional decline that increase frailty risk. At the same time, chronic low-grade inflammation is strongly associated with frailty, linking physical stressors to accelerated physiological aging and greater vulnerability to adverse health outcomes.	The study found strong associations of genetically predicted jobs involve heavy manual or physical work and job involves mainly walking or standing with frailty.
Ikeda et al., 2019	SES	Individual's current SES: educational attainment, household income.	A lower SES is clearly associated with poor health-related behaviors, including physical inactivity, smoking, obesity, limited use of health care services and poor social activities.	Highly educated older adults were found to have a higher likelihood of recovering from pre-frailty. However, for older individuals who were frail at baseline, there were no observed

			This negative conduct with regard to well-being may contribute to socio-economic inequalities in both deterioration and improvement from the state of frailty.	differences in recovery to either robustness or pre-frailty based on educational attainment. Participants with higher incomes were more likely to recover from pre-frailty. Older individuals with higher levels of education and income were less likely to transition from robustness to pre-frailty, frailty, disability, or death.
Iriarte et al., 2024	social environment	The Area Deprivation Index (ADI) is constructed from 17 socioeconomic indicators, including income, education, employment, and housing quality, sourced from the US Census 2015. This index enables the ranking of neighborhoods based on socioeconomic disadvantage within a specific region and is calculated for block groups corresponding to participants' home addresses.	The ADI is rooted in four factors: income, housing, employment, and education. These modifiable factors may interact to increase or decrease the chances of harmful outcomes. For instance, neighborhood disadvantages such as walkability and safety may determine physical activity levels and social engagement and, therefore, mediate the pathway between frailty and other adverse health outcomes such as falls and death.	National ADI did not differ by frailty status.
John et al., 2013	SES	Social positions can be evaluated based on indicators such as education level (measured by the number of years in school), income insecurity (reflecting how well income and assets meet needs), household income (average monthly income for the household), home ownership status (whether one owns, rents, or lives with family), and satisfaction with income (subjective assessment of contentment with financial situation).		All indicators of social position showed a graded, rather than threshold, relationship with frailty, with lower status consistently associated with higher frailty levels. A ceiling effect was observed for education, such that participants with more than 12 years of schooling had frailty rates similar to those with 10–12 years of education. In addition, home ownership was protective; older adults who owned their homes were less likely to be frail than those in other housing situations.
Keränen et al., 2017	Technology	Internet use during the past three months was recorded, along with the devices used to access it. Technology acceptance was captured by three constructs: performance expectancy (perceived benefits of using technology), effort expectancy (perceived ease of use), and social influence (perceived pressure from important others to use technology).	Differences in ICT adoption between frail and nonfrail older adults are unlikely to be driven mainly by physical or economic constraints, as neither visual impairment nor perceived cost remained significant in multivariable analyses. Instead, ICT use, whether for direct social interaction or for other tasks, has been linked to better well-being, higher quality of life, and greater social engagement in later life. ICT activities also provide cognitively and intellectually stimulating challenges that can enhance feelings of empowerment, while computers and the internet support autonomy by enabling older adults to complete many routine tasks via electronic services.	The most common forms of internet use involved e-services (for example, online banking, social services, tax services, and ticket purchases), information seeking (such as timetables, health information, and recipes), news, and communication (for example, email or Skype). Nonusers tended to hold more negative views regarding performance expectancy, interest, effort required, social influence, and subjective cost. Among nonusers, those who were frail had particularly unfavorable attitudes, expressing more negative opinions than non-frail nonusers overall and showing especially strong resistance toward advanced mobile ICT.
Kheifets et al., 2022	SES	Individual and neighborhood SES as composite score self-report and included years of education, family status, current employment status, house-hold income, neighborhood SES based on home address via an index developed and validated by the Israel Central Bureau of Statistics.		Education and income, as well as area-based SES, serve as strong predictive factors for physical activity frequency, and subsequent development of frailty.
Kim et al., 2019	Physical environment	Perceived neighborhood walkability: pedestrian friendliness, comfort, safety, convenience.<br>Perceived neighborhood environmental pollution: water pollution, noise pollution, temperature, excessive sunlight.	Walkable neighborhoods with low pollution encourage physical activity by enabling residents to walk rather than use other transport. Poor land use, disconnected sidewalks, and heavy traffic reduce daily activities. Residents in walkable areas socialize more, gaining community support through easier neighbor interactions and walking companionship, enhancing social connectivity. In this study, most subjects grew cash crops in greenhouses, involving uncomfortable postures and intensive labor, leading to more musculoskeletal issues and lower health-related quality of life. These factors	Frailty was associated with low perceived neighborhood walkability, and severe perceived neighborhood environmental pollution after adjusting for sex, age, cohabitation status, educational attainment, employment status, monthly income, grip strength, and polypharmacy.

			highlight the need for careful interpretation of rural study outcomes due to environmental and occupational variations.	
King et al., 2017	Social environment	Structural neighborhood disadvantages were measured by the percentages of Black residents, families with income below the poverty level, households receiving public assistance, female-headed households, residents under age 18, and unemployed male civilians aged 16 years and older.	While neighborhood disadvantage may have a significant effect for some matters and for some people, it may have an attenuated effect when needed resources are provided and used at a broader geographic level, and when access is facilitated.	Neighborhood disadvantage significantly predicted higher frailty in unadjusted analyses, but this association was fully explained by race.
Kwan et al., 2019	Social environment; Physical environment	Life-space was measured as an independent variable using the Life-Space Assessment with five specific levels by proximity from inside the home to outside a district. Social participation was measured by the Reintegration to Normal Living Index (RNLI). The RNLI measures contain 11items categorized into two factors: physical and social.	Life space is preferentially and negatively associated with slowness. Reduced life-space may result from a poor age-supportive environment that does not offer convenient access to resources or recreational facilities, social support, transportation, neighborhood security, and a user-friendly walking environment. Frail older people who walk slowly may find it difficult to navigate in a city. Thus, helping slow-walking older people to maximize their life-space through environmental design. The perceived satisfaction with social participation is associated with lower frailty. This implies that the perception of satisfaction with social participation may be more important than how much a person actually participated in the social events.	The results showed that social participation and life-space are negatively associated with frailty.
Kwan et al., 2023	Technology	Technology acceptance was measured by the Senior Technology Acceptance Model-14 item which consists of four constructs: attitudinal beliefs, control beliefs, geotechnology, anxiety, and health.	Under the COVID-19 pandemic, because of quarantine and social distancing measures, the level of participation in physical activity of community-dwelling older adults was reported to decline, albeit the fact that they expressed the need to remain physically active. One possible explanation is that people with higher levels of technology acceptance may use technology more to engage in more physical activity during COVID-19.	This study showed that physical activity and technology acceptance were associated with frailty, and technology acceptance moderated the association of physical activity with frailty.
Lang et al., 2009	Social environment	Structural Neighborhood deprivation: Index of Multiple Deprivation with seven dimensions included: income deprivation; employment deprivation; health deprivation; education, skills, and training deprivation; barriers to housing and services; living environment deprivation; and crime. Personal wealth: Total wealth was assessed using a range of questions in which participants were asked about their financial and housing wealth and their other assets.		Frailty in older adults is independently associated with individual and neighborhood socioeconomic factors. Older adults who are poor and live in deprived neighborhoods are most vulnerable. Subgroup analysis reported no difference to the direction of the relationship between FI. and wealth or neighborhood deprivation.
Lee et al., 2020	Physical environment	Daily PM2.5 level	Putative mechanisms explaining the health consequences of PM2.5 exposure include oxidative stress, inflammatory responses, and gene/epigenetic modification factors also implicated in aging and frailty pathogenesis. Airborne pollutants may disrupt homeostasis and accelerate age-related declines in functional performance and capacity at cellular, organ and system levels; multi-systemic homeostatic disruption would conduce frailty.	The associations were stronger among men, people older than 75 years, with higher disease burden, non-smokers, and those with lower household income.
Li et al., 2020	SES	Childhood neighborhood quality before age 17 was assessed by four questions asking the participants whether the neighborhood they lived as a child was safe, willing to help, close knit and clean and attractive.	Neighborhood quality during childhood can impact health outcomes both directly, such as through exposure to dangerous physical environments, and indirectly through factors like social connections, social support, lifestyle choices, and access to health and social services. Social cohesion, as indicated by dimensions like willingness to help and close-knit communities in the study, reflects the density of social	Older adults who experienced low socioeconomic status or poor health in childhood are more vulnerable to frailty in later life, even after accounting for adult circumstances.

			networks, the structure of social relationships, and individual involvement in society.	
Li et al., 2021	Physical environment	Rural areas are characterized by agricultural production and low population density, whereas urban areas are dominated by industrial, commercial, and other non-agricultural activities and have higher population density. Rural–urban residence was classified using a neighborhood-level item asked of local officials: “Is this a community office or a village office?” Neighborhood socioeconomic status was proxied by per-capita net income, and data on the availability of neighborhood aging services were also collected.	The Neighborhood Stress Process Model suggests rurality and community socioeconomic characteristics influence mental well-being by moderating the link between physical and mental health. It’s not community traits directly but their moderating effect that explains rural-urban differences. In rural China, limited healthcare availability and affordability increase vulnerability to stress’s negative effects. Cultural factors also play a role: older adults needing care may see themselves as burdens, and families must pool scarce resources. These dynamics highlight how rural and urban contexts differently shape the mental health impact of physical health decline.	A stronger relationship between deterioration in frailty and depressive symptoms in rural neighborhoods and neighborhoods with lower SES, after controlling for individual-level SES. Also, the moderating effects of the neighborhood-level socioeconomic factors remained after controlling for urbanicity, indicating that neighborhood SES works beyond the rural-urban contexts.
Li, 2024	Technology	Internet use was measured with the following items: HRS: ‘Do you regularly use the Internet (or the World Wide Web) for sending and receiving e-mail or for any other purpose, such as making purchases, searching for information, or making travel reservations?’; CHARLS: ‘Did you use the Internet in the past month?’; SHARE: ‘In the last seven days, have you used the Internet at least once for e-mailing, searching for information, making purchases, or for any other purpose?’; and MHAS: ‘Can you access Internet service at home?’.		Internet use was consistently linked to a lower risk of being frail. Social isolation partly explained this relationship, suggesting that going online helps reduce frailty in part by keeping people more socially connected. The protective association was somewhat stronger among older adults who were at least in their mid-sixties, men, those not in paid work, those not married or partnered, non-smokers, people who drank alcohol, and those not living with their children.
Liu et al., 2020	SES	Health literacy: The health literacy scale was constructed by referring to the Chinese Citizen Health Literacy Questionnaire, with 33 items covering three dimensions: knowledge and belief literacy, behavior literacy, and skill literacy. Social support: Social support was measured using the Social Support Rating Scale, with three items measuring objective support and four items measuring subjective support.	Depression commonly manifests as cognitive impairment, with insufficient coping resources in psychological and social areas, and older adults can lose interest in physical and social activities, elevating the risk of decreased physical function and falls. Participants with a lower education level were inclined to have lower health literacy, probably due to their limited ability to acquire health information and poor acceptance of knowledge.	Health literacy had an indirect negative association with frailty through the mediating effect of social support and depression.
Liu et al., 2022	Physical environment	The neighborhood environment comprises both physical and social resources that support residents’ emotional and physical well-being. It includes physical elements such as basic infrastructure, transportation, passable roads, accessibility, and health care facilities, as well as social features like community organization, area-level socioeconomic conditions, access to social support, opportunities for social and leisure activities, and exercise facilities.	Better infrastructure like sewer systems and paved roads provides rural older adults convenience and smooth travel. Accessible neighborhood exercise facilities are linked to lower baseline frailty and slower frailty progression. More voluntary social organizations encourage participation, reducing loneliness and improving health. Rural older adults rely more on informal care due to fewer personal resources and family migration to cities. Volunteering helps fill gaps left by absent family and limited public eldercare. While infrastructure impacts frailty in both rural and urban areas, poor infrastructure significantly accelerates frailty only in rural populations, reflecting socioeconomic disparities.	Accessible exercise facilities were associated with a lower initial level of frailty only among rural older adults. Over the 7-year follow-up period, better basic infrastructures and accessible exercise facilities were associated with a slower increase rate of frailty scores among rural residents. Higher community-level socioeconomic status was associated with a lower initial level of frailty only among urban older adult.
Liu et al., 2023	SES ; Social environment; Physical environment	life course SES and community basic infrastructure and social organization. Life-course Socioeconomic Status: never disadvantaged; three Critical Stage patterns; three Social Mobility patterns; one Cumulative Exposure pattern. Community physical and social environmental resources: community physical and social environmental resources: community basic infrastructure and Community voluntary organizations.		Individuals who experienced two or three stages of cumulative SES disadvantages not only had a higher level of frailty at baseline but also experienced a significantly faster increase in frailty over time. A very small association between childhood SES disadvantages and the initial level of frailty, suggesting that the divergence of health for community-dwelling older adults may start in adulthood. Individuals with poor SES were more likely to be constrained by the lack of basic

				infrastructure resources than individuals with better SES and were also more likely to benefit from the buffering effects of better basic infrastructure resources.
Liu et al.,2024	Physical environment	Pollution Assessment Ambient air pollutants including PM1, PM2.5, PM10, NO2, and O3.	Previous studies have established that air pollution produces adverse effects on health, primarily through mechanisms involving inflammation, oxidative stress, metabolic disturbances, and genetic and epigenetic modification.	Air quality improvement in PM1, PM2.5, PM10, and NO2 could alleviate the progression of frailty.
Lyu et al., 2022	Social environment	Social organizational participation was assessed by evaluating if participants take part in any of the organizations.<br>Social support was evaluated by the 2-Way Social Support Scale. Social networks were measured using the Lubben Social Network Scale.	Personal social networks have been indicated to be associated with physical activity and improving social networks and interpersonal processes may enhance the effectiveness of physical activity.	Frailty is closely associated with nutrition-related, physical, and social factors.
Maharani et al., 2023	SES; Physical environment	Neighborhood deprivation was assessed using the Index of Multiple Deprivation (IMD), which comprises seven domains measured at the Lower-layer Super Output Areas (LSOAs) level: income deprivation, employment deprivation, education, skills and training deprivation, health and disability deprivation, crime, barriers to housing and services, and living environment deprivation. Household wealth was measured as the net total wealth of a benefit unit, defined as the sum of savings and investments after financial debts have been subtracted.		Individuals who are less wealthy and live in more deprived areas are more likely to become prefrail or frail and have higher risks of being prefrail/frail. poorest individuals who live in the most deprived areas are at the highest risk of prefrailty and frailty. A greater effect of household wealth and neighborhood deprivation on having prefrailty or frailty amongst men than women. Living in urban areas is not associated with an increased odds of prefrailty or frailty. No significant differences appear in the links between household wealth and neighborhood deprivation with the odds of being prefrail and frail.
Maltby et al., 2020	Social environment	Social isolation was defined using five indicators: being unmarried or not cohabiting, having less than monthly contact with children, having less than monthly contact with other family members, having less than monthly contact with friends, and not belonging to any social organization. An overall isolation score was first constructed using these five indicators in line with prior research. A second set of variables applied an alternative scoring scheme to the same items to better capture the multidimensional nature of social isolation.		Social isolation emerged as a multidimensional construct with three distinct facets: isolation from the nuclear family, from other immediate relatives, and from the wider social network. Of these, isolation from the wider social network uniquely predicted a specific dimension of frailty over four years, even after controlling for baseline frailty and multiple potential confounders.
Manrique-Espinoza et al., 2016	physical environment	At the household level, an asset index was constructed with a count of 18 household assets which includes ownership of the following goods: own house, TV, radio, car, bike, stereo, computer.	The role of self-perception of health as a frailty predictor can be explained through the model, where self-appraisal has biological and cognitive underpinnings that correlate with objective or hard measures (biomarkers) of health, thus suggesting that self-appraisal captures the physiological state of individuals.	Living in households with higher socioeconomic status reduces the probability of being pre-frail or frail.
Martins et al., 2020	Physical environment	Perception of neighborhood built environment: The Neighborhood Environment Walkability Scale (NEW) consists of residential density, land-use mix diversity, land-use mix access, street connectivity, walking or cycling facilities, aesthetics, traffic safety, crime safety.	Life-space constriction theory and environmental stress theory: Perceptions could feed into the constriction of their life-space, defined as the spatial areas a person moves through his or her life. These perceptions influence physical and social activities and make it harder for the frailer person to break out of the frailty cycle of health decline. These changing perceptions of environmental demand might create maladaptive behaviors to older frail adults, being detrimental to health, as theorized by the environmental press theory.	After adjusting for age, gender, marital status, social isolation, economic situation, and physical activity, older adults with increasing FI scores were more likely to perceive their environment as having fewer destinations, worse street connectivity, poorer infrastructure for walking, poorer aesthetics and higher levels of crime than less frail older adults have.
Martins et al., 2021	Physical environment	Perceptions of neighborhood built environmental were derived from the Neighborhood Environment Walkability Scale (NEWS). The NEWS has nine subscales residential density; land use mix diversity; land use mix access; street	Negative perceptions of one's life-space can restrict both social interactions and physical activity within neighborhood spaces, as individuals may feel less motivated or safe to go out and engage with others or use local amenities.	Frail and pre-frail participants were more likely to live in areas with lower residential density and fewer road crashes, but with greater accessibility to services. Frailty and pre-frailty were significantly associated with negative perceptions of the

		connectivity; walking and cycling facilities; traffic safety; traffic load; and crime safety. Objective neighborhood environment were collected and mapped using ArcMap GIS software. Seven different area-level measures were chosen to match the existing subscales of NEWS: residential population; land use mix diversity; accessibility to services; street connectivity; persistent green cover; density of road crashes; and crime rate.		neighborhood, particularly low land-use diversity, lower perceived accessibility to services, and lower perceived safety from crime. In subscale analyses, land-use mix diversity, land-use mix access, and perceived crime safety remained significantly associated with frailty after adjustment for socio-demographic variables.
Mena et al., 2020	Physical environment	Perceptions of the immediate environment, including recreation, transportation, neighborhood habitability, services, general environment, and community social interactions, were assessed. GIS-based spatial analysis identified hot spots and cold spots of frailty conditions among older individuals.		Most of the frail participants reside in poor or undervalued environments, while the healthier group of patients reside in more highly valued environments,<br>Spatial clustering with respect to the physical environment was mainly related to recreation and technology; while only a few variables associated with transport, neighborhood and environment were clustered.
Mendonca et al., 2020	SES	Socioeconomic status, full-time education, and participants' postcodes were used to derive the Index of Multiple Deprivation.	A likely explanation for the weak association with SES is survivor bias. By age 85, many individuals in poorer health and from lower socioeconomic backgrounds may already have died, leaving a selected group of survivors. Among those who did enroll in low SES, health may be relatively robust compared with their deceased counterparts, which can attenuate SES effects that are more evident in younger-old cohorts.	SES did not appear to have an effect on the likelihood of moving from one frailty state to another at this age.
Mitsutake et al., 2021	Physical environment	Neighborhood walkability was self-rated and categorized into four levels: car-dependent, somewhat walkable, very walkable, and walker's paradise.	More walkable areas positively influenced greater physical activity among older adults.	The risk of frailty among participants who lived in somewhat walkable or very walkable areas was lower than that of participants who lived in car-dependent areas.
Mooney et al., 2018	Social environment	Chronic stress: 8 current and ongoing stressful situations lasting 12 months or longer. The situations included ongoing difficulties relating to the following: personal health, physical or emotional health in spouse or child, alcohol or drug use in family member, work, finances, housing, close relationships, and caregiving. Perceived control was measured as a latent variable using a previously validated measure consists of two subtypes, personal mastery reflects a general sense of efficacy in attaining goals and perceived constraints, which reflect a belief that outcomes are beyond one's control.	Greater levels of chronic stress were associated with lower levels of control, which in turn were associated with greater severity of frailty. These models posit that stress alone does not explain adverse health outcomes. Rather, the effects of stress on health are mediated by its adverse effects on psychological processes, which in turn could result in greater stress reactivity and cumulative physiological burden, coping strategies, over the long term, to the pathogenesis of frailty.	Chronic stress was related to baseline frailty with the effect being almost entirely indirect through perceived control. Perceived control also had a direct effect on lower baseline frailty. A significant indirect effect of SES on baseline frailty through control and a small but significant indirect effect through both chronic stress and control. The mediating role of perceived control in the chronic stress and frailty relationship was generally equivalent across age, gender, and racial, ethnic subgroups.
Mori et al., 2022	Physical environment	Built environments—human-made settings such as neighborhoods, parks, and streets—have attracted growing attention because of their associations with health. They can be characterized along eight features, capturing four negative and four positive aspects. Negative aspects include locations with graffiti or garbage, roads or intersections with a high risk of traffic accidents, places that feel unsafe for walking alone at night, and routes that are difficult to walk due to hills or steps. Positive aspects include access to parks and sidewalks, attractive views or buildings, access to shops selling fresh food, and houses or facilities where people feel free to drop in. These features are typically assessed by asking, "How many of the following places are there within walking distance, roughly within 1 km of your house?"	Even though individuals who are aware of hills and steps in their neighborhoods might be avoiding going out, the presence of those hills and steps at the community level unconsciously suppresses frailty onset because of the moderate resistance exercise required to get around on foot. A rich built environment might naturally have a preventive effect on frailty even without the individua's' awareness.	Individual-level negative built environment features associated with higher frailty risk included locations with graffiti or garbage, roads or intersections perceived as traffic hazards, areas considered unsafe for walking alone at night, and places difficult to walk because of hills or steps. In contrast, individual-level features linked to lower frailty risk were access to parks and sidewalks, attractive views or buildings, nearby fresh food stores, and homes or facilities where older adults felt comfortable dropping in. At the community level, living in areas with a modestly higher proportion of locations difficult to walk (e.g., hills or steps) was paradoxically associated with a slightly lower 3-year frailty risk, possibly because those who perceive walking difficulty in their neighborhood are more likely to limit outings.

Mori et al., 2023	Physical environment	Places are there within walking distance, roughly within 1 km, of one's house. Five subjective built environment features: locations difficult for walking, hills or steps, access to parks and sidewalks, fascinating views or buildings, access to fresh food stores, houses or facilities where you feel free to drop in.	Access to parks and sidewalks, access to fresh food stores, houses or facilities where people feel free to drop in, and fascinating views or buildings contributed to reduced onset of frailty. Walking time, depression, and social support were mediating factors in the relationship between built environment and the onset of frailty. Highly walkable environment can reduce the onset of frailty, including by increasing individuals' amount of walking and physical activity.	Four built environmental features contributed to reducing the onset of frailty: access to parks and sidewalks, access to fresh food stores, houses or facilities where people feel free to drop in, and fascinating views or buildings. Significant mediation effects were confirmed for all built features with a walking time of at least 30 minutes, depression, and social support.
Mümken et al., 2024	Social environment	Social network characteristics, assessed by network size and the presence of someone with whom respondents could discuss important personal matters. Subjective social experience was captured by social motivation and loneliness, measured using the 3-Item Loneliness Scale alongside the four-item EURO-D depression scale. Social out-of-door activity was indexed by a summed score of four social activities that typically require leaving the home.	Life-space mobility may explain the mediating role of social out-of-home activities identified in our study. Reduced participation in these activities signals early life-space constriction and diminished physiological reserves, limiting outdoor autonomy. Social out-of-home activities help maintain social contacts despite shrinking networks in older age, facilitating the exchange of functional and emotional support and health information, which may delay frailty onset. Additionally, such participation fosters a stronger sense of community belonging and social identity, which are linked to better self-rated and objective health, potentially improving frailty outcomes.	The direct effects of loneliness and lack of motivation on EFS-scores were partially mediated by performed social out-of-home activities. A significant association between increasing participation in social out-of-home activities and a reduction of frailty symptoms using a linear mixed model.
Nakajima et al., 2019	Physical environment	Perceived indoor temperature was examined using the standardized questionnaire, Comprehensive Assessment System for Built Environment Efficiency Health Checklist. Economic satisfaction was measured with a self-reported question with four response choices.	One possibility was that people who are unsatisfied with their economic situation were the people having difficulty warming up their house and at risk of fuel poverty.	An interaction effect showed that people in the Cold Group and unsatisfied with their economic status had significantly higher frailty index scores.
Nishimura et al., 2024	Physical environment	Urban–rural status was defined using criteria from Japan's Ministry of Internal Affairs and Communications: an urban area was a central city with a population exceeding 1 million and the surrounding municipalities whose residents primarily commuted to that city for work or school.	Many participants in rural areas did not use public transportation or walk for >15 min. This indicates that cars are their primary means of transportation and that they have few opportunities to walk unless they are aware of them.	The prevalence of frailty, with robustness being particularly significantly lower in rural areas than in urban areas 29.7% vs. 43.9%. Living in rural areas was associated with frailty. after adjusting for age, gender, HbA1c levels, and diabetes duration.
Noguchi & Shang, 2024	Social environment	Individual social capital was conceptualized along two dimensions: a cognitive dimension, reflecting perceptions of community social cohesion, and a structural dimension, capturing informal socializing and participation in social activities.	Rich substantive connections with neighbors and friends are closely linked to acquiring informational and instrumental social support, which may help frail older adults live in the community independently, through supporting essential tasks such as shopping at stores, bank procedures, and using transportation services. However, the cognitive aspects of individual social capital did not have any moderating effects on functional ability decline. Although trust and perceived reciprocity in the community have been associated with improved well-being and mental health, its effects may be modest in terms of the practical benefits of maintaining living functions.	Structural dimension of social capital moderate frailty against functional ability.
Noguchi et al., 2022	Social environment	Community-level social capital was conceptualized at both structural and cognitive levels. At the structural level, civic participation was measured as the percentage of residents in each community who took part in volunteer, sports, hobby, study/cultural, or skill-teaching groups. At the cognitive level, social cohesion captured general trust, perceived willingness of others to help, and attachment to the residential area, while reciprocity reflected emotional support received and provided, as well as instrumental support received.	Group-level social capital affects health through mechanisms like social contagion, spreading health information and behaviors; informal social control, managing unhealthy behaviors; and collective efficacy, mobilizing groups to develop health-related facilities. Rich civic participation connects diverse individuals and bridges social classes. However, as physical decline is more pronounced in old-old adults than young-old adults, community-level civic participation alone may be insufficient. For old-old adults,	Higher community civic participation demonstrated a significant association with a lower risk for frailty onset.

			individual interventions alongside fostering community social capital are likely necessary to address their specific health needs effectively.	
Nyende et al., 2023	Social environment			A perceived sense of control can be converted into personal and social resources that help older adults manage daily life and adapt to age-related challenges. Three main themes emerged: control expressed through bodily experiences and everyday activities, control shaped by place of residence, and control negotiated within health and social care relationships. Living at home, in particular, was associated with a stronger sense of safety, independence, and continuity for older people with frailty.
Ocampo-Chaparro et al., 2019	SES	Socioeconomic, cultural and environmental conditions factors. Social affiliation: Access to recent problems to health services.		The factors significantly associated with frailty were older age, being women, living in rural areas, having low education, a greater number of medical conditions, insufficient current income, childhood health problems and a poor economic situation in childhood.
Peek et al., 2012	Social environment	Perceived social support was measured as emotional support using a two-item scale asking whether, in times of trouble, respondents could count on at least some family or friends and whether they could talk about their deepest problems with at least some of them. Stressors were operationalized as recent adversities, including financial strain and the occurrence of negative health events or other unfavorable life events in the previous 12 months.	Financial stressors had consistent deleterious effects on frailty. Having lower socioeconomic position is associated with increased exposure to social, psychological, and physical risk factors related to poor health outcomes, along with having fewer resources to deal with negative circumstances	Social support protects against increases in frailty for the group that is characterized by progressive moderate frailty. The stable low frailty group and the progressive high frailty groups are less influenced by potentially modifiable characteristics, such as social support. The middle group potentially represents a group in transition, not quite as high risk at the progressive high frailty group nor as stable as the least frail group.
Pérez-Hernández et al., 2018	Physical environment	Living in an apartment building with elevator, having piped hot water, a heating system, feeling cold frequently at home, having a bathtub or shower, a refrigerator, a washing machine, a landline, and a room of your own are all aspects of housing conditions.	Living in walk-up apartments may prevent older adults from going outdoors an activity that has been prospectively associated with functional and intellectual health benefits among this subpopulation. Cold homes have a harmful effect on health, especially among older people, who have reduced thermoregulatory control and thermal discrimination.	After multivariate adjustment, participants who lived in homes with more than poor condition showed a higher risk of frailty.
Pollak et al., 2024	Social environment	Social connection was captured at three levels: social networks as a structural indicator, social activity and social support as functional indicators, and loneliness as a qualitative indicator of connection. Perceived social support was measured with four items from the Multidimensional Scale of Perceived Social Support, and social network size was defined as the number of community members, family, and friends seen at least once per month. Loneliness was assessed using a modified 5-item De Jong Gierveld Loneliness Scale, and subjective socioeconomic status with the MacArthur Scale of Subjective Social Status.	The stress-buffering hypothesis is often invoked to explain how social connection can mitigate adverse health outcomes: people with greater material and emotional support tend to experience better health than those with less support. In this framework, loneliness—particularly when combined with low social activity and older age—emerges as a key pathway to frailty, as it increases the risk of worsening depression and impedes recovery from depressive episodes. Depression, in turn, has been proposed as an important mechanism linking loneliness to higher morbidity and mortality.	Loneliness, low social activity, and older age lead to frailty. When frailty was present, this combination of factors was present 75% of the time. Additionally, 82% of frailty in this sample was explained by this combination of conditions.
Portegijs et al., 2016	Physical environment	Life-space mobility was assessed across hierarchical levels (bedroom, other rooms, outside the home, neighborhood, town, and beyond), with participants reporting how many days per week they reached each level and whether they required personal assistance or assistive devices. Perceived autonomy in outdoor participation was measured by asking participants to rate their perceived opportunities to visit relatives and friends, make trips and travel, spend leisure time, meet other people, and live life in the way they prefer.		Life-space mobility was lower among those with frailty and pre-frailty compared with those without frailty and, in addition, declined at a faster pace. Perceived autonomy in outdoor participation was more restricted among those with frailty and pre-frailty compared with those without frailty, but the rate of decline did not differ.

Risbridger et al., 2022	Social environment	Social participation was determined by employment status, social network, and community activity.		Social isolation, and non-engagement in community activities were associated with increased frailty. Being involved in the community and maintaining social ties will provide a support system for older people, improve their mental wellbeing, and allow them to stay active; having objectives and a purpose in life has been shown to improve happiness and health.
Schwartz et al., 2023	Physical environment	Residential addresses were geocoded and linked to neighborhood boundaries defined using U.S. Census geography (for example, census tracts or block groups). Publicly available neighborhood indicators were then attached, including access to exercise opportunities, availability of healthy food options, neighborhood socioeconomic status, and rural versus urban classification. Principal component analysis was used to extract a single underlying component from these four indicators, yielding an overall neighborhood index summarizing the shared variance across these characteristics.		Participants had increased odds of being pre-frail/frail if they lived in 'resource-poor' neighborhoods as opposed to 'resource-rich' neighborhoods after adjusting for other pre-frailty or frailty risk factors. Individual health behaviors and socioeconomic factors did attenuate the association between neighborhood score and pre-frailty or frailty. The results indicate potential mitigation of a neighborhood's effect on pre-frailty/frailty by these individual-level variables.
Seo et al., 2021	Physical environment	Neighborhood environments, as assessed by a structured questionnaire, were associated with physical frailty, taking into account urban-rural disparities in these environments. The perceived neighborhood environment scale consisted of 17 self-report questions, with components including residential density (1 item), access to destinations (5 items), neighborhood infrastructure (4 items), neighborhood safety (4 items), social environment (1 item), aesthetic qualities (1 item), and street connectivity (1 item).	The presence of destination means that the environment has many places to visit like banks, medical institutes, and public facilities that provide opportunities for social and physical activities. Therefore, the presence of destinations may also increase the chances of increasing connectivity and interactions among members of society. Crime safety at night may increase stress and isolation, and lead to inactivity39 and poor mental health.	In the urban areas, absence of destination and no crime safety at night was significantly associated with physical frailty. In the rural areas, poor access to recreational facilities and no aesthetics was significantly associated with physical frailty after adjusting for potentially confounding variables.
Shah et al., 2021	Physical environment	satisfaction with the home living environment	A living environment that is not age-friendly could lead to limited physical activity and reduced opportunities, thus contributing to loneliness and isolation.	Older people who were satisfied with their living environment had lower frailty scores in both old age homes and the community.
Shakya et al., 2024	SES; Social environment	loneliness (the UCLA Loneliness Scale); Everyday discrimination, being treated with less courtesy or respect than other people, receiving poor service than others at restaurants or stores, being treated as not smart, people acting afraid, and being threatened or harassed; Financial strain, based on the assessment of the hardship of paying monthly bills traumatic life events; low neighbor cohesion, perceptions of (a) being part of the local area, (b) trust among the people in the neighborhood, (c) friendliness among people, and (d) safety while walking alone; lower subjective social status, the MacArthur Scale was used to assess subjective social status using a 10-point score. The MacArthur Scale is a visual scale with a ten-rung ladder.	Chronic stress due to loneliness is posited to instigate hypothalamic pituitary adrenocortical pathways and inflammation, compromising immunity. Low subjective social status may contribute to chronic stress. financial strain was associated with greater odds of frailty in older adults. Financial strain may increase frailty risk via suboptimal access to nutritious food and related malnutrition. Low neighborhood cohesion may contribute to frailty in older adults through restricted participation in physical activities and psychological distress.	Psychosocial stressors: High loneliness, low subjective social status, high financial strain, and low neighborhood cohesion independently increased the odds of frailty. The mediating pathway of structural social determinants of health was not significant.
Shiau et al., 2023	SES	The socioeconomic position was evaluated across various life stages. In childhood, it is determined by the father's education and occupation. During young adulthood, it is influenced by the individual's education attainment. In the active professional life stage, socioeconomic position is shaped by the participant's education and occupation indicators. Finally, in older age, income becomes a key factor in assessing socioeconomic status.	Life-span socioeconomic position trajectories: childhood, young adulthood, active professional life, and older age all seem to be critical periods associated with frailty, although a low SEP in childhood may be more strongly associated with low physical activity in older age. a low SEP in childhood might be more strongly associated with low physical activity in older age.	Compared with the stable low SEP trajectory group, the stable high SEP trajectory group was significantly associated with a lower prevalence of frailty. The study found general evidence that low SEP at any time in the life course was associated with higher prevalence of frailty and pre-frailty.
Shin & Choi, 2021	Physical environment	The average hourly concentrations of PM2.5, PM10, and O3.	Air pollution is known to have an adverse health effect by inducing inflammation, oxidative stress, metabolic disorders, and genetic and epigenetic alterations. Exposure to pollutants	Each 1-unit increase of PM2.5 and PM10 increased the odds ratios of the frail group compared to the robust group.

			can potentiate the age-related decline and deterioration of functional properties at the cellular, tissue, and organ level.	
Shiratsuchi et al., 2024	Technology	Technology usage was operationalized as competence in several everyday digital tasks: using a mobile phone, using an ATM, operating a video recorder, and sending e-mail.	Higher ICT use is associated with the absence of frailty in individuals 75 years and older. The maintenance of ICT use has been reported to prevent cognitive decline.	Higher ICT use was not associated with frailty after adjusting for covariates. Similar associations were found in the sub-groups of women, participations with less than 12 years of education; living alone; living together. No association existed between using ICT and frailty in the sub-groups of men and less than 13 years of education.
Song et al., 2023	Physical environment	Biomass cooking fuels included straw, firewood, and charcoal, while clean fuels comprised gas, solar energy, and electricity.	The mechanisms linking biomass fuel use to frailty are not yet fully understood but are thought to be closely related to exposure to fine particulate matter and other toxic emissions from incomplete combustion. Burning biomass in inefficient stoves generates high levels of harmful gases and particles that contribute to household air pollution, with estimates suggesting that 10–38% of the carbon in solid fuels is released into the air during incomplete combustion. These pollutants can trigger chronic inflammation, oxidative stress, and multi-system physiological damage, which over time may accelerate the development of frailty in older adults.	Compared to clean fuels, cooking with biomass fuels was intricately linked to a rise in frailty risk.
Su et al., 2024	Social environment	Social isolation: self-reported family social isolation and friends social isolation.		Before the COVID-19 pandemic, greater social connectedness with friends was associated with lower frailty only indirectly, via reduced depressive symptoms, whereas social connectedness with family or relatives showed no significant indirect or direct association with frailty. During the pandemic, both friend and family connectedness became linked to frailty through their effects on depression, and family connectedness additionally showed a direct protective association with frailty.
Szanton et al., 2010	SES	Individual SES: SES was measured using education and household income.	SES has been linked to inflammation, decreased serotonin and altered biological risk profiles.	After adjustment for other factors, older women with less than a high school education had about three times higher odds of frailty than those with more schooling, and those with annual incomes below $10,000 had roughly twice the odds of frailty compared with wealthier peers.
Takatori et al., 2021	Social environment	Social activities: Exercise-based activities refer to regular participation in gymnastics classes, ground golf, Japanese croquet, and other activities engaged in by local residents.	Older people who reversed frailty progression have strong relations with the neighborhood, which may have improved their physical functions and psychological aspects.	The results of the study indicate that high self-rated health and participation in exercise-based social activity are independent factors that influence reversing frailty progression.
Thinuan et al., 2020	Social environment ; Physical environment			As physical capacities decline, previously suitable living spaces can become constraining and make everyday mobility and physical activity more difficult. Older adults respond by exerting control over their environments, for example by avoiding certain places or limiting specific activities. Beliefs about the ability and value of being physically active (self-efficacy) are critical for sustaining activity despite frailty and environmental barriers; higher self-efficacy is consistently linked to greater physical activity across cultures and age groups, even when studies do not focus specifically on frailty. Family members may interpret functional decline as a signal to restrict older adults' activity, which can further limit movement; in Thai contexts, this is partly rooted in reciprocal caregiving norms in which adult children are expected to "repay" parents through protection and care. Safety is a result

				of the interaction between physical space and physical ability that change over time as the participants age.
Tsubota-Utsugi et al., 2018	Social environment ; Physical environment	The physical environment variables include degree of housing damage categories such as completely destroyed, large-scale partially destroyed, moderate- to small-scale partially destroyed, partly damaged, not destroyed but flooded, and not destroyed or flooded. Residential status after the disaster includes options like not displaced, temporary housing, other residence, family, friend, or relative's house, newly built house after the disaster, rental apartment, and other. In the social environment assessment, social networks were evaluated using the 6-item Lubben Social Network Scale. The scale includes questions about the number of relatives or friends seen or heard from at least once a month, the number of relatives or friends one feels close enough to call on for help, and the number of relatives or friends one feels comfortable talking with about private matters.	A sedentary lifestyle was associated with the risk of frailty among women who had experienced extensive housing damage and those living in temporary housing. No facilities or public transport services are available in the surrounding areas. Therefore, those who were living in temporary housing faced more difficulties in terms of accessing other facilities and transport. Among women forced to move a long distance from the disaster area, the difficulty of accessing existing communities and the inability to share their experience of the disaster or damage might have increased the risk of frailty. By contrast, the reason that a risk of frailty was not associated with psychological distress or a poor social network among women living in temporary housing may be explained by the fact that the residents living in temporary housing had experienced the same degree of housing damage.	Among men, psychological distress, in parallel with a poor social network, was related to frailty among only the participants with extensive housing damage and those living in temporary housing. Among women, worsening psychological distress was associated only with no damage and no displaced survivor. Among women with extensive damage and displacement, health outcomes such as overweight and diabetes and poor social networks were strongly related to frailty.
Uccheddu et al., 2019	Care/service	Countries were grouped into three welfare regimes: Northern Europe (Denmark, Sweden) as social democratic welfare states with universal social rights and high levels of de-familization; Southern Europe (Italy, Spain) as a “Southern” regime characterized by fragmented, sub-protective welfare provision and strong reliance on family support; and Western Europe (Austria, Belgium, France, Germany, Switzerland) as belonging to the Bismarckian where social insurance is closely tied to employment and status.	More generous, highly de-commodifying welfare policies in Scandinavian social democratic states appear to offer better protection against the adverse health impacts of low socioeconomic status.	The positive impact of education and wealth on health (frailty) is stronger for women living in countries where the welfare arrangements are less de-commodifying and de-familializing. No such interaction is found for income and for fixed-effects estimates.
Uno et al., 2021	Social environment	Social network scale: The LSNS-6 is a self-report six-item questionnaire that measures social engagement, including that with family and friends. One subscale evaluates family ties, while the other evaluates friendship ties.	Social isolation induces psychological distress and exhaustion. Exhaustion is strongly related to the presence of depression. The results of the present study indicate that relieving friendship-social isolation may prevent prefrailty and physical frailty.	Prefrailty was only associated with friendship-related social isolation at baseline.
Van der Linden et al., 2020	SES	Childhood socioeconomic conditions were calculated using Wahrendorf and Blane's measure of childhood circumstances. This measure combines four binary indicators of socioeconomic conditions at age 10, including the occupational position of the main breadwinner, number of books at home, overcrowding, and housing quality. Indicators of adult socioeconomic conditions (ASC): highest educational attainment (in years), occupational class (high skill and low skill), and satisfaction with household income.	This suggests that adult-life socioeconomic conditions capture the cumulative effect produced by childhood socioeconomic conditions in relation to frailty.	Results showed that lower educational attainment and having difficulty making ends meet with household income was associated with higher odds of being frail. Bismarckian welfare regime is the least able to deal with cumulative disadvantage. The frailty trajectories were similarly impacted: initial inequalities have not been absorbed, and they affect health in later life.
Veronese et al., 2023	Physical environment	Average air pollution, PM2.5, PM10, and NOx. The mean noise pollution index was obtained as the arithmetic mean of residential sound levels detected at the daytime, the evening, and night.		Air pollution was significantly associated with the presence of frailty and prefrailty independently from several confounders considered.
Wang & Hulme, 2021	SES			This review showed that the selected studies did not reach consistent conclusions about the relationship between frailty and socioeconomic status. Regarding age patterns in frailty, evidence was found for the age-as-leveler, status maintenance, and cumulative advantage hypotheses.

Wang et al., 2023	Social environment	Social capital consists of social cohesion and social interaction is assessed using two separate instruments. Social cohesion is assessed using the Neighborhood Scales, which consists of 4 items, and the social interaction scale consists of five items.	The possible mechanism may be that communicating with others can obtain emotional and social support and then buffer against physiological stress, thereby reducing the likelihood of frailty.	After adjusting for sociodemographic factors, higher social capital was associated with lower frailty and fewer depressive symptoms, whereas depressive symptoms were positively associated with frailty. Social capital predicted a reduced risk of frailty in logistic regression, with depressive symptoms partially mediating this relationship.
Wang et al., 2024	SES	Sources of income included: pension, continued employment, financial dependence on immediate family (spouse, child, or grandchild), and reliance on other people or government subsidies. Medical insurance captured access to health coverage. Community support referred to practical services such as daily and personal care, help with living tasks, shopping assistance, and home delivery. Social support reflected opportunities to spend time with others, enjoy entertainment, and participate in social activities.		Financial dependence increased the risk of low quality of life (QoL) among prefrail and frail older adults, but not among those who were robust. Having commercial or other forms of insurance and receiving community-based social support protected prefrail and frail individuals both from low QoL and from progression of prefrailty. Continuing to work was associated with lower QoL yet simultaneously acted as a protective factor against worsening prefrailty.
Wang, 2023	SES	Eleven individual ACEs, including intrafamilial aggression, family dynamics, loss or threat of loss within the family, socioeconomic status, and neighborhood quality,	The social mobility model has revealed that changes of the micro-social context over the life course will lead to variations in their health. For China, when the older people in this study were children, they grew up in a system inspired by an egalitarian ethos, with a low-level protection. Due to economic transition, they have gone through a limited and fragile welfare regime.	In general, the association between ACEs and FI exhibited that those who were least or most likely to experience adversity experience were less affected by ACEs while experiencing childhood adversities. The study suggests that childhood disadvantage is not always bad for FI, but the extent of which is shaped by social context where ACEs happen. Macro-level social factors: the association between ACEs and FI are differentiated by cross-country difference welfare regime e.g., Germanic countries vs. northern Europe.
Williams et al., 2023	Physical environment	Self- identified unmet resource needs related to transportation, housing, food, utilities, and medications/medical care.	First, unmet basic needs are likely associated with less access to medical care and reduced preventative care, thus resulting over time in increased rates of frailty. Second, insecurity of basic needs may contribute to higher rates of mental distress and stress, and in turn, this chronic stress may result in increased frailty. Lastly, the mental distress and stress related to unmet needs may increase risk behaviors related to frailty.	Having any basic unmet need was associated with 3.3- fold higher adjusted odds of frailty compared to not having a basic unmet need. The most prevalent unmet need was transportation insecurity, with many reporting missing appointments due to transportation difficulties. In addition, several participants reported material insecurities including food insecurity or difficulty obtaining medicine or health care.
Woo et al., 2005	SES ; Social environment	The first group consists of socioeconomic factors such as occupation, educational level, total monthly income, and adequacy of income. The second group consisted of lifestyle factors covering level of physical exercise, dietary alcohol and smoking habits.<br>The third group consisted of social support and network.		For men, increasing frailty was observed with non-white collar occupations, inadequate expenses, no or little exercise, abstinence from alcohol, few relatives or neighbors and no or infrequent participation in helping others. For women, little contact with relatives, rather than number of relatives, and absence of participation in community or religious activities were additional factors.
Wu et al., 2024	Physical environment	Levels of Exposure to Atmospheric Pollutants: hourly monitoring data obtained for seven air pollutants.		Long-term exposure to PM2.5 was associated with an increased risk of pre-frailty and frailty. PM10 exhibited similar associations. Elevated PM2.5 concentrations showed significant positive associations with the number of chronic disease scores, IADL score, and functional limitation status score.
Xu et al., 2022	Social environment; Physical environment	The age-friendliness of communities was assessed by the Age-friendly Community Evaluation Scale, which includes 3 items on housing, 7 items on transportation, 6 items on the built environment, 6 items on social participation, and 10 items on social inclusion and health services. Two alternative methods were used for assessment: individual-level AFC, calculated as the mean score of each individual's assessments on the corresponding scale's items; and community-level AFC,	A community's age-friendliness, especially the individual-level AFC, shows a positive association with the well-being of older adults, albeit to different extents. The multilevel analysis results of this study suggest that the stronger link between individual-level AFC, as opposed to community-level AFC, and frailty may be attributed to the diversity of individual preferences, accessibility, and availability of community resources.	A higher level of Age-friendly Community (AFC) is linked to lower odds of frailty, with individual-level perception of AFC playing a more significant role for older adults compared to community-level AFC.<br><br>In communities with a higher AFC, where activities are diverse and engaging, individuals who may not actively participate can still have opportunities to engage or observe

		estimated as the mean scale score of all respondents in the same community.		occasional events. This can strengthen their social support network and reduce feelings of loneliness.<br><br>Communities with a higher AFC are more likely to have better physical environments in terms of "Housing," "Transportation," and "Built Environment," providing safe facilities suitable for older individuals to engage in physical activities and establish sports groups.
Yan et al., 2022	SES	Adverse Childhood Experiences (ACEs) refer to adversities encountered before the age of 17, encompassing challenges within the family, friendships, and community. Childhood socioeconomic status (SES) was assessed using self-rated SES and the father's educational level as indicators. Childhood health and health care were evaluated through subjective measures like self-reported health status and objective indicators including being confined to bed or home for a month or longer, hospitalization for a month or more, receiving vaccinations, and having a usual source of care.	Positive childhood experiences could not only reduce the initial level of frailty in later life but also slow its progression. Moreover, the findings of this study add new evidence that objective indicators of childhood health and health care, rather than subjective indicators, were significantly associated with the change rate of frailty in later life.	Positive childhood experiences may not only lower baseline frailty in later life but also slow its subsequent progression. In addition, this study provides new evidence that objective indicators of childhood health and health care, rather than subjective reports, are significantly associated with the rate of frailty change in older age.
Ye et al., 2018	Social environment	Perceived neighborhood social and physical environment was captured through four dimensions: aesthetic quality, walking environment, social cohesion, and social participation.	The aesthetic quality of the neighborhood environment has been shown to affect health behaviors, such as improving fruit and vegetable consumption, which is an essential component of a healthy diet and one of the most modifiable risk factors for chronic disease. A high aesthetic quality in the neighborhood likely enhances residents' pleasant moods and is associated with higher mental well-being. Additionally, the walkability of the neighborhood correlates with various types of physical activity and chronic diseases. Furthermore, individuals who perceive their neighborhoods as more cohesive are more likely to foster positive social norms regarding healthy behaviors and social support. Neighbors who trust each other are more inclined to help promote access to services and amenities in daily life	After controlling individual covariates, aesthetic quality, walking environment, social cohesion, and social participation were inversely associated with frailty.
Young et al., 2016	SES	One's education level and Father's occupational classification	A small effect of childhood socioeconomic status, as measured by the father's occupational classification, persists across the life course. The variance in frailty is mainly genetically determined and strongly age-dependent, but it is also influenced by environmental factors, including personal health behaviors and characteristics such as marital status and education. This effect is mediated through educational attainment, underscoring the significance of education as a means to mitigate familial disadvantage.	The findings revealed that 45% of the inter-individual variation in Frailty Index (FI) was heritable, while 52% was attributed to the individual's unique environment. Multiple linear regression analysis indicated a small yet statistically significant inverse relationship between the father's occupational class and FI, with mediation through one's educational attainment and birth weight. These results suggest that frailty is influenced by both genetic and environmental factors.
Yu et al., 2018	Physical environment	Green space was quantified based on the Normalized Difference Vegetation Index.	Green space promotes physical activities, especially in males; memory span and mood, reduce air pollution.	In multivariable models, the frailty status of participants living in neighborhoods with more than 34.1% green space, the highest quartile, at baseline was more likely to improve at the 2-year follow-up than it was for those living in neighborhoods with 0 to 4.5%, the lowest quartile.
Yu et al., 2020	SES	Objective measures of SES: educational level, maximum life-time income. Subjective social status: Participants were asked to place a mark on a picture of an upright ladder with 10 rungs, with the lowest rung indicating the most undesirable and the highest rung indicating the most desirable state with	Subjective social status independently predicts frailty, capturing social position aspects beyond objective socioeconomic status. Older adults' social comparisons rely less on objective SES and more on lifetime achievements, capabilities, autonomy, and quality of family and social relationships. Gender roles influence these perceptions: men,	This study demonstrates that in a 14-year follow-up cohort of older Chinese people, there was a social gradient in the development of frailty, where lower subjective social status at baseline was associated with a higher incidence of frailty after an average of 14 years for men only.

		respect to their standing in the community; community status ladder.	expected to be agentic and avoid weakness, may emphasize their role as financial providers and status, increasing distress and dissatisfaction when subjective status is low. This distress heightens vulnerability to frailty. Women's communal roles may shape different social expectations and experiences, affecting frailty risk differently.	
Zhou et al., 2024	Physical environment	Indoor temperature: room air temperature, relative humidity, black-bulb temperature, and air velocity.	Frail elderly people exhibited resistance to low temperatures during the summer due to fear of physiological pain and disease infection, which also indicates their inadequate physiological regulation of summer environmental changes.	Subjective perception of the indoor environment differed by frailty level: more frail older adults were psychologically more sensitive to environmental conditions and tended to prefer slightly warmer rooms with lower air velocity. Frailty status also shaped cooling behavior, with clear differences in how and when devices were used. In particular, frail individuals tended to wait until outdoor temperatures were higher before turning on air conditioning, indicating a different threshold for initiating air conditioning use compared with less frail peers.
Zhu et al., 2020	Physical environment	Residential greenness was measured by calculating the Normalized Difference Vegetation Index (NDVI) in the 500 m radius around participants' residence.		Living in greener areas was associated with a lower likelihood of being frail, with those in the greenest neighborhoods showing notably reduced odds of frailty compared with those in the least green areas. This protective association of residential greenness was more pronounced for urban residents than for those living in rural settings.
Zimmermann et al., 2021	Social environment; Physical environment	Subjective walkability was assessed by asking, "How suitable is your external living environment for walking, using a wheelchair, or managing things?" Attachment to outdoor places was captured with the question, "How closely do you feel connected to your living environment?", alongside the interviewer's assessment of the home environment.	In older age, subjective perceptions of the home often reflect personal identity, and failure to form attachment to one's home over time can harm health and psychological well-being. Very old adults in poorer residential areas are more likely to be frail, suggesting that frailty may result from person–environment misfit, as functionally impaired older adults can struggle with unfavorable home environments, reducing mobility and increasing frailty risk. At the same time, high coping self-efficacy can protect against prefrailty regardless of health deficits, underscoring the importance of psychological resilience in aging.	The quality of the respondents' residential location assessed by interviewers, as well as their subjective attachment to their home environment, remained significant predictors of their frailty levels.
Note: Blank cells indicate that the original publication did not report this information or that the information overlapped with data presented in other cells.				

References

Abe, N., Ide, K., Watanabe, R., Hayashi, T., Iizuka, G., & Kondo, K. (2023). Social participation and incident disability and mortality among frail older adults: A JAGES longitudinal study. *Journal of the American Geriatrics Society 71*(6), 1881-1890. https://doi.org/10.1111/jgs.18269

Abe, T., Carver, A., & Sugiyama, T. (2021). Associations of neighborhood built and social environments with frailty among mid-to-older aged Australian adults. *Geriatrics Gerontology International*, *21*(10), 893-899. https://doi.org/10.1111/ggi.14253

Abeliansky, A. L., Erel, D., & Strulik, H. (2021). Social vulnerability and aging of elderly people in the United States. *SSM - Population Health*, *16*, 100924. https://doi.org/10.1016/j.ssmph.2021.100924

Aktuna, A., Hassoy, H., Ergin, I., & Mandiracioglu, A. (2023). Frailty prevalence and its associations with socioeconomic factors, health status, and healthcare utilization among elderly home care clients. *Journal of Public Health*, *32*(11), 2189-2197. https://doi.org/10.1007/s10389-023-01967-5

Alberdi Aramendi, A., Weakley, A., Aztiria Goenaga, A., Schmitter-Edgecombe, M., & Cook, D. J. (2018). Automatic assessment of functional health decline in older adults based on smart home data. *Journal of Biomedical Informatics*, *81*, 119-130. https://doi.org/10.1016/j.jbi.2018.03.009

Amiri, S., & Behnezhad, S. (2019). Systematic review and meta-analysis of the association between smoking and the incidence of frailty. *Neuropsychiatrie*, *33*(4), 198-206.

Anand, A., Syamala, T. S., Sk, M. K., & Bhatt, N. (2020). Understanding Frailty, Functional Health and Disability among Older Persons in India: A Decomposition Analysis of Gender and Place of Resident. *Journal of Research in Health Sciences*, *20*(3), e00484. https://doi.org/10.34172/jrhs.2020.20

Andrew, M. K. (2015). Frailty and Social Vulnerability. *Interdisciplinary Topics in Gerontology and Geriatrics*, *41*, 186-195. https://doi.org/10.1159/000381236

Andrew, M. K., & Keefe, J. M. (2014). Social vulnerability from a social ecology perspective: a cohort study of older adults from the National Population Health Survey of Canada. *BMC Geriatrics*, *14*(1), 90. https://doi.org/10.1186/1471-2318-14-90

Annear, M., Keeling, S., Wilkinson, T., Cushman, G., Gidlow, B., & Hopkins, H. (2014). Environmental influences on healthy and active ageing: A systematic review. *Ageing and Society*, *34*(4), 590-622.

Arakawa Martins, B., Taylor, D., Barrie, H., Lange, J., Sok Fun Kho, K., & Visvanathan, R. (2021). Objective and subjective measures of the neighbourhood environment: Associations with frailty levels. *Archives of Gerontology and Geriatrics*, *92*, 104257. https://doi.org/10.1016/j.archger.2020.104257

Aranda, M. P., Ray, L. A., Snih, S. A., Ottenbacher, K. J., & Markides, K. S. (2011). The protective effect of neighborhood composition on increasing frailty among older Mexican Americans: a barrio advantage? *Journal of Aging and Health*, *23*(7), 1189-1217. https://doi.org/10.1177/0898264311421961

Aravantinou-Karlatou, A., Kavasileiadou, S., Panagiotakis, S., Tziraki, C., Almegewly, W., Androulakis, E., & Kleisiaris, C. (2022). The Impact of Socioeconomic Factors and Geriatric Syndromes on Frailty among Elderly People Receiving Home-Based Healthcare: A Cross-Sectional Study. *Healthcare (Basel)*, *10*(10). https://doi.org/10.3390/healthcare10102079

Asiamah, N., Agyemang, S. M., Vieira, E. R., Khan, H. T. A., & Gasana, J. (2023). Sedentary behaviour among older adults residing in flat and hilly neighbourhoods and its association with frailty and chronic disease status. *BMC Public Health*, *23*(1), 2083. https://doi.org/10.1186/s12889-023-17029-0

Avila-Funes, J. A., Paniagua-Santos, D. L., Escobar-Rivera, V., Navarrete-Reyes, A. P., Aguilar-Navarro, S., & Amieva, H. (2016). Association between employee benefits and frailty in community-dwelling older adults. *Geriatrics Gerontology International*, *16*(5), 606-611. https://doi.org/10.1111/ggi.12523

Ayaz-Alkaya, S., & Kulakci-Altintas, H. (2024). Prevalence and predisposing factors of frailty and social inclusion among older adults: A cross-sectional study. *Public Health Nursing*, *41*(6), 1377-1384. https://doi.org/10.1111/phn.13411

Baker, P. S., Bodner, E. V., & Allman, R. M. (2003). Measuring life-space mobility in community-dwelling older adults. *Journal of the American Geriatrics Society 51*(11), 1610-1614. https://doi.org/10.1046/j.1532-5415.2003.51512.x

Baniassadi, A., Yu, W., Wong, A., Day, R., Travison, T., Lipsitz, L., & Manor, B. (2024). Feasibility of High-Frequency Monitoring of the Home Environment and Health in Older Adults: Proof of Concept. *Journal of Aging and Environment*, *38*(1), 18-36. https://doi.org/10.1080/26892618.2022.2131676

Baranyi, G., Welstead, M., Corley, J., Deary, I. J., Muniz-Terrera, G., Redmond, P., Shortt, N., Taylor, A. M., Ward Thompson, C., Cox, S. R., & Pearce, J. (2022). Association of Life-Course Neighborhood Deprivation With Frailty and Frailty Progression From Ages 70 to 82 Years in the Lothian Birth Cohort 1936. *American Journal of Epidemiology*, *191*(11), 1856-1866. https://doi.org/10.1093/aje/kwac134

Bigonnesse, C., & Chaudhury, H. (2022). Ageing in place processes in the neighbourhood environment: a proposed conceptual framework from a capability approach. *European Journal of Ageing*, *19*(1), 63-74. https://doi.org/10.1007/s10433-020-00599-y

Bloomfield, K., Wu, Z., Tatton, A., Calvert, C., Hikaka, J., Boyd, M., Bramley, D., & Connolly, M. J. (2024). The Association between Frailty, Quality of Life and Resilience in Community-dwelling Retirement Village Residents. *Journal of the American Medical Directors Association*, *25*(11), 105256. https://doi.org/10.1016/j.jamda.2024.105256

Caldwell, J. T., Lee, H., & Cagney, K. A. (2019). Disablement in Context: Neighborhood Characteristics and Their Association With Frailty Onset Among Older Adults. *The Journals of Gerontology: Series B*, *74*(7), e40-e49. https://doi.org/10.1093/geronb/gbx123

Cantor, M. H. (1975). Life space and the social support system of the inner city elderly of New York. *Gerontologist*, *15*(1 Pt 1), 23-27. https://doi.org/10.1093/geront/15.1_part_1.23

Cao, L., Zhai, D., Kuang, M., & Xia, Y. (2022). Indoor air pollution and frailty: A cross-sectional and follow-up study among older Chinese adults. *Environmental Research*, *204*(Pt A), 112006. https://doi.org/10.1016/j.envres.2021.112006

Casakin, H., & Reizer, A. (2016). Place Attachment and Perceived Environmental Uncertainty in Elder Adults Living in the Renewed Kibbutz. In *Environmental gerontology in Europe and Latin America: Policies and perspectives on environment and aging* (pp. 203-218). Springer.

Chaudhary, M., & Chowdhary, R. (2018). Age and socioeconomic gradients in frailty among older adults in India. *Journal of Public Health*, *27*(5), 675-685. https://doi.org/10.1007/s10389-018-0988-3

Chaudhury, H., & Oswald, F. (2019). Advancing understanding of person-environment interaction in later life: One step further. *Journal of Aging Studies*, *51*, 100821. https://doi.org/10.1016/j.jaging.2019.100821

Chen, H., Xu, X., Jia, C., Gu, H., Zhang, L., & Yi, Y. (2023). Household Polluting Fuel Use and Frailty among Older Adults in Rural China: The Moderating Role of Healthy Lifestyle Behaviors. *Healthcare (Basel)*, *11*(12). https://doi.org/10.3390/healthcare11121747

Chen, L.-J., Chen, C.-Y., Lue, B.-H., Tseng, M.-Y., & Wu, S.-C. (2014). Prevalence and associated factors of frailty among elderly people in Taiwan. *International Journal of Gerontology*, *8*(3), 114-119.

Chen, S., Honda, T., Chen, T., Narazaki, K., Haeuchi, Y., Supartini, A., & Kumagai, S. (2015). Screening for frailty phenotype with objectively-measured physical activity in a west Japanese suburban community: evidence from the Sasaguri Genkimon Study. *BMC Geriatrics*, *15*, 36. https://doi.org/10.1186/s12877-015-0037-9

Chen, Y. (2024). Childhood and adult socioeconomic status influence on late-life healthy longevity: evidence from the Chinese longitudinal healthy longevity survey. *Frontiers in Public Health*, *12*, 1352937. https://doi.org/10.3389/fpubh.2024.1352937

Chon, D., Lee, Y., Kim, J., & Lee, K. E. (2018). The Association between Frequency of Social Contact and Frailty in Older People: Korean Frailty and Aging Cohort Study (KFACS). *Journal of Korean Medical Science*, *33*(51), e332. https://doi.org/10.3346/jkms.2018.33.e332

Christensen, M. G., Jacobsen, K. K., Nilsson, C., Jepsen, R., Thygesen, L., Suetta, C., & Holm, E. A. (2024). Prevalence and population characteristics associated with frailty in a rural low socioeconomic area in Denmark: the Lolland-Falster Health Study. *BMJ Open*, *14*(3), e073754. https://doi.org/10.1136/bmjopen-2023-073754

Chu, W. M., Nishita, Y., Tange, C., Zhang, S., Furuya, K., Shimokata, H., Lee, M. C., Arai, H., & Otsuka, R. (2024). Effects of cigarette smoking and secondhand smoke exposure on physical frailty development among community-dwelling older adults in Japan: Evidence from a 10-year population-based cohort study. *Geriatrics Gerontology International*, *24*, 142-149.

Collard, R. M., Boter, H., Schoevers, R. A., & Oude Voshaar, R. C. (2012). Prevalence of frailty in community-dwelling older persons: a systematic review. *Journal of the American Geriatrics Society 60*(8), 1487-1492. https://doi.org/10.1111/j.1532-5415.2012.04054.x

Cramm, J. M., & Nieboer, A. P. (2013). Relationships between frailty, neighborhood security, social cohesion and sense of belonging among community-dwelling older people. *Geriatrics Gerontology International*, *13*(3), 759-763. https://doi.org/10.1111/j.1447-0594.2012.00967.x

Cramm, J. M., Van Dijk, H. M., & Nieboer, A. P. (2016). The creation of age-friendly environments is especially important to frail older people. *Ageing and Society*, *38*(4), 700-720. https://doi.org/10.1017/s0144686x16001240

Dai, W., Liu, S., Xu, W., Shen, Y., Yang, X., & Zhou, Q. (2024). The combined effects of heatwaves, air pollution and greenery on the risk of frailty: a national cohort study. *Scientific Reports*, *14*(1), 24293. https://doi.org/10.1038/s41598-024-73604-4

Dannefer, D. (2014). On the conceptualization of context in developmental discourse: Four meanings of context and their implications. In *Life-span development and behavior* (pp. 83-110). Psychology Press.

Dent, E., Kowal, P., & Hoogendijk, E. O. (2016). Frailty measurement in research and clinical practice: A review. *European Journal of Internal Medicine*, *31*, 3-10. https://doi.org/10.1016/j.ejim.2016.03.007

Ding, Q., Kou, C., Feng, Y., Sun, Z., Geng, X., Sun, X., Jia, T., Wang, Q., Huang, Q., Han, W., & Bai, W. (2024). Effects of air pollutants exposure on frailty risk: A systematic review and meta-analysis. *Environmental Pollution*, *361*, 124793. https://doi.org/10.1016/j.envpol.2024.124793

Duppen, D., Lambotte, D., Dury, S., Smetcoren, A. S., Pan, H., De Donder, L., & Consortium, D. S. (2020). Social Participation in the Daily Lives of Frail Older Adults: Types of Participation and Influencing Factors. *The Journals of Gerontology: Series B*, *75*(9), 2062-2071. https://doi.org/10.1093/geronb/gbz045

Duppen, D., Van der Elst, M. C. J., Dury, S., Lambotte, D., De Donder, L., & D, S. (2019). The Social Environment's Relationship With Frailty: Evidence From Existing Studies. *Journal of Applied Gerontology*, *38*(1), 3-26. https://doi.org/10.1177/0733464816688310

Dury, S., De Roeck, E., Duppen, D., Fret, B., Hoeyberghs, L., Lambotte, D., Van der Elst, M., van der Vorst, A., Schols, J., Kempen, G., Rixt Zijlstra, G. A., De Lepeleire, J., Schoenmakers, B., Kardol, T., De Witte, N., Verte, D., De Donder, L., De Deyn, P. P., Engelborghs, S., . . . Dierckx, E. (2017). Identifying frailty risk profiles of home-dwelling older people: focus on sociodemographic and socioeconomic characteristics. *Aging and Mental Health*, *21*(10), 1031-1039. https://doi.org/10.1080/13607863.2016.1193120

Espinoza, S. E., & Hazuda, H. P. (2015). Frailty prevalence and neighborhood residence in older Mexican Americans: the San Antonio longitudinal study of aging. *Journal of the American Geriatrics Society 63*(1), 106-111. https://doi.org/10.1111/jgs.13202

Etman, A., Kamphuis, C. B., Prins, R. G., Burdorf, A., Pierik, F. H., & van Lenthe, F. J. (2014). Characteristics of residential areas and transportational walking among frail and non-frail Dutch elderly: does the size of the area matter? *International Journal of Health Geographics*, *13*, 7. https://doi.org/10.1186/1476-072X-13-7

Fang, B., Huang, J., Zhao, X., Liu, H., Chen, B., & Zhang, Q. (2022a). Concurrent and lagged associations of social participation and frailty among older adults. *Health & Social Care in the Community*, *30*(6), e4812-e4820. https://doi.org/10.1111/hsc.13888

Fang, J., Ren, J., Ren, L., Qiu, X., Yuan, S., Wang, W., & Wang, J. (2022b). Perceived Social Support and Associated Factors Among Community-Dwelling Older Adults With Frailty and Pre-frailty in Hangzhou, China. *Frontiers in Psychiatry*, *13*, 944293. https://doi.org/10.3389/fpsyt.2022.944293

Feng, Z., Lugtenberg, M., Franse, C., Fang, X., Hu, S., Jin, C., & Raat, H. (2017). Risk factors and protective factors associated with incident or increase of frailty among community-dwelling older adults: A systematic review of longitudinal studies. *PloS one*, *12*(6), e0178383. https://doi.org/10.1371/journal.pone.0178383

Fernandes, T. G., Silva, K. R., Guerra, R. O., Parente, R. C. P., Borges, G. F., & Freire Junior, R. C. (2021). Influence of the Amazonian context on the frailty of older adults: A population-based study. *Archives of Gerontology and Geriatrics*, *93*, 104162. https://doi.org/10.1016/j.archger.2020.104162

Franse, C. B., van Grieken, A., Qin, L., Melis, R. J. F., Rietjens, J. A. C., & Raat, H. (2017). Socioeconomic inequalities in frailty and frailty components among community-dwelling older citizens. *PloS one*, *12*(11), e0187946. https://doi.org/10.1371/journal.pone.0187946

Freitag, S., & Schmidt, S. (2016). Psychosocial Correlates of Frailty in Older Adults. *Geriatrics (Basel)*, *1*(4), 26. https://doi.org/10.3390/geriatrics1040026

Fried, L. P., Tangen, C. M., Walston, J., Newman, A. B., Hirsch, C., Gottdiener, J., Seeman, T., Tracy, R., Kop, W. J., Burke, G., McBurnie, M. A., & Cardiovascular Health Study Collaborative Research, G. (2001). Frailty in older adults: evidence for a phenotype. *The Journals of Gerontology: Series A*, *56*(3), M146-156. https://doi.org/10.1093/gerona/56.3.m146

Fritz, H., Cutchin, M. P., Gharib, J., Haryadi, N., Patel, M., & Patel, N. (2020). Neighborhood Characteristics and Frailty: A Scoping Review. *Gerontologist*, *60*(4), e270-e285. https://doi.org/10.1093/geront/gnz072

Gale, C. R., Syddall, H. E., Cooper, C., Sayer, A. A., Bergman, H., & Brunner, E. J. (2012). Close relationships and risk of frailty: the Hertfordshire Cohort Study. *Journal of the American Geriatrics Society 60*(2), 390-392. https://doi.org/10.1111/j.1532-5415.2011.03799.x

Gale, C. R., Westbury, L., & Cooper, C. (2018). Social isolation and loneliness as risk factors for the progression of frailty: the English Longitudinal Study of Ageing. *Age and Ageing*, *47*(3), 392-397. https://doi.org/10.1093/ageing/afx188

Garcia-Esquinas, E., Navas-Acien, A., Perez-Gomez, B., & Artalejo, F. R. (2015). Association of lead and cadmium exposure with frailty in US older adults. *Environ Research*, *137*, 424-431. https://doi.org/10.1016/j.envres.2015.01.013

Garcia-Vigara, A., Cano, A., Fernandez-Garrido, J., Carbonell-Asins, J. A., Tarin, J. J., & Sanchez-Sanchez, M. L. (2022). Non-use of information and communication technology as a predictor of frailty in postmenopausal midlife and older women. *Maturitas*, *156*, 60-64. https://doi.org/10.1016/j.maturitas.2021.05.010

Gardiner, P. A., Mishra, G. D., & Dobson, A. J. (2016). The Effect of Socioeconomic Status Across Adulthood on Trajectories of Frailty in Older Women. *Journal of the American Medical Directors Association*, *17*(4), 372 e371-373. https://doi.org/10.1016/j.jamda.2015.12.090

Ge, F., & Kwon, S. (2023). How Neighborhood Structural and Individual Characteristics Affect Frailty Progression: Evidence from the China Health and Retirement Longitudinal Study. *The Journal of Nutrition, Health and Aging*, *27*(5), 362-370. https://doi.org/10.1007/s12603-023-1916-1

Ge, L., Yap, C. W., & Heng, B. H. (2022). Associations of social isolation, social participation, and loneliness with frailty in older adults in Singapore: a panel data analysis. *BMC Geriatrics*, *22*(1), 26. https://doi.org/10.1186/s12877-021-02745-2

Gobbens, R. J. J. (2019). Cross-sectional and Longitudinal Associations of Environmental Factors with Frailty and Disability in Older People. *Archives of Gerontology and Geriatrics*, *85*, 103901. https://doi.org/10.1016/j.archger.2019.103901

Gonzalez-Bautista, E., Roman-Perez, S., Manrique-Espinoza, B. S., Salinas-Rodriguez, A., & Santos-Luna, R. (2024). Frailty Was Associated With Atmospheric NO2 Levels: A Geospatial Approach. *The Journals of Gerontology: Series A*, *79*(9). https://doi.org/10.1093/gerona/glae168

Guo, X., Su, W., Wang, X., Hu, W., Meng, J., Ahmed, M. A., Qu, G., & Sun, Y. (2024a). Assessing the effects of air pollution and residential greenness on frailty in older adults: a prospective cohort study from China. *Environmental Science and Pollution Research*, *31*(6), 9091-9105. https://doi.org/10.1007/s11356-023-31741-9

Guo, Y., & Yang, F. (2024). Can Social Mobility Impact Frailty Trajectories of Chinese Adults in Later Life? A Nationwide Longitudinal Study. *Innovation in Aging*, *8*(2), igae003. https://doi.org/10.1093/geroni/igae003

Guo, Y. F., Ng, N., Kowal, P., Lin, H., Ruan, Y., Shi, Y., & Wu, F. (2022). Frailty Risk in Older Adults Associated With Long-Term Exposure to Ambient PM2.5 in 6 Middle-Income Countries. *The Journals of Gerontology: Series A*, *77*(5), 970-976. https://doi.org/10.1093/gerona/glac022

Guo, Z., Xue, H., Fan, L., Wu, D., Wang, Y., Chung, Y., Liao, Y., Ruan, Z., & Du, W. (2024b). Differential effects of size-specific particulate matter on frailty transitions among middle-aged and older adults in China: findings from the China Health and Retirement Longitudinal Study (CHARLS), 2015-2018. *International Health*, *16*(2), 182-193. https://doi.org/10.1093/inthealth/ihad033

Haider, S., Grabovac, I., Drgac, D., Mogg, C., Oberndorfer, M., & Dorner, T. E. (2020). Impact of physical activity, protein intake and social network and their combination on the development of frailty. *European Journal of Public Health*, *30*(2), 340-346. https://doi.org/10.1093/eurpub/ckz191

Hanlon, P., Politis, M., Wightman, H., Kirkpatrick, S., Jones, C., Khan, M., Bezzina, C., Mackinnon, S., Rennison, H., Wei, L., Vetrano, D. L., Blane, D. N., Dent, E., & Hoogendijk, E. O. (2024). Frailty and socioeconomic position: A systematic review of observational studies. *Ageing research reviews*, *100*, 102420. https://doi.org/10.1016/j.arr.2024.102420

He, Q., Chang, H. T., Wu, C. D., & Ji, J. S. (2022). Association between residential greenspace structures and frailty in a cohort of older Chinese adults. *Communications Medicine (Lond)*, *2*, 43. https://doi.org/10.1038/s43856-022-00093-9

Herr, M., Robine, J. M., Aegerter, P., Arvieu, J. J., & Ankri, J. (2015). Contribution of socioeconomic position over life to frailty differences in old age: comparison of life-course models in a French sample of 2350 old people. *Annals of epidemiology*, *25*(9), 674-680 e671. https://doi.org/10.1016/j.annepidem.2015.05.006

Hoogendijk, E. O., Suanet, B., Dent, E., Deeg, D. J., & Aartsen, M. J. (2016). Adverse effects of frailty on social functioning in older adults: Results from the Longitudinal Aging Study Amsterdam. *Maturitas*, *83*, 45-50. https://doi.org/10.1016/j.maturitas.2015.09.002

Hoogesteyn, K., Meijer, E., & Vrij, A. (2020). Examining witness interviewing environments. *Journal of Investigative Psychology and Offender Profiling*, *17*(3), 238-249. https://doi.org/10.1002/jip.1549

Hsu, H. C., & Chang, W. C. (2015). Trajectories of frailty and related factors of the older people in Taiwan. *Experimental Aging Research 41*(1), 104-114. https://doi.org/10.1080/0361073X.2015.978219

Hu, K., Keenan, K., Hale, J. M., & Borger, T. (2020). The association between city-level air pollution and frailty among the elderly population in China. *Health Place*, *64*, 102362. https://doi.org/10.1016/j.healthplace.2020.102362

Hu, S., Jin, C., & Li, S. (2022). Association between social capital and frailty and the mediating effect of health-promoting lifestyles in Chinese older adults: a cross-sectional study. *BMC Geriatrics*, *22*(1), 175. https://doi.org/10.1186/s12877-022-02815-z

Huang, J., Gui, Y., Wu, J., & Xie, Y. (2024). Causal effects of socioeconomic traits on frailty: a Mendelian randomization study. *Frontiers in Medicine*, *11*, 1344217. https://doi.org/10.3389/fmed.2024.1344217

Hulur, G., & Macdonald, B. (2020). Rethinking social relationships in old age: Digitalization and the social lives of older adults. *American Psychologist*, *75*(4), 554-566. https://doi.org/10.1037/amp0000604

Ikeda, T., Tsuboya, T., Aida, J., Matsuyama, Y., Koyama, S., Sugiyama, K., Kondo, K., & Osaka, K. (2019). Income and education are associated with transitions in health status among community-dwelling older people in Japan: the JAGES cohort study. *Family Practice*, *36*(6), 713-722. https://doi.org/10.1093/fampra/cmz022

Joanna Briggs Institute. (2017). *Checklist for analytical cross sectional studies*. https://jbi.global/sites/default/files/2019-05/JBI_Critical_Appraisal-Checklist_for_Analytical_Cross_Sectional_Studies2017_0.pdf

Iriarte, E., Cooley, S., Wisch, J., Erlandson, K. M., Ances, B. M., & Jankowski, C. (2025). Area Deprivation Index and Frailty Among Older People With HIV. *Journal of Applied Gerontology*, *44*(1), 44-51. https://doi.org/10.1177/07334648241262658

Jansen, S. J. (2013). Why is housing always satisfactory? A study into the impact of preference and experience on housing appreciation. *Social Indicators Research*, *113*(3), 785-805. https://doi.org/https://doi.org/10.1007/s11205-012-0114-9

Johansson, M. M., Natt, M., Peolsson, A., & Ohman, A. (2023). Frail community-dwelling older persons' everyday lives and their experiences of rehabilitation - a qualitative study. *Scandinavian Journal of Occupational Therapy*, *30*(1), 65-75. https://doi.org/10.1080/11038128.2022.2093269

John, P. D. S., Montgomery, P. R., & Tyas, S. L. (2013). Social position and frailty. *Canadian Journal on Aging/La Revue canadienne du vieillissement*, *32*(3), 250-259.

Keranen, N. S., Kangas, M., Immonen, M., Simila, H., Enwald, H., Korpelainen, R., & Jamsa, T. (2017). Use of Information and Communication Technologies Among Older People With and Without Frailty: A Population-Based Survey. *Journal of Medical Internet Research*, *19*(2), e29. https://doi.org/10.2196/jmir.5507

Kheifets, M., Goshen, A., Goldbourt, U., Witberg, G., Eisen, A., Kornowski, R., & Gerber, Y. (2022). Association of socioeconomic status measures with physical activity and subsequent frailty in older adults. *BMC Geriatrics*, *22*(1), 439. https://doi.org/10.1186/s12877-022-03108-1

Kim, D. H., Schneeweiss, S., Glynn, R. J., Lipsitz, L. A., Rockwood, K., & Avorn, J. (2018). Measuring Frailty in Medicare Data: Development and Validation of a Claims-Based Frailty Index. *The Journals of Gerontology: Series A*, *73*(7), 980-987. https://doi.org/10.1093/gerona/glx229

Kim, M. J., Seo, S. H., Seo, A. R., Kim, B. K., Lee, G. Y., Choi, Y. S., Kim, J. H., Kim, J. R., Kang, Y. S., Jeong, B. G., & Park, K. S. (2019). The Association of Perceived Neighborhood Walkability and Environmental Pollution With Frailty Among Community-dwelling Older Adults in Korean Rural Areas: A Cross-sectional Study. *Journal of Preventive Medicine and Public Health*, *52*(6), 405-415. https://doi.org/10.3961/jpmph.19.166

King, K. E., Fillenbaum, G. G., & Cohen, H. J. (2017). A Cumulative Deficit Laboratory Test-based Frailty Index: Personal and Neighborhood Associations. *Journal of the American Geriatrics Society 65*(9), 1981-1987. https://doi.org/10.1111/jgs.14983

Kojima, G., Taniguchi, Y., Kitamura, A., & Shinkai, S. (2018). Are the Kihon Checklist and the Kaigo-Yobo Checklist Compatible With the Frailty Index? *Journal of the American*

Medical Directors Association, *19*(9), 797-800 e792. https://doi.org/10.1016/j.jamda.2018.05.012

Kojima, G., Walters, K., Iliffe, S., Taniguchi, Y., & Tamiya, N. (2020). Marital Status and Risk of Physical Frailty: A Systematic Review and Meta-analysis. *Journal of the American Medical Directors Association*, *21*(3), 322-330. https://doi.org/10.1016/j.jamda.2019.09.017

Kwan, R. Y. C., Cheung, D. S. K., Lo, S. K. L., Ho, L. Y. W., Katigbak, C., Chao, Y. Y., & Liu, J. Y. W. (2019). Frailty and its association with the Mediterranean diet, life-space, and social participation in community-dwelling older people. *Geriatric Nursing*, *40*(3), 320-326. https://doi.org/10.1016/j.gerinurse.2018.12.011

Kwan, R. Y. C., Yeung, J. W. Y., Lee, J. L. C., & Lou, V. W. Q. (2023). The association of technology acceptance and physical activity on frailty in older adults during the COVID-19 pandemic period. *European Review of Aging and Physical Activity*, *20*(1), 24. https://doi.org/10.1186/s11556-023-00334-3

Lang, I. A., Hubbard, R. E., Andrew, M. K., Llewellyn, D. J., Melzer, D., & Rockwood, K. (2009). Neighborhood deprivation, individual socioeconomic status, and frailty in older adults. *Journal of the American Geriatrics Society* *57*(10), 1776-1780. https://doi.org/10.1111/j.1532-5415.2009.02480.x

Lawton, M. P., & Simon, B. (1968). The ecology of social relationships in housing for the elderly. *Gerontologist*, *8*(2), 108-115. https://doi.org/10.1093/geront/8.2.108

Lee, W. J., Liu, C. Y., Peng, L. N., Lin, C. H., Lin, H. P., & Chen, L. K. (2020). PM(2.5) air pollution contributes to the burden of frailty. *Scientific Reports*, *10*(1), 14478. https://doi.org/10.1038/s41598-020-71408-w

Li, Y., Wang, Y., & Morrow-Howell, N. (2021). Neighborhood Effects on the Health of Chinese Older Adults: Beyond the Rural and Urban Dichotomy. *Gerontologist*, *61*(3), 403-412. https://doi.org/10.1093/geront/gnaa081

Li, Y., Xue, Q. L., Odden, M. C., Chen, X., & Wu, C. (2020). Linking early life risk factors to frailty in old age: evidence from the China Health and Retirement Longitudinal Study. *Age and Ageing*, *49*(2), 208-217. https://doi.org/10.1093/ageing/afz160

Lin, B., & Zhu, J. (2020). Policy effect of the Clean Air Action on green development in Chinese cities. *Journal of Environmental Management*, *258*, 110036. https://doi.org/10.1016/j.jenvman.2019.110036

Liu, H., Chen, B., Li, Y., & Morrow-Howell, N. (2022). Neighborhood resources associated with frailty trajectories over time among community-dwelling older adults in China. *Health Place*, *74*, 102738. https://doi.org/10.1016/j.healthplace.2021.102738

Liu, H., Zhang, M., Chen, B., Huang, L., & Zhao, X. (2023). Links between life-course SES and frailty trajectory moderated by community environment resources: Person-environment Fit perspective. *Advances in Life Course Research*, *58*, 100580. https://doi.org/10.1016/j.alcr.2023.100580

Liu, M., Zeeshan, M., Sun, T., Hu, X., Nie, Z., Dong, H., Dong, G., & Ou, Y. (2024). Association of Air Quality Improvement and Frailty Progression: A National Study across China. *Toxics*, *12*(7). https://doi.org/10.3390/toxics12070464

Liu, Y., Meng, H., Tu, N., & Liu, D. (2020). The Relationship Between Health Literacy, Social Support, Depression, and Frailty Among Community-Dwelling Older Patients With

Hypertension and Diabetes in China. *Frontiers in Public Health*, *8*, 280. https://doi.org/10.3389/fpubh.2020.00280

Lyu, W., Tanaka, T., Son, B. K., Akishita, M., & Iijima, K. (2022). Associations of multi-faceted factors and their combinations with frailty in Japanese community-dwelling older adults: Kashiwa cohort study. *Archives of Gerontology and Geriatrics*, *102*, 104734. https://doi.org/10.1016/j.archger.2022.104734

Maharani, A., Sinclair, D. R., Chandola, T., Bower, P., Clegg, A., Hanratty, B., Nazroo, J., Pendleton, N., Tampubolon, G., Todd, C., Wittenberg, R., O'Neill, T. W., & Matthews, F. E. (2023). Household wealth, neighbourhood deprivation and frailty amongst middle-aged and older adults in England: a longitudinal analysis over 15 years (2002-2017). *Age and Ageing*, *52*(3). https://doi.org/10.1093/ageing/afad034

Maltby, J., Hunt, S. A., Ohinata, A., Palmer, E., & Conroy, S. (2020). Frailty and Social Isolation: Comparing the Relationship between Frailty and Unidimensional and Multifactorial Models of Social Isolation. *Journal of Aging and Health*, *32*(10), 1297-1308. https://doi.org/10.1177/0898264320923245

Manrique-Espinoza, B., Salinas-Rodriguez, A., Salgado de Snyder, N., Moreno-Tamayo, K., Gutierrez-Robledo, L. M., & Avila-Funes, J. A. (2016). Frailty and Social Vulnerability in Mexican Deprived and Rural Settings. *Journal of Aging and Health*, *28*(4), 740-752. https://doi.org/10.1177/0898264315609909

Martins, B. A., Visvanathan, R., Barrie, H. R., Huang, C. H., Matsushita, E., Okada, K., Satake, S., Edwards, S., Uno, C., & Kuzuya, M. (2021). Built Environment and Frailty: Neighborhood Perceptions and Associations With Frailty, Experience From the Nagoya Longitudinal Study. *Journal of Applied Gerontology*, *40*(6), 609-619. https://doi.org/10.1177/0733464820912663

Mena, C., Ormazabal, Y., Fuentes, E., & Palomo, I. (2020). Impacts of Physical Environment Perception on the Frailty Condition in Older People. *Geospatial Health*, *15*(1). https://doi.org/10.4081/gh.2020.888

Mendonca, N., Kingston, A., Yadegarfar, M., Hanson, H., Duncan, R., Jagger, C., & Robinson, L. (2020). Transitions between frailty states in the very old: the influence of socioeconomic status and multi-morbidity in the Newcastle 85+ cohort study. *Age and Ageing*, *49*(6), 974-981. https://doi.org/10.1093/ageing/afaa054

Mitnitski, A. B., Mogilner, A. J., & Rockwood, K. (2001). Accumulation of deficits as a proxy measure of aging. *The Scientific World Journal*, *1*, 323-336. https://doi.org/10.1100/tsw.2001.58

Mitsutake, S., Ishizaki, T., Yokoyama, Y., Nishi, M., Koohsari, M. J., Oka, K., Yano, S., Abe, T., & Kitamura, A. (2021). Do Walking-Friendly Built Environments Influence Frailty and Long-Term Care Insurance Service Needs? *Sustainability*, *13*(10). https://doi.org/10.3390/su13105632

Mooney, C. J., Elliot, A. J., Douthit, K. Z., Marquis, A., & Seplaki, C. L. (2018). Perceived Control Mediates Effects of Socioeconomic Status and Chronic Stress on Physical Frailty: Findings From the Health and Retirement Study. *The Journals of Gerontology: Series B*, *73*(7), 1175-1184. https://doi.org/10.1093/geronb/gbw096

Mori, Y., Tsuji, T., Watanabe, R., Hanazato, M., Chen, Y. R., & Kondo, K. (2023). Built Environments and Frailty in Older Adults: The JAGES Longitudinal Study Using Mediation Analysis. *Journal of the American Medical Directors Association*, *24*(11), 1677-1682. https://doi.org/10.1016/j.jamda.2023.06.023

Mori, Y., Tsuji, T., Watanabe, R., Hanazato, M., Miyazawa, T., & Kondo, K. (2022). Built environments and frailty in older adults: A three-year longitudinal JAGES study. *Archives of Gerontology and Geriatrics*, *103*, 104773. https://doi.org/10.1016/j.archger.2022.104773

Mumken, S. A., Alonso-Perez, E., Haeger, C., O'Sullivan, J. L., Xue, Q. L., Lech, S., Herrmann, W. J., & Gellert, P. (2024). Prevention of frailty in relation with social out-of-home activities in older adults: results from the Survey of Health, Ageing, and Retirement in Europe. *European Journal of Ageing*, *21*(1), 35. https://doi.org/10.1007/s10433-024-00829-7

Nakajima, Y., Schmidt, S. M., Malmgren Fange, A., Ono, M., & Ikaga, T. (2019). Relationship between Perceived Indoor Temperature and Self-Reported Risk for Frailty among Community-Dwelling Older People. *International Journal of Environmental Research and Public Health*, *16*(4). https://doi.org/10.3390/ijerph16040613

Niedoba, S., & Oswald, F. (2024). Person–Environment Exchange Processes in Transition into Dementia: A Scoping Review. *Gerontologist*, *64*(2). https://doi.org/10.1093/geront/gnad034

Nishimura, A., Masuda, C., Murauchi, C., Ishii, M., Murata, Y., Kawasaki, T., Azuma, M., Arai, H., & Harashima, S. I. (2024). Regional differences in frailty among older adults with type 2 diabetes: a multicenter cross-sectional study in Japan. *BMC Geriatrics*, *24*(1), 688. https://doi.org/10.1186/s12877-024-05223-7

Noguchi, T., Murata, C., Hayashi, T., Watanabe, R., Saito, M., Kojima, M., Kondo, K., & Saito, T. (2022). Association between community-level social capital and frailty onset among older adults: a multilevel longitudinal study from the Japan Gerontological Evaluation Study (JAGES). *Journal of Epidemiology and Community Health*, *76*(2), 182-189. https://doi.org/10.1136/jech-2021-217211

Noguchi, T., & Shang, E. (2024). Role of Individual Social Capital in the Association of Physical Frailty With Functional Ability Among Older Adults. *Journal of the American Medical Directors Association*, *25*(7), 105024. https://doi.org/10.1016/j.jamda.2024.105024

Nyende, A., Ellis-Hill, C., & Mantzoukas, S. (2023). A Sense of Control and Wellbeing in Older People Living with Frailty: A Scoping Review. *Journal of Gerontological Social Work*, *66*(8), 1043-1072. https://doi.org/10.1080/01634372.2023.2206438

Ocampo-Chaparro, J. M., Reyes-Ortiz, C. A., Castro-Florez, X., & Gomez, F. (2019). Frailty in older adults and their association with social determinants of Health. The SABE Colombia Study. *Colombia Médica (Cali)*, *50*(2), 89-101. https://doi.org/10.25100/cm.v50i2.4121

Oswald, F., & Wahl, H.-W. (2005). *Home and identity in late life: International perspectives*.

Oswald, F., Wahl, H.-W., Wanka, A., & Chaudhury, H. (2024). Theorizing place and aging: Enduring and novel issues in environmental gerontology. In *Handbook on aging and place* (pp. 37-60). Edward Elgar Publishing.

Page, M. J., McKenzie, J. E., Bossuyt, P. M., Boutron, I., Hoffmann, T. C., Mulrow, C. D., Shamseer, L., Tetzlaff, J. M., Akl, E. A., Brennan, S. E., Chou, R., Glanville, J., Grimshaw, J. M., Hrobjartsson, A., Lalu, M. M., Li, T., Loder, E. W., Mayo-Wilson, E., McDonald, S., . . . Moher, D. (2021). The PRISMA 2020 statement: an updated guideline for reporting systematic reviews. *BMJ*, *372*, n71. https://doi.org/10.1136/bmj.n71

Peace, S., Wahl, H.-W., Mollenkopf, H., & Oswald, F. (2007). Environment and ageing. *Ageing and Society*, 209-234.
Peek, M. K., Howrey, B. T., Ternent, R. S., Ray, L. A., & Ottenbacher, K. J. (2012). Social support, stressors, and frailty among older Mexican American adults. *The Journals of Gerontology: Series B*, *67*(6), 755-764.
Perez-Hernandez, B., Lopez-Garcia, E., Graciani, A., Ayuso-Mateos, J. L., Rodriguez-Artalejo, F., & Garcia-Esquinas, E. (2018). Housing conditions and risk of physical function limitations: a prospective study of community-dwelling older adults. *Journal of Public Health (Oxf)*, *40*(3), e252-e259. https://doi.org/10.1093/pubmed/fdy004
Peters, M. D. J., Marnie, C., Tricco, A. C., Pollock, D., Munn, Z., Alexander, L., McInerney, P., Godfrey, C. M., & Khalil, H. (2020). Updated methodological guidance for the conduct of scoping reviews. *JBI Evidence Synthesis*, *18*(10), 2119-2126. https://doi.org/10.11124/JBIES-20-00167
Pollak, C., Verghese, J., & Blumen, H. M. (2024). Difference-Making Pathways to Frailty Through Social Factors: A Configurational Analysis. *Gerontologist*, *64*(6). https://doi.org/10.1093/geront/gnad173
Portegijs, E., Rantakokko, M., Viljanen, A., Sipila, S., & Rantanen, T. (2016). Is frailty associated with life-space mobility and perceived autonomy in participation outdoors? A longitudinal study. *Age and Ageing*, *45*(4), 550-553. https://doi.org/10.1093/ageing/afw072
Riley, A. R. (2018). Neighborhood Disadvantage, Residential Segregation, and Beyond-Lessons for Studying Structural Racism and Health. *Journal of Racial and Ethnic Health Disparities*, *5*(2), 357-365. https://doi.org/10.1007/s40615-017-0378-5
Risbridger, S., Walker, R., Gray, W. K., Kamaruzzaman, S. B., Ai-Vyrn, C., Hairi, N. N., Khoo, P. L., & Pin, T. M. (2022). Social Participation's Association with Falls and Frailty in Malaysia: A Cross-Sectional Study. *The Journal of frailty and Aging*, *11*(2), 199-205. https://doi.org/10.14283/jfa.2021.31
Rowles, G. D., & Cutchin, M. (2025). Aging, place, and the life course of societies. *Journal of Aging Studies*, *75*, 101373. https://doi.org/10.1016/j.jaging.2025.101373
Schwartz, L. F., Dhaduk, R., Howell, C. R., Brinkman, T. M., Ehrhardt, M. J., Delaney, A., Srivastava, D. K., Lanctot, J. Q., Armstrong, G. T., Robison, L. L., Hudson, M. M., Ness, K. K., & Henderson, T. O. (2023). The Association of Neighborhood Characteristics and Frailty in Childhood Cancer Survivors: A Report from the St. Jude Lifetime Cohort Study. *Cancer Epidemiology, Biomarkers & Prevention*, *32*(8), 1021-1029. https://doi.org/10.1158/1055-9965.EPI-22-1322
Serrat, R., Scharf, T., Villar, F., & Gomez, C. (2020). Fifty-Five Years of Research Into Older People's Civic Participation: Recent Trends, Future Directions. *Gerontologist*, *60*(1), e38-e51. https://doi.org/10.1093/geront/gnz021
Seo, Y., Kim, M., Shim, H., & Won, C. W. (2021). Differences in the Association of Neighborhood Environment With Physical Frailty Between Urban and Rural Older Adults: The Korean Frailty and Aging Cohort Study (KFACS). *Journal of the American Medical Directors Association*, *22*(3), 590-597 e591. https://doi.org/10.1016/j.jamda.2020.09.044
Shah, R., Carandang, R. R., Shibanuma, A., Ong, K. I. C., Kiriya, J., & Jimba, M. (2021). Understanding frailty among older people living in old age homes and the community in

Nepal: A cross-sectional study. *PloS one*, *16*(4), e0251016. https://doi.org/10.1371/journal.pone.0251016

Shakya, S., Silva, S. G., McConnell, E. S., McLaughlin, S. J., & Cary, M. P., Jr. (2024). Psychosocial stressors associated with frailty in community-dwelling older adults in the United States. *Journal of the American Geriatrics Society 72*(4), 1088-1099. https://doi.org/10.1111/jgs.18821

Shiau, M. H., Hurng, B. S., Wang, Y. W., & Yeh, C. J. (2023). Association between socioeconomic position trajectories and frailty among elderly people in Taiwan. *Archives of Gerontology and Geriatrics*, *104*, 104824. https://doi.org/10.1016/j.archger.2022.104824

Shin, J., & Choi, J. (2021). Frailty Related to the Exposure to Particulate Matter and Ozone: The Korean Frailty and Aging Cohort Study. *International Journal of Environmental Research and Public Health*, *18*(22). https://doi.org/10.3390/ijerph182211796

Shinkai, S., Watanabe, N., Yoshida, H., Fujiwara, Y., Nishi, M., Fukaya, T., Lee, S., Kim, M. J., Ogawa, K., & Murayama, H. (2013). Validity of the" Kaigo-Yobo Check-List" as a frailty index. *Japanese Journal of Public Health*, *60*(5), 262-274.

Shiratsuchi, D., Makizako, H., Akaida, S., Tateishi, M., Hirano, H., Iijima, K., Yamada, M., Kojima, N., Obuchi, S., Fujiwara, Y., Murayama, H., Nishita, Y., Jeong, S., Otsuka, R., Abe, T., & Suzuki, T. (2024). Associations between information and communication technology use and frailty in community-dwelling old-old adults: results from the ILSA-J. *European Geriatric Medicine*, *15*(3), 621-627. https://doi.org/10.1007/s41999-024-00979-y

Solar, O., & Irwin, A. (2010). *A conceptual framework for action on the social determinants of health*. World Health Organization.

Song, Q., Dai, M., Lin, T., Zhao, Y., Peng, X., Liang, R., Su, Q., & Yue, J. (2023). Biomass fuel usage for cooking and frailty among older adults in China: a population-based cohort study. *Frontiers in Public Health*, *11*, 1122243. https://doi.org/10.3389/fpubh.2023.1122243

Su, Y., Hamatani, M., Yuki, M., Ogawa, N., & Kawahara, K. (2024). Frailty and social isolation before and during the coronavirus disease 2019 pandemic among older adults: A path analysis. *Journal of Advanced Nursing*, *80*(5), 1902-1913. https://doi.org/10.1111/jan.15971

Szanton, S. L., Seplaki, C. L., Thorpe, R. J., Jr., Allen, J. K., & Fried, L. P. (2010). Socioeconomic status is associated with frailty: the Women's Health and Aging Studies. *Journal of Epidemiology and Community Health*, *64*(1), 63-67. https://doi.org/10.1136/jech.2008.078428

Takatori, K., & Matsumoto, D. (2021). Social factors associated with reversing frailty progression in community-dwelling late-stage elderly people: An observational study. *PloS one*, *16*(3), e0247296. https://doi.org/10.1371/journal.pone.0247296

Theou, O., Brothers, T. D., Pena, F. G., Mitnitski, A., & Rockwood, K. (2014). Identifying common characteristics of frailty across seven scales. *Journal of the American Geriatrics Society 62*(5), 901-906. https://doi.org/10.1111/jgs.12773

Thinuan, P., Siviroj, P., Barry, C. D., Gordon, S. C., Lerttrakarnnon, P., & Lorga, T. (2020). Narratives of older persons' frailty and physical activity in relation to environmental landscapes and time. *International Journal of Older People Nursing*, *15*(2), e12298. https://doi.org/10.1111/opn.12298

Tricco, A. C., Lillie, E., Zarin, W., O'Brien, K. K., Colquhoun, H., Levac, D., Moher, D., Peters, M. D. J., Horsley, T., Weeks, L., Hempel, S., Akl, E. A., Chang, C., McGowan, J., Stewart, L., Hartling, L., Aldcroft, A., Wilson, M. G., Garritty, C., . . . Straus, S. E. (2018). PRISMA Extension for Scoping Reviews (PRISMA-ScR): Checklist and Explanation. *Annals of Internal Medicine*, *169*(7), 467-473. https://doi.org/10.7326/M18-0850

Tsubota-Utsugi, M., Yonekura, Y., Tanno, K., Nozue, M., Shimoda, H., Nishi, N., Sakata, K., Kobayashi, S., & study, R. (2018). Association between health risks and frailty in relation to the degree of housing damage among elderly survivors of the great East Japan earthquake. *BMC Geriatrics*, *18*(1), 133. https://doi.org/10.1186/s12877-018-0828-x

Uccheddu, D., Gauthier, A. H., Steverink, N., & Emery, T. (2019). Gender and socioeconomic inequalities in health at older ages across different European welfare clusters: Evidence from SHARE data, 2004–2015. *European Sociological Review*, *35*(3), 346-362. https://doi.org/10.1093/esr/jcz007

Uno, C., Okada, K., Matsushita, E., Satake, S., & Kuzuya, M. (2021). Friendship-related social isolation is a potential risk factor for the transition from robust to prefrailty among healthy older adults: a 1-year follow-up study. *European Geriatric Medicine*, *12*(2), 285-293. https://doi.org/10.1007/s41999-020-00422-y

Van der Linden, B. W. A., Cheval, B., Sieber, S., Orsholits, D., Guessous, I., Stringhini, S., Gabriel, R., Aartsen, M., Blane, D., Courvoisier, D., Burton-Jeangros, C., Kliegel, M., & Cullati, S. (2020). Life Course Socioeconomic Conditions and Frailty at Older Ages. *The Journals of Gerontology: Series B*, *75*(6), 1348-1357. https://doi.org/10.1093/geronb/gbz018

Veronese, N., Maniscalco, L., Matranga, D., Lacca, G., Dominguez, L. J., & Barbagallo, M. (2023). Association Between Pollution and Frailty in Older People: A Cross-Sectional Analysis of the UK Biobank. *Journal of the American Medical Directors Association*, *24*(4), 475-481 e473. https://doi.org/10.1016/j.jamda.2022.12.027

Wahl, H. W., & Gerstorf, D. (2020). Person-Environment Resources for Aging Well: Environmental Docility and Life Space as Conceptual Pillars for Future Contextual Gerontology. *Gerontologist*, *60*(3), 368-375. https://doi.org/10.1093/geront/gnaa006

Wahl, H.-W., & Gerstorf, D. (2018). A conceptual framework for studying COntext Dynamics in Aging (CODA). *Developmental Review*, *50*, 155-176. https://doi.org/https://doi.org/10.1016/j.dr.2018.09.003

Wang, H. Y., Huang, Y., Zhou, M. R., Jiang, H. Y., Zong, Y. H., Zhu, X. H., & Sun, X. (2024). Socioeconomic support, quality of life, and prognosis of frailty among the older adults. *Health Care Science*, *3*(2), 101-113. https://doi.org/10.1002/hcs2.88

Wang, J., & Hulme, C. (2021). Frailty and socioeconomic status: a systematic review. *Journal of Public Health Research*, *10*(3). https://doi.org/10.4081/jphr.2021.2036

Wang, L., Xie, S., Hu, X., Li, J., He, S., Gao, J., & Wang, Z. (2023). Social capital, depressive symptomatology, and frailty among older adults in the western areas of China. *PloS one*, *18*(10), e0292236. https://doi.org/10.1371/journal.pone.0292236

Wang, Q. (2023). Social contexts and cross-national differences in association between adverse childhood experiences and frailty index. *SSM - Population Health*, *22*, 101408. https://doi.org/10.1016/j.ssmph.2023.101408

Wells, G. A., Shea, B., O'Connell, D., Peterson, J., Welch, V., Losos, M., & Tugwell, P. (2000). *The Newcastle-Ottawa Scale (NOS) for assessing the quality of nonrandomised studies in*

meta-analyses. Ottawa: Ottawa Hospital Research Institute. https://web.archive.org/web/20210716121605id_/www3.med.unipmn.it/dispense_ebm/2009-2010/Corso%20Perfezionamento%20EBM_Faggiano/NOS_oxford.pdf

Welstead, M., Luciano, M., Russ, T. C., & Muniz-Terrera, G. (2022). Heterogeneity of Frailty Trajectories and Associated Factors in the Lothian Birth Cohort 1936. *Gerontology*, *68*(8), 861-868. https://doi.org/10.1159/000519240

Wiles, J., & Coleman, T. (2024). Home and aging. In *Handbook on Aging and Place* (pp. 183-200). Edward Elgar Publishing.

Williams, G. R., Fowler, M., Giri, S., Dai, C., Harmon, C., Al-Obaidi, M., Stephenson, C., Bona, K., Landier, W., Bhatia, S., & Wolfson, J. (2023). Association of unmet basic resource needs with frailty and quality of life among older adults with cancer-Results from the CARE registry. *Cancer Medicine*, *12*(12), 13846-13855. https://doi.org/10.1002/cam4.6038

Woo, J., Goggins, W., Sham, A., & Ho, S. C. (2005). Social determinants of frailty. *Gerontology*, *51*(6), 402-408. https://doi.org/10.1159/000088705

Wu, D., Guo, Z., Xue, H., Fan, L., Liao, Y., Nyame, L., Cui, M., Tian, Y., Ruan, Z., & Du, W. (2024). Association between City-Level Particulate Matter Exposure and Frailty among Middle-Aged and Older Adults in China. *Gerontology*, *70*(10), 1074-1087. https://doi.org/10.1159/000539517

Xu, J., Chen, Y., Wang, Y., Gao, J., & Huang, L. (2022). Association between Age-Friendliness of Communities and Frailty among Older Adults: A Multilevel Analysis. *International Journal of Environmental Research and Public Health, 19*(12). https://doi.org/10.3390/ijerph19127528

Xue, Q. L., Fried, L. P., Glass, T. A., Laffan, A., & Chaves, P. H. M. (2007). Life-Space Constriction, Development of Frailty, and the Competing Risk of Mortality: The Women's Health and Aging Study I. *American Journal of Epidemiology*, *167*(2), 240-248. https://doi.org/10.1093/aje/kwm270

Yan, Y., Cai, L., & Lu, N. (2022). Childhood experiences and frailty trajectory among middle-aged and older adults in China. *European Journal of Ageing*, *19*(4), 1601-1615. https://doi.org/10.1007/s10433-022-00746-7

Ye, B., Gao, J., & Fu, H. (2018). Associations between lifestyle, physical and social environments and frailty among Chinese older people: a multilevel analysis. *BMC Geriatrics*, *18*(1), 314. https://doi.org/10.1186/s12877-018-0982-1

Young, A. C., Glaser, K., Spector, T. D., & Steves, C. J. (2016). The Identification of Hereditary and Environmental Determinants of Frailty in a Cohort of UK Twins. *Twin Research and Human Genetics*, *19*(6), 600-609. https://doi.org/10.1017/thg.2016.72

Yu, R., Tong, C., Leung, J., & Woo, J. (2020). Socioeconomic Inequalities in Frailty in Hong Kong, China: A 14-Year Longitudinal Cohort Study. *International Journal of Environmental Research and Public Health*, *17*(4). https://doi.org/10.3390/ijerph17041301

Yu, R., Wang, D., Leung, J., Lau, K., Kwok, T., & Woo, J. (2018). Is Neighborhood Green Space Associated With Less Frailty? Evidence From the Mr. and Ms. Os (Hong Kong) Study. *Journal of the American Medical Directors Association*, *19*(6), 528-534. https://doi.org/10.1016/j.jamda.2017.12.015

Zhou, H., Yu, W., Zhao, K., Shan, H., Zhou, S., Zhang, Y., Wang, H., & Wei, S. (2024). Adaptative thermal comfort analysis in the elderly based on Fried frailty classification in

residential buildings during summer. *Building and Environment*, *252*. https://doi.org/10.1016/j.buildenv.2024.111262

Zimmermann, J., Hansen, S., & Wagner, M. (2021). Home environment and frailty in very old adults. *Zeitschrift für Gerontologie und Geriatrie 54*(Suppl 2), 114-119. https://doi.org/10.1007/s00391-021-01969-6
